# Enhancing the Cumulene Character of One‐Dimensional Acetylene‐Based Systems by Stimuli‐Induced Planarization of Their Two Pro‐diradicaloid Cyclopenta[*h,i*]aceanthrylene Units

**DOI:** 10.1002/anie.202419832

**Published:** 2024-11-21

**Authors:** Álvaro Corrochano Fernández, Samara Medina Rivero, Sumit Chaurasia, Tomás Torres, Juan Casado, Giovanni Bottari

**Affiliations:** ^1^ Departamento de Química Orgánica Universidad Autónoma de Madrid, Campus de Cantoblanco 28049 Madrid Spain; ^2^ IMDEA-Nanociencia, Campus de Cantoblanco 28049 Madrid Spain; ^3^ Institute for Advanced Research in Chemical Sciences (IAdChem) Universidad Autónoma de Madrid 28049 Madrid Spain; ^4^ Department of Physical Chemistry University of Málaga, Andalucia-Tech, Campus de Teatinos s/n 29071 Málaga Spain

**Keywords:** acetylene, cooperative supramolecular polymerization, cyclopenta[*h,i*]aceanthrylene, cumulene, pro-diradicaloid species

## Abstract

Acetylene/polyynes −(C≡C−)_*n*_ and cumulenes =(C=)_*n*_ are connectors widely used for the realization of one‐dimensional (1D) π‐conjugates. Although both π‐moieties are constituted by *sp* carbon atoms, their different bond connectivity confers distinct physicochemical properties to the resulting systems. In this context, while many acetylene/polyyne‐ and cumulene‐based derivatives have been prepared and studied, no reports have tackled the possibility to reversibly alter the acetylene/polyyne‐cumulene electronic character of these derivatives using mild conditions. Herein, we present a novel approach to enhance the cumulene character of 1D acetylene‐based conjugates consisting in the preparation of derivatives featuring an acetylene moiety connecting two pro‐diradicaloid species, namely cyclopenta[*h,i*]aceanthrylene (CPA), at their pro‐radical positions. A thoughtful spectroscopic study of the prepared dimers, complemented by theoretical calculations, suggest a high π‐electronic delocalization of the pro‐diradicaloid CPAs through the central acetylene spacer upon the dimers’ planarization which, in turn, increases the cumulenic character of the acetylenic π‐bridge, a feature enhanced for one of the two dimers at low temperature and in methylcyclohexane due to an aggregation‐induced planarization process. We reckon that the proposed approach offers an interesting avenue towards the realization of 1D systems which cumulenic character of the acetylenic π‐connector could be altered in response to external stimuli.

## Graphical abstract

Connecting two pro‐diradicaloid species, namely cyclopenta[*h,i*]aceanthrylene, through an acetylene bridge has been shown to increase the cumulene character in one‐dimensional acetylene‐based conjugates. Spectroscopic analysis and calculations indicate significant π‐electronic delocalization during the low temperature‐promoted planarization of the conjugates, which in turn enhances the cumulenic character of the acetylenic π‐bridge.

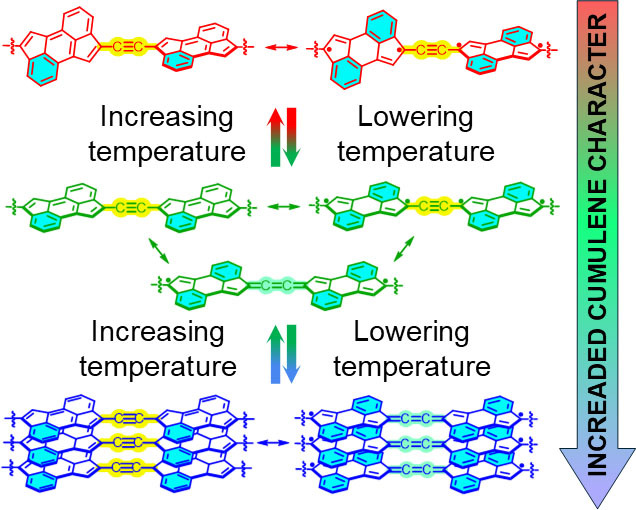

## Introduction

π‐Conjugated, one‐dimensional (1D) molecules are compounds featuring a continuous network of overlapping π‐orbitals which facilitates the delocalization of π‐electrons along the backbone.[^1^, ^2^, ^3^, ^4^] As a result of their extended π‐conjugation, these 1D systems present several interesting electronic properties.[^5^, ^6^, ^7^, ^8^, ^9^] On one hand, the appearance of size‐dependent band‐like states and wavefunction confinement confer them with the capability to absorb and emit light over a wide range of wavelengths, making them attractive materials for optoelectronic applications such as organic lasers, photodetectors, and organic light‐emitting diodes (OLEDs).[^10^, ^11^] On the other hand, the 1D nature of these compounds allows for anisotropic and directional charge transport along the elongated axis, prompting their use as single‐molecule electronic components in integrated circuits.[^12^, ^13^]

**Figure 1:**
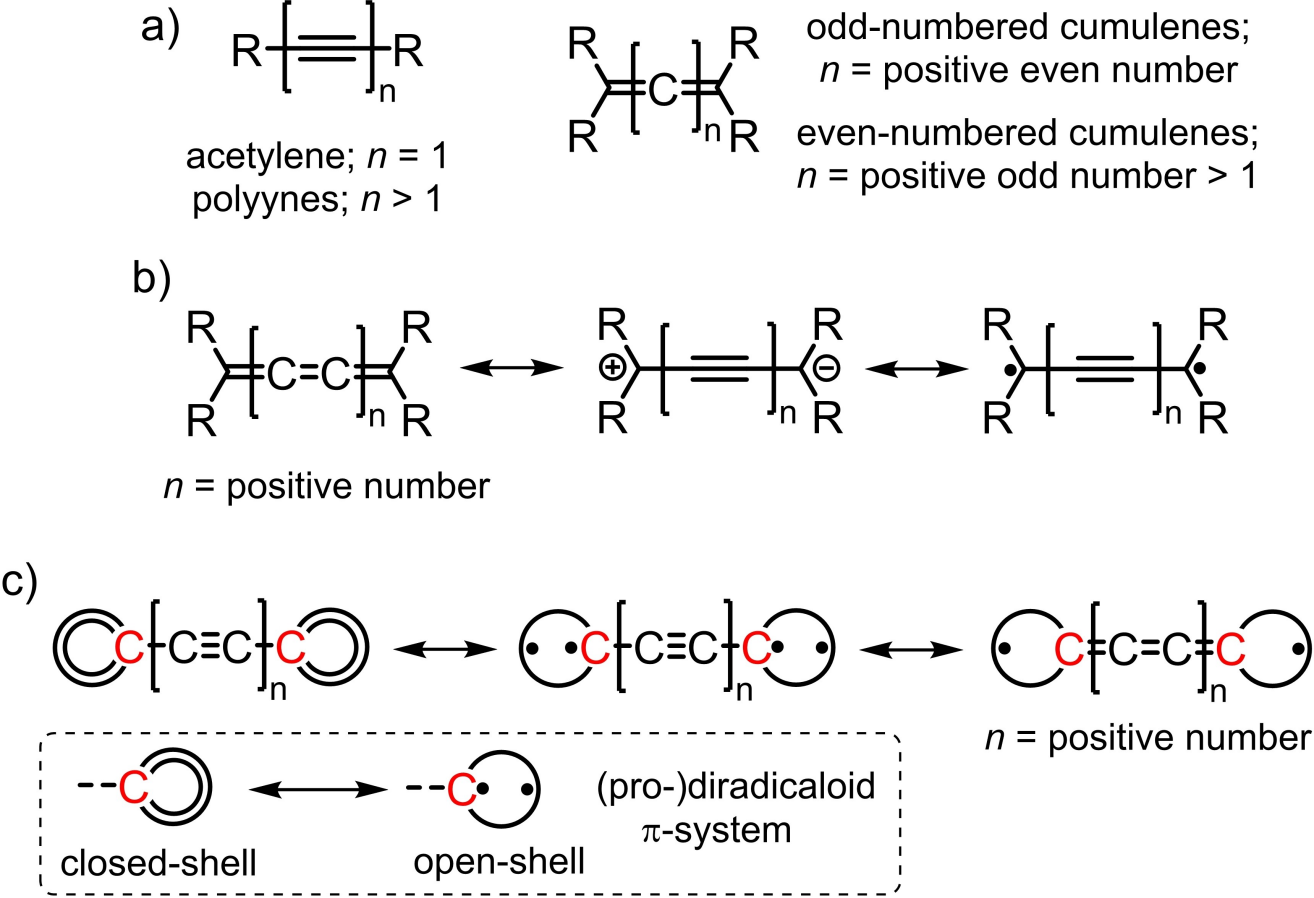
a) Generic molecular structure of acetylene/polyynes and [*n*]cumulenes. b) Closed‐shell cumulene‐type (left), charge‐separated (middle), and diradical open‐shell acetylene/polyyne‐type (right) canonical forms in odd‐numbered cumulenes. c) Canonical forms of a generic acetylene/polyyne‐based 1D compound incorporating two terminal (pro‐)diradicaloid π‐systems. The two carbon atoms coloured in red are those shared between the central acetylene/polyyne spacer and the two adjacent (pro‐)diradicaloid species, each of them sustaining a (partial) radical character.

Fostered by their potential applications in the fields of chemistry, materials science, and electronics, the design and synthesis of π‐conjugated 1D molecules have rapidly evolved during the last decades, leading to the realization of a wide range of novel materials with tailored properties.[^3^, ^4^] From the structural point of view, a common feature shared by many of these 1D π‐conjugated systems is the presence, in their molecular skeleton, of π‐bridges that can act as inter‐connectors between 2D polycyclic π‐conjugated units or end‐capped π‐conjugated moieties. Focusing on the π‐connectors employed for the realization of such 1D molecules, acetylene/polyynes^[14]^ and [*n*]cumulenes (where *n* is the number of cumulated double bonds in a chain constructed of *n*+1 carbon atoms)^[15]^ are, arguably, among the most widely used building blocks (Figure 1a). While both 1D π‐bridges are constituted by *sp* carbon atoms, in the acetylene/polyynes, such carbon atoms are connected by an alternating sequence of single and triple bonds, whereas in the [*n*]cumulenes, the π‐conjugation is provided by the presence of consecutive double bonds. Such different bond connectivity, beside conferring distinct physicochemical properties to the acetylene/polyyne‐ and cumulene‐based systems, requires the use of dissimilar synthetic strategies for the preparation of these 1D conjugates.^[16]^ Nonetheless, it has been hypothesized that, for odd‐numbered cumulenes, besides the closed‐shell cumulene‐type electronic configuration (Figure 1b, left), acetylene/polyyne‐type canonical forms showing a charge‐separated (Figure 1b, middle) and an open‐shell diradical character (Figure 1b, right) should also be considered.^[17]^ In this context, it has been theoretically predicted a gradual transition from a cumulene‐type to a polyyne‐type electronic structure upon increasing the length of the 1D π‐conjugated wire.^[18]^ Such acetylene/polyyne species have also been postulated as short‐lived intermediates in order to explain the relatively low rotation barrier (i.e., few tens of kcal mol^−1^) about the cumulene axis observed in odd‐numbered cumulenes.^[19]^ More recently, such cumulene‐to‐polyyne transition has been realized by preparing a [5]cumulene end‐capped by two phenalenyl moieties able to stabilize radicals.^[20]^

Interestingly, the reverse process, namely the polyyne‐to‐cumulene transition, has also been achieved in few cases using “harsh” conditions such as applying UV light photoexcitation at 78 K,^[21]^ strong electric fields,^[22]^ or chemical reduction of polyynes.^[23]^ In all those cases, the formation of metastable intermediates presenting a certain degree of cumulene character was detected. However, to the best of our knowledge, no reports have appeared documenting the possibility to control, even partially, the acetylene/polyyne‐to‐cumulene transformation in a reversible fashion and using “mild” conditions.

With the aim of identifying potential 1D π‐systems which could undergo such controlled electronic rearrangement, we turned our attention back to the diradical acetylene/polyynes canonical forms seen for odd‐numbered cumulenes (see above). While it is reasonable to assume that, in such systems, the acetylene/ polyyne open‐shell electronic configuration should have a smaller contribution to the molecule resonance hybrid compared to its closed‐shell cumulene analogue, we reasoned that such situation could be altered by flanking the acetylene/polyyne unit with two π‐conjugated systems showing a (pro‐)diradicaloid character (Figure 1c). We hypothesize that, in such 1D π‐conjugated molecules, engineering the two (pro‐)radical carbon atoms shared between the central acetylene/polyyne spacer and the two adjacent (pro‐)diradicaloid systems (coloured in red in Figure 1c) could synergistically increase the electronic communication between the two molecular halves thus promoting, under some conditions, the acetylene/polyyne‐to‐cumulene transition.

**Figure 2:**
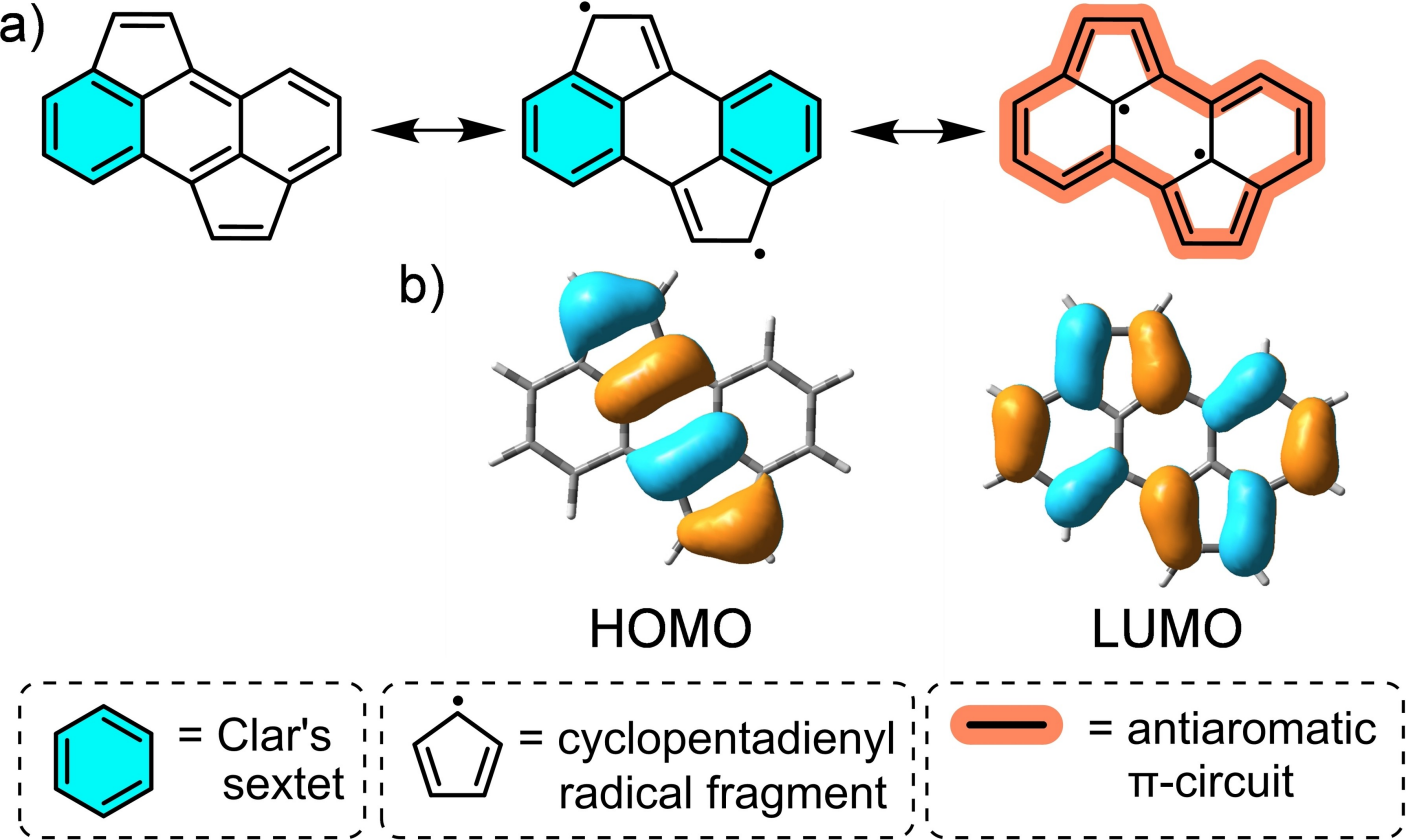
a) Three of the possible canonical forms of CPA. b) Orbital topology of the HOMO and LUMO of an unsubstituted CPA calculated at the B3LYP/6‐31G** level (isovalue=0.03). The two FMOs show important similarity with the canonical forms in a) represented above them.

Based on this hypothesis, while searching for a π‐conjugated system showing such (pro‐)diradicaloid character and that could be used as “capping” unit for an acetylene/polyyne π‐conjugated wire, we come across to cyclopenta[*h,i*]aceanthrylene (CPA) (Figure 2a).^[24]^ CPA is a planar polyaromatic hydrocarbon (PAH) which possesses an excellent electron affinity, feature which has prompted its use as an electron acceptor for the construction of electron donor‐acceptor (D−A) conjugates.^[25]^ Another interesting feature of CPA is its intense optical absorption covering all the way from the UV to the near‐infrared (NIR) region (Figure S3.7a).

However, despite the use of CPA for the construction of many photo‐responsive conjugates,^[26]^ no reports have addressed the origin of the panchromatic absorption presented by this small PAH, a feature particularly striking if compared to anthracene, its structurally simpler analogue, which does not show any significant absorption beyond 400 nm (Figure S3.7b). Moreover, anthracene is moderately fluorescent (i.e., fluorescence quantum yield (Φ_f_) ~0.3)^[27]^ whereas CPA is poorly emissive (i.e., Φ_f_ >0.002).^[28]^ To address this issue, we focused our attention on the CPA frontier molecular orbitals (FMOs), which alert of its non‐alternant character resulting from the presence in its structure of two rings containing an odd number (i.e., 5) of carbon atoms.^[29]^ In terms of valence bond theory, of the three canonical forms of CPA shown in Figure 2a, the acene‐like structure (Figure 2a, left) is not represented in the FMOs, which conversely resemble a diradicaloid structure featuring two cyclopentadienyl radicals in the case of the HOMO (Figure 2b, left) and a 16 electron, antiaromatic species in the case of the LUMO (Figure 2b, right). While CPA has a closed‐shell electronic structure with a negligible diradical character resulting from the small contribution of the cyclopentadienyl radical forms (Figure S10.2), its peculiar optical features (i.e., NIR absorption and poor emission) clearly suggest a not negligible contribution of the non‐acenic forms in the HOMO (Figure 2a, center) and LUMO (Figure 2a, right).

More specifically, the low energy absorption band in CPA might results from i) the presence of non‐bonding atoms in both the HOMO and the LUMO, and/or ii) the poor overlap between the HOMO and the LUMO which reduces the exchange integral and thus the oscillator strength. The reduced optical gap in CPA makes the HOMO–LUMO excitation to occur at longer wavelengths something which, in turn, increases the non‐radiative deactivation pathway of the excited state resulting in low fluorescence.[^30^, ^31^]

**Figure 3:**
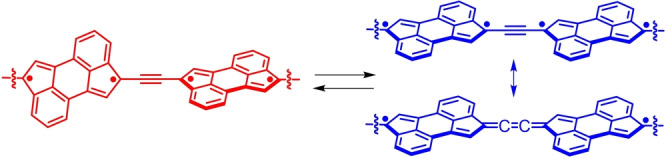
Schematic representation of the proposed conformation‐dependent acetylene‐to‐cumulene transformation in a generic 1D CPA dimer.

Once unravelled the incipient pro‐diradicaloid character of CPA, we decided to investigate its possible use for the realization of an acetylene‐based 1D system in which the (closed‐shell) acetylene to (diradicaloid) cumulene transformation could take place reversibly. In this context, we envisaged that a CPA dimer having an acetylene π‐bridge connecting two CPAs at the carbon atoms of two the five‐member rings sustaining a certain radical character could achieve this goal (Figure 3). We hypothesize that, in such dimer, an out‐of‐plane arrangement of the two CPAs would lead to a more pronounced acetylene‐type character of the central π‐connector due to poor orbital overlap of the two PAHs (Figure 3, left). On the other hand, a more planar conformation of the 1D dimer would allow for a better orbital overlap between the two pro‐diradicaloid CPAs and the central π‐wire, a coupling that should promote a partial “cumulenization” of the central π‐bridge (Figure 3, right).

**Scheme 1:**
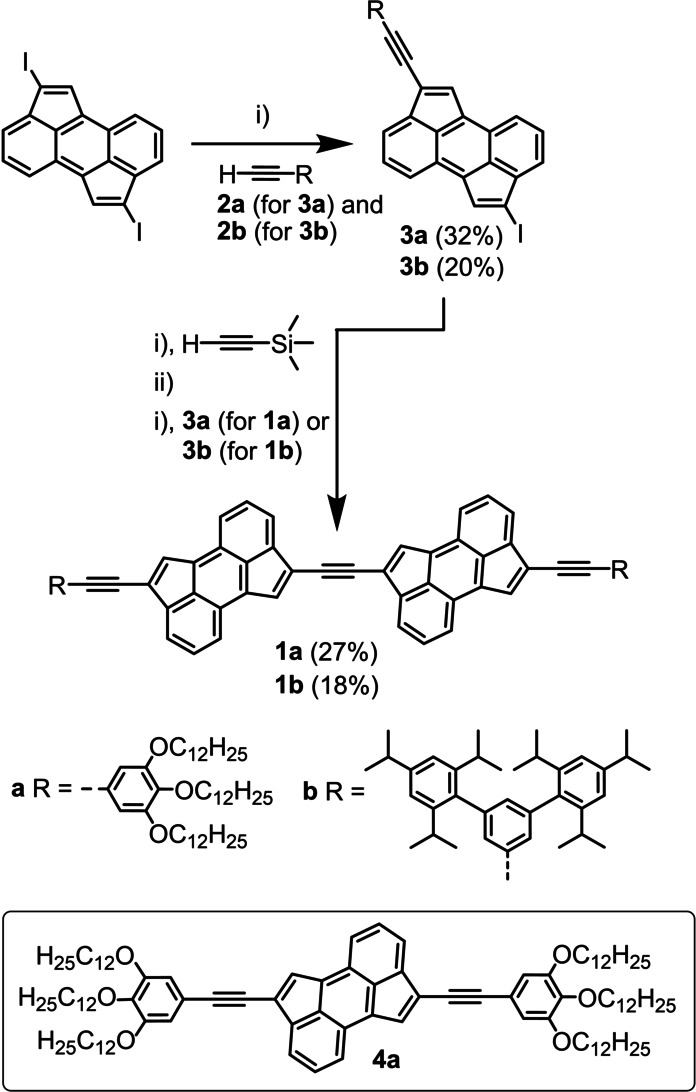
Reaction scheme for the preparation of CPA dimers **1** **a** and **1** **b**. Reaction conditions: i) Pd(PPh_3_)_4_, CuI, THF, NEt_3_, room temperature, overnight; ii) K_2_CO_3_, THF, MeOH, room temperature, 3 h. Ethynyl‐functionalized derivatives **2** **a** and **2** **b** were used as reactants for the preparation of **3** **a** and **3** **b**, respectively. Similarly, the latter were used as reactants for the preparation of CPA dimers **1** **a** and **2** **b**, respectively. The molecular structure of CPA monomer **4** **a** used as reference compound is shown in the frame.

To accomplish this objective, CPA dimers **1** **a** and **1** **b** which differ in the nature of their peripheral groups (i.e., long aliphatic dodecyloxy chains in the case of **1** **a** and bulky 2,4,6‐isopropylphenyl units in the case of **1** **b**) were prepared and studied (Scheme 1). A thorough study on the prepared CPA dimers using spectroscopic analysis, coupled with theoretical calculations, points out to a notable enhancement in the π‐electronic delocalization of the pro‐diradicaloid CPA units through the central acetylene π‐connector upon the dimers’ planarization, which, in turn, increases the cumulenic character of the acetylenic π‐bridge. Notably, this feature is further amplified in **1** **a** under specific conditions: notably, at low temperatures and in methylcyclohexane (MCH) due to an aggregation‐induced planarization process.

## Results and Discussion

The synthesis of CPA dimer **1** **a** started with a Sonogashira coupling reaction between 2,7‐diiodo CPA^[27]^ and ethynyl‐functionalized **2** **a** affording **3** **a** in a 32 % yield (Scheme 1).^[32]^ Next, a one‐pot synthetic procedure involving i) a metal‐catalysed coupling reaction between iodo‐substituted CPA **3** **a** and trimethylsilylacetylene, followed by ii) in situ deprotection of the resulting TMS‐substituted CPA derivative using K_2_CO_3_, and iii) Sonogashira coupling between the ethynyl‐terminated CPA derivative and **3** **a** was employed, giving rise to CPA dimer **1** **a** in an overall 27 % yield.^[33]^ A CPA dimer analogue of **1** **a**, namely **1** **b**, was also prepared using a similar synthetic strategy (Scheme 1). Both CPA dimers **1** **a** and **1** **b** were fully characterized by a wide range of spectroscopic, spectrometric, and microscopic techniques (see Supporting Information).

The UV/Vis absorption spectrum of **1** **a** in CHCl_3_ at 25 °C shows an intense, high‐energy band at 307 nm together with some lower absorbance electronic transitions between 350 and 500 nm. Beside such high‐energy transitions, a broad, low‐energy band appears spanning from 550 to 900 nm with two maxima at 739 and 808 nm (Figure S3.6), an optical transition which theoretical calculations attributed to a HOMO→LUMO transition (see Section 10, Supporting Information).

**Figure 4:**
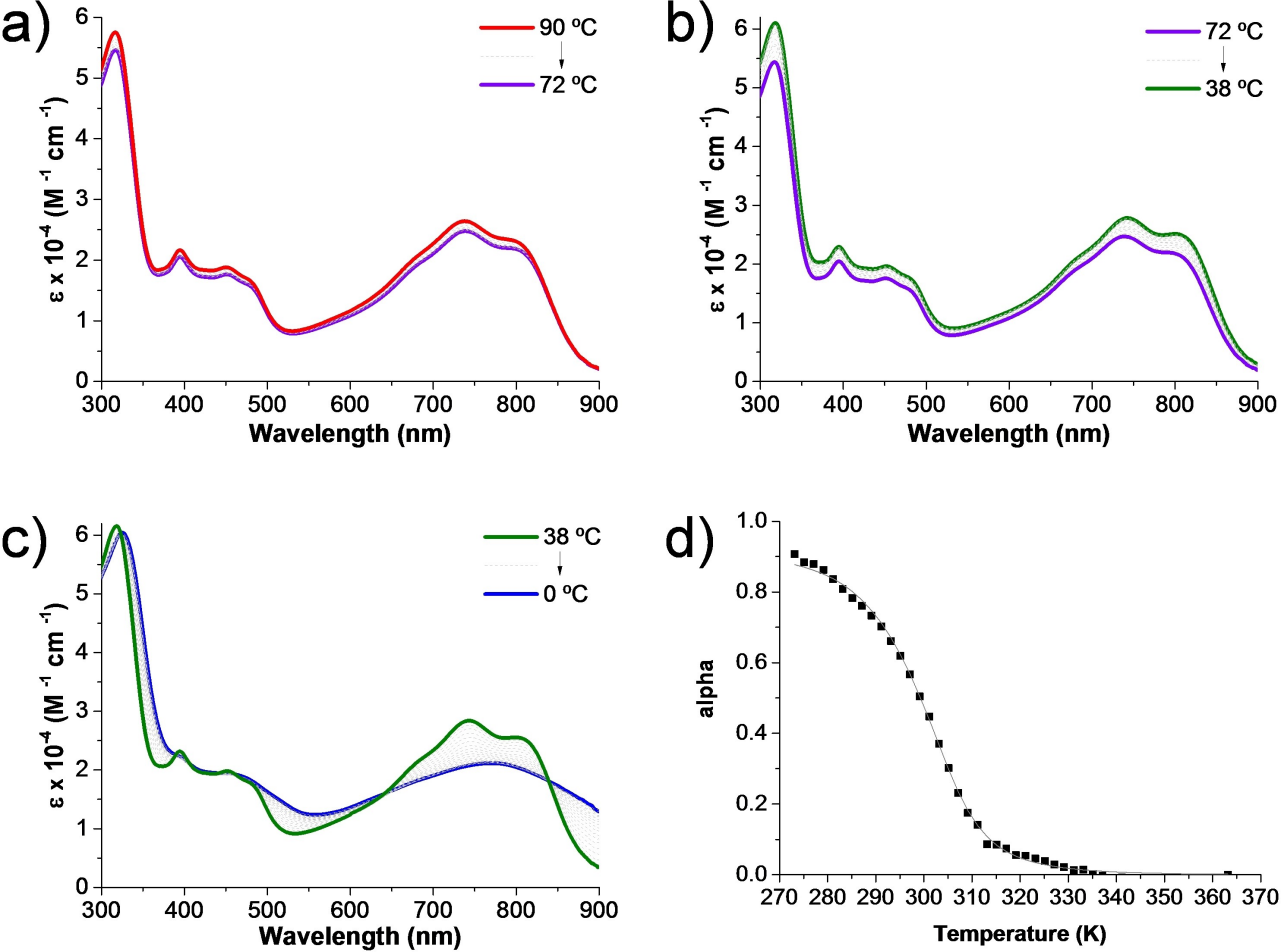
a–c) VT UV/Vis absorption spectra of CPA dimer **1** **a** in MCH ([**1** **a**]=2.6×10^−5^ M) between 90 and 0 °C. Intermediate spectra recorded every 2 °C (dashed grey lines). Cooling rate: 0.2 °C/min. d) Cooling curve data (square dots) of CPA dimer **1** **a** in MCH ([**1** **a**]=2.6×10^−5^ M, temperature range from 90 (i.e., 363 K) to 0 °C (i.e., 273 K)) monitored at 875 nm fitted to a nucleation‐elongation model (solid lines).

Interestingly, changing the solvent from CHCl_3_ to MCH, a less polar medium, while maintaining the temperature to 25 °C, results in the intensity decrease and redshift of the broad, low‐energy bands (Figure S3.6). To gain insight into the origin of such change in the NIR absorption of **1** **a** in MCH, variable‐temperature (VT) UV/Vis experiments at 2.6×10^−5^ M were carried out. Three different spectral “regimes” were observed. Upon decreasing the temperature from 90 °C, a small decrease (i.e., regime A) (Figure 4a) followed by a slight increase (i.e., regime B) (Figure 4b) in the intensity of the CPA dimer absorption is observed throughout the investigated spectral window.^[34]^ For both regimes, a similarity between the shape of the absorption spectra of **1** **a** in MCH at high temperatures (e.g., 90 °C) and the spectrum of the same dimer in CHCl_3_ at 25 °C is evident (Figure S3.6). Interestingly, further lowering the temperature in MCH results in a drastic change in the CPA dimer absorption spectrum (i.e., regime C), in particular, a redshift and intensity reduction of the low‐energy band (Figure 4c). Well‐defined isosbestic points at 324, 388, 642, and 839 nm point out to a clear‐cut transformation between two different species in solution. It is important to highlight that the low temperature‐induced changes observed for **1** **a** in MCH do not take place neither for the CPA monomer **4** **a** in the same solvent (Figure S3.2), nor for the CPA dimer itself in CHCl_3_ (Figure S3.4). This suggests that both the dimeric structure of **1** **a** as well as the nature of the solvent (i.e., MCH) play a crucial role in triggering the observed low temperature‐induced redshift of the NIR bands.

**Figure 5:**
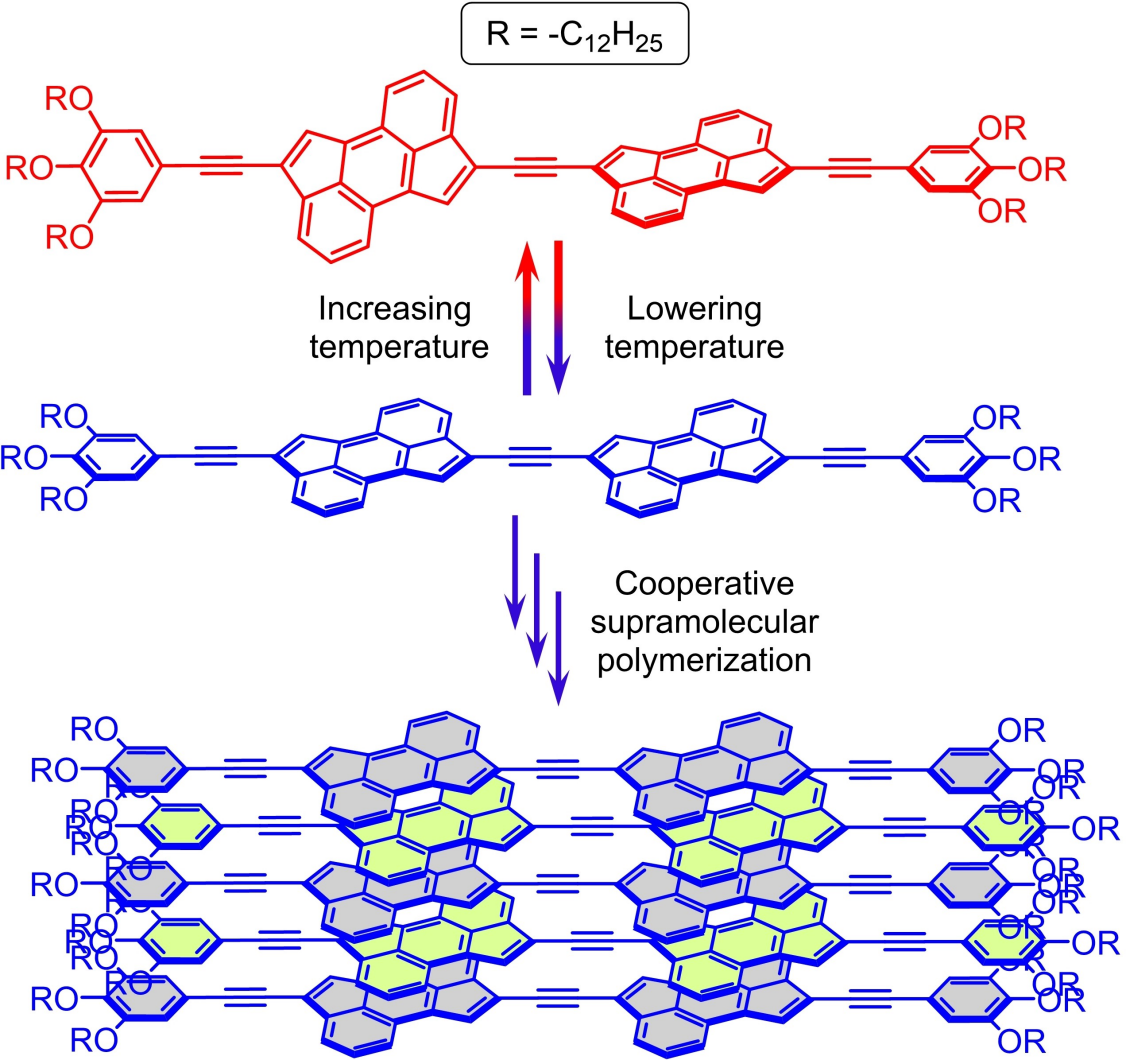
Proposed mechanism for the low temperature‐induced supramolecular polymerization of CPA dimer **1** **a** in MCH. In order to facilitate the visualization of the stacked arrangement of **1** **a** within the supramolecular polymer, the aromatic rings of the stacked CPA dimers have been filled in light grey and light green colour in an alternate fashion.

To unravel the origin of the changes occurring in the absorption spectrum of **1** **a** in MCH in regime C, VT UV/Vis experiments at different concentrations (i.e., from 6.5×10^−6^ to 7.8×10^−5^ M) were carried out (Figure S3.1). While for all the investigated concentrations, regimes A, B, and C were still observed, a significant increase of the upper temperature at which regime C sets in (i.e., the transition temperature between regimes B and C) was seen upon increasing the concentration (e.g., from 22 °C at 6.5×10^−6^ M to 52 °C at 7.8×10^−5^ M) (Figure S3.5). Such temperature‐concentration relationship points out to an intermolecular process taking place for **1** **a** in regime C. Taking this into account, we ascribe the absorption band tailing into the NIR region observed for **1** **a** in MCH in regime C upon lowering the temperature to the formation of supramolecular aggregates. However, considering that no substantial temperature‐ or concentration‐dependent changes of the absorption features of CPA monomer **4** **a** in MCH or CPA dimer **1** **a** in CHCl_3_ occur (see above), we hypothesize that, for **1** **a** in MCH, a decrease in temperature induces a more planar arrangement of the two acetylene‐spaced CPA moieties (see below). Such conformational change, which provides an optimal electronic surface for extended π‐conjugation and intermolecular π–π contacts, is crucial in triggering the formation of the supramolecular aggregates through a combination of CPA π‐stacking as well as van der Waals interactions between the long, peripheral alkoxy chains (Figure 5). To further substantiate the involvement of CPA π‐stacking interactions as one of the key forces in driving the formation of the supramolecular aggregates in **1** **a**, VT UV/Vis control experiments were realized on **1** **b**, a CPA dimer in which the two terminal trialkoxyphenyl moieties of **1** **a** have been formally replaced by bulky 2,4,6‐isopropylphenyl units. For CPA dimer **1** **b**, no sign of aggregation (i.e., appearance of NIR bands) was observed in MCH upon lowering the temperature, even at “high” concentrations (Figure S3.3).

**Figure 6:**
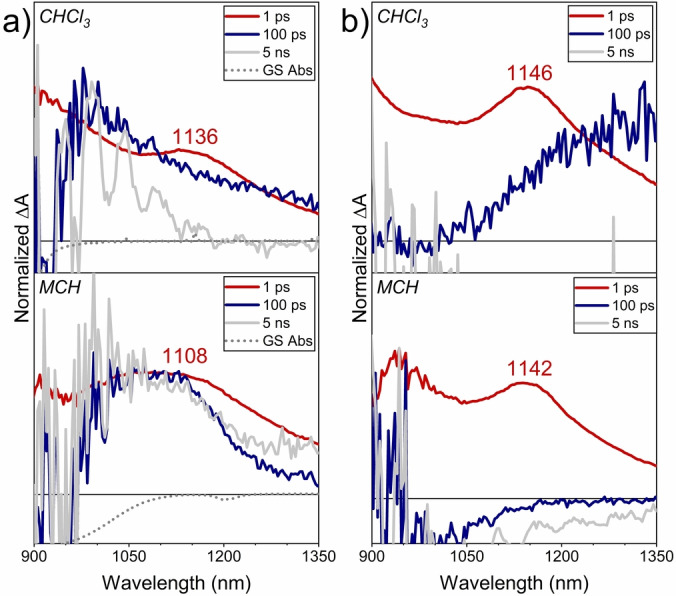
Normalized *fs*‐TEA spectra of CPA dimers a) **1** **a** and b) **1** **b** in CHCl_3_ (top) and MCH (bottom) at room temperature at selected delay times: 1 ps (red, lines), 100 ps (blue lines), and 5 ns (grey lines). Ground state absorption of **1** **a** in CHCl_3_ and MCH are also showed for reference (grey, dotted lines).

The optical characterization of CPA dimers **1** **a** and **1** **b** was complemented with femtosecond transient electronic absorption spectroscopy (*fs*‐TEAS) studies. Figure 6a shows normalized transient absorption spectra of **1** **a** in CHCl_3_ and MCH at room temperature in the NIR region at selected delay times. Photoexcitation of **1** **a** in both solvents generated a similar prompt (i.e., subpicosecond timescale) excited state absorption (ESA) profile at ca. 1100 nm (see Section 3 in the Supporting Information). However, the time evolution of this ESA band strongly depends on the solvent. While no relevant optical features were observed at 100 ps in CHCl_3_, in MCH a portion of this ESA band is still visible at 5 ns (i.e., the time window limit of our setup). The *fs*‐TEAS of **1** **a** in CHCl_3_ is consistent with the fast formation of a singlet excited state (ESA at 1156 nm) which decays in the picosecond timescale (7.8 ps) in line to what observed for the photodynamics of a CPA monomer analogous to **4** **a**.^[28]^ In contrast to the fast, non‐radiative decay channel governing the photodynamic of the molecularly dispersed species in CHCl_3_, long‐living ESA bands are observed for **1** **a** in MCH at long delay times (Table S1), which suggests exciton delocalization due to aggregation, as previously observed in supramolecular aggregates.[^35^, ^36^] Moreover, control experiments on “bulky” CPA dimer **1** **b** further substantiate this assumption. For this CPA dimer which does not form supramolecular aggregates, the same behaviour is observed in CHCl_3_ and MCH, with an ESA lifetime of less than 10 ps (Figures 6b and S3.8b).

Once established the formation of supramolecular aggregates as the responsible for the observed spectral changes of **1** **a** in MCH, we turned our attention at disclosing the mechanism underlining the supramolecular polymerization process. With this aim, we analysed the changes in the absorbance intensity (λ=875 nm) as a function of the temperature (i.e., from 90 to 0 °C) and at different concentrations. The plots of the fraction of aggregated **1** **a** (α_agg_) as a function of the temperature showed non‐sigmoidal curves (Figures 4d and S4.1, suggesting a nucleation–elongation polymerization mechanism which implies the initial, slow formation of stable, yet energetically unfavourable, nuclei of **1** **a** (i.e., the nucleation phase) followed by the rapid, energetically favourable addition of monomers of **1** **a** to such nuclei (i.e., the elongation phase) leading to thermodynamically stable supramolecular polymers. The different melting curves obtained were successfully fitted by the cooperative model developed by ten Eikelder, Markvoort, Meijer and co‐workers,[^37^, ^38^, ^39^] which allows to extract some thermodynamic parameters of the cooperative supramolecular polymerization process such as *σ* (i.e., 0.067), a value which provides insights into whether a given system polymerize following a cooperative (i.e., *σ* <1) or non‐cooperative (i.e., *σ*=1) growth mechanism (Table 1).

**Table 1:** Thermodynamic parameters of the cooperative supramolecular polymerization of CPA dimer **1** **a** in MCH obtained from temperature‐dependent UV/Vis absorption experiments at different concentrations using the ten Eikelder–Markvoort–Meijer model. Elongation temperature (*T*_e_), elongation enthalpy (Δ*H*_e_) and entropy (Δ*S_e_*), nucleation enthalpy (Δ*H*_n_), nucleation (*K*_n_) and elongation (*K*_e_) constants, degree of cooperativity (σ).

ΔH_n_ (kJ mol^−1^)	K_n_ (M^−1^)	T_e_ (K)	ΔH_e_ (kJ mol^−1^)	ΔS_e_ (J mol^−1^ K^−1^)	K_e_ (M^−1^)	σ
−6.9±0.2	2183.7	306.8±0.7	−76.4±1.2	−0.163±0.004	32367.9	0.067

Supported by the notion that the low temperature‐induced aggregation capability of **1** **a** in MCH is strongly dependent on the CPA dimer concentration, we focused our attention on the possibility of promoting the aggregation of **1** **a** in CHCl_3_, a solvent in which the CPA dimer did not show any sign of aggregates formation in the UV/Vis absorption experiments (see above). ^1^H NMR experiments of **1** **a** in CDCl_3_ ([**1** **a**]=5×10^−3^ M; i.e., a 2‐fold concentration increase compared to the one used for the VT UV/Vis absorption experiments in CHCl_3_) showed sharp proton peaks at 25 °C (Figure S2.9). However, upon lowering the temperature, a broadening of the dimer dodecyloxy and, particularly, the CPA proton peaks was observed, a process which reached its maximum close to −45 °C (Figure S6.1). Taking into account that the low temperature‐promoted ^1^H NMR peaks broadening cannot be ascribed to the precipitation of **1** **a** nor to the freezing of the solvent (i.e., the CDCl_3_ solution at −45 °C was perfectly transparent and fluid),^[40]^ a possible explanation of the observed phenomenon is the formation of supramolecular polymers, as postulated for the VT UV/Vis absorption experiments of **1** **a** in MCH (see above). Such self‐assembly process could be responsible, in the ^1^H NMR spectrum, for the significant broadening of the CPA protons due to the PAH stacked arrangement, while exerting an attenuated effect on the protons of the peripheral alkoxy units. Increasing the temperature from −55 °C back to 25 °C caused a gradual re‐sharpening of the ^1^H NMR proton peaks in **1** **a**. However, the final spectrum was significantly broader that the one recorded before the cooling/heating cycle (Figure S6.2a,b). For the spectrum to fully recover its original sharpness, the previously cooled/heated CHCl_3_ solution had to be left at room temperature for a few days (Figure S6.2c). The observed “hysteresis” points out to the presence of some kinetic effects in the assembly/disassembly of the supramolecular aggregates, a phenomenon previously reported for other supramolecular ensembles.^[41]^ Interestingly, such hysteresis was not observed upon an abrupt freezing of a CDCl_3_ solution of **1** **a** down to −198 °C using liquid nitrogen followed by its rapid heating to 25 °C (Figure S6.2d). This finding suggests that a gradual decrease of the temperature is needed for CPA dimer **1** **a** to properly self‐organize into supramolecular aggregates.

To further support the hypothesis of the low temperature‐induced formation of supramolecular aggregates in a “concentrated” CDCl_3_ solution of **1** **a**, VT ^1^H NMR experiments were carried out on “bulky” CPA dimer **1** **b** in the same solvent and at the same concentration used for **1** **a**. For **1** **b**, no sign of proton peaks broadening was observed upon lowering the temperature (Figure S6.3).

The propensity of **1** **a** to form aggregates was also investigated by atomic force microscopy depositing MCH solutions of **1** **a** on highly ordered pyrolytic graphite which revealed the formation of μm‐long fibers and μm‐wide islands (see Section 5 in the Supporting Information).

Since it is reasonable to assume that the low temperature‐induced aggregation process in **1** **a** is triggered by a planarization of the CPA dimer (see above), Raman spectroscopy was used to obtain information regarding possible changes in the “electronic” nature of the π‐bridge upon aggregation, which could point out to hypothesized acetylene‐to‐cumulene transformation. Firstly, solid‐state Raman experiments were carried out on CPA monomer **4** **a** and CPA dimers **1** **a** and **1** **b** (Figure S8.1). Focusing on the 2200–2100 cm^−1^ region where the stretching vibrations of the C≡C acetylene moiety usually appear, hereafter denoted as ν_(C≡C)_, only one intense Raman band at 2183 cm^−1^ is observed for **4** **a**, whereas two different ν_(C≡C)_ bands are seen for **1** **a** and **1** **b** (Figure S8.2) in two separated wavenumber regions between 2200 and 2100 cm^−1^. On one hand, for both CPA dimers, higher‐energy modes are observed at 2183 cm^−1^ (for **1** **a**) and 2188 cm^−1^ (for **1** **b**), which are associated, as in the case of **4** **a**, to vibrations of the two external acetylene groups (i.e., ν_(C≡C)*external*_). The low intensity of those Raman bands in both CPA dimers suggests that they are out of the region of the main electronic/structural π‐conjugation changes. On the other hand, **1** **a** and **1** **b** show intense Raman bands appearing in the 2160–2060 cm^−1^ interval, which are attributed to the internal acetylene spacer in both CPA dimers (i.e., ν_(C≡C)*central*_). More specifically, for **1** **a** the more intense Raman peaks are detected at 2148 and 2140 cm^−1^, whereas those of “bulky” CPA dimer **1** **b** appear at 2158 and 2148 cm^−1^, both upshifted by ~8–11 cm^−1^ with respect to those in **1** **a**.

**Figure 7:**
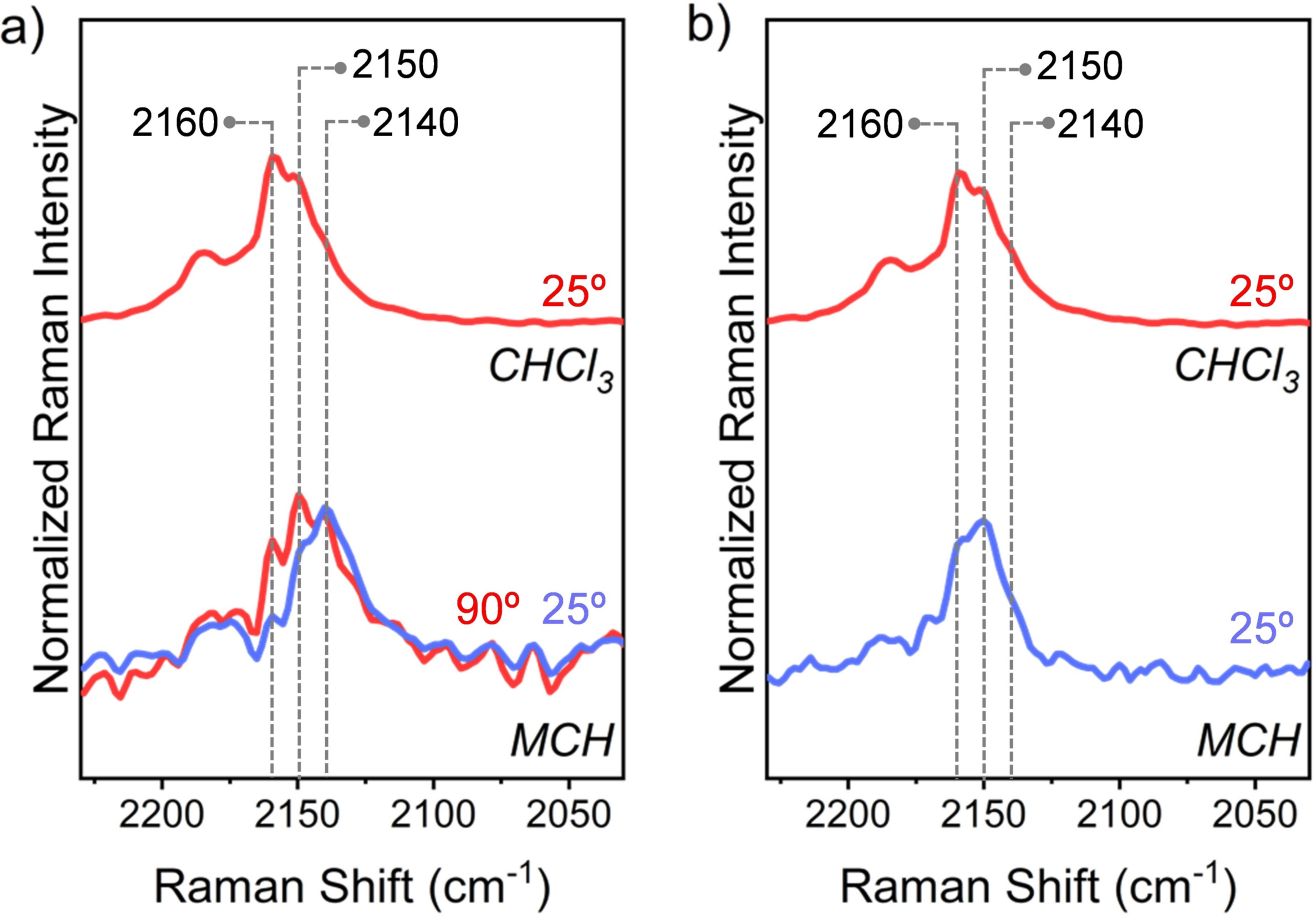
FT‐Raman spectra of CPA dimers a) **1** **a** and b) **1** **b** at 1×10^−3^ M in MCH (bottom) at 25 °C (blue lines) and 90 °C (red line), and CHCl_3_ (top) at 25 °C (red lines). To facilitate the spectral comparison, three vertical dashed lines have been drawn at three selected frequencies.

With this information in hand, “concentrated” solutions (i.e., 1×10^−3^ M) of **1** **a** and **1** **b** in MCH were prepared and their FT‐Raman spectra at different temperatures recorded (Figures 7 (bottom spectra), and S8.3 and S8.4).^[42]^ The Raman spectrum of **1** **a** in MCH at 90 °C displays three main peaks in the 2160–2060 cm^−1^ ν_(C≡C)*central*_ frequency window, namely at 2160, 2150, and 2140 cm^−1^ (Figure 7a, bottom). Lowering the temperature to 25 °C afforded a Raman spectrum closely akin to the one obtained at 90 °C in terms of peaks position, but significantly different in terms of the relative intensities of the three main peaks, being the low‐energy one at 2140 cm^−1^ the most intense one.

To elucidate the origin of these three vibrational Raman transitions, a control Raman experiment on **1** **a** in CHCl_3_ at 25 °C was carried out. The resulting Raman spectrum showed two intense peaks at 2159 and 2150 cm^−1^, with no sign of the peak at 2140 cm^−1^ (Figure 7a, top). Taking into account that: i) the latter Raman experiment on **1** **a** was carried out in similar experimental conditions (i.e., CHCl_3_, 25 °C, and [**1** **a**]=1×10^−3^ M) to the one used for the ^1^H NMR studies (i.e., CDCl_3_, 25 °C, and [**1** **a**]=5×10^−3^ M); and ii) the latter ^1^H NMR investigation did not show any sign of supramolecular aggregate formation (see above), one could speculate that the higher‐wavenumber Raman peaks of **1** **a** in CHCl_3_ observed at 2159 and 2150 cm^−1^ are predominantly attributable to its molecularly dispersed species. On the other hand, considering that **1** **a** is able to aggregate in “diluted” (i.e., 10^−5–^10^−6^ M) MCH solutions at 25 °C, as demonstrated in VT UV/Vis absorption experiments (see above), it is reasonable to assume that such self‐assembly would also take place for more concentrated MCH solutions as those used in the Raman experiments (i.e., 10^−3^ M) at 25 °C.^[43]^ Considering all such evidences, it is a logical assignment that the lower‐energy Raman peak at 2140 cm^−1^ results from the ν_(C≡C)*central*_ of CPA dimer **1** **a** in its self‐assembled state.

To further substantiate this hypothesis, a VT Raman control experiment in MCH was performed on **1** **b** which did not show aggregation in VT UV/Vis absorption experiments in MCH (see above). The Raman spectrum of **1** **b** in MCH at 25 °C was different from that of **1** **a** recorded in the same experimental conditions. In the former case, two main bands at 2159 and 2150 cm^−1^ are seen (Figure 7b, bottom). As in the case of **1** **a**, the two higher‐wavenumber peaks in **1** **b** are most probably related to the molecularly dispersed CPA dimer, as also corroborated by the Raman spectrum of **1** **b** in CHCl_3_ at 25 °C which also shows two peaks at 2159 and 2150 cm^−1^ (Figure 7b, top). However, in **1** **b**, differently than in the case of **1** **a**, no Raman peak at 2140 cm^−1^ is observed at 25 °C, neither in MCH nor in CHCl_3_, further validating the hypothesis that such low‐energy Raman band in **1** **a** arises from the aggregated species.

**Figure 8:**
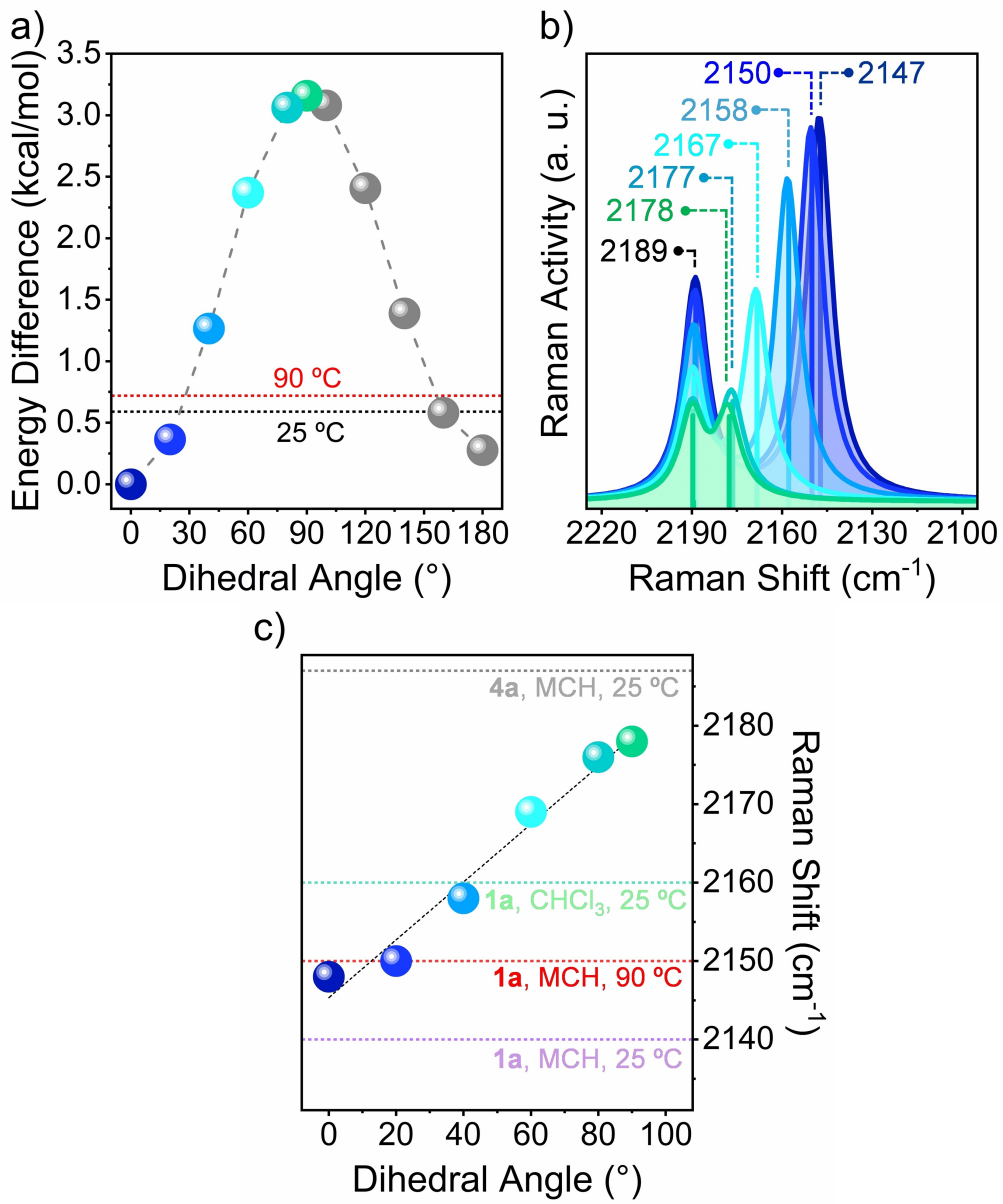
a) Calculated energy difference (kcal/mol) in the gas phase between the optimized geometry of CPA dimer **1** **a** as a function of the dihedral angle between the two CPA units (thereafter referred as Θ) (0°≤Θ≤180°). The two horizontal lines indicates the thermal energy (ET) of **1** **a** at 25 °C (black dashed line) and 90 °C (red dashed line); b) Variation of the Raman ν_(C≡C)*central*_ of **1** **a** as a function of Θ (0°≤Θ≤90°) (*N.B*.: ν_(C≡C)*external*_ remains unchanged during the Θ scan since the dihedral angle between the peripheral phenyl ring and its proximal CPA was kept fixed at 0°). The spectra colour code corresponds to that used in a); c) Linear fitting of the calculated Raman ν_(C≡C)*central*_ (circles colour code corresponds to that used in a) as a function of the Θ (R^2^=0.973). In the same Figure, the most intense experimental ν_(C≡C)*central*_ Raman peak of CPA monomer **4** **a** in MCH at 25 °C (grey horizontal dashed line), and CPA dimer **1** **a** in CHCl_3_ at 25 °C (green horizontal dashed line) and in MCH at 25 °C (red horizontal dashed line) and 90 °C (violet horizontal dashed line) have been included. Calculations were performed at the B3LYP/6‐31G** level of theory and theoretical Raman spectra were scaled down uniformly by a factor of 0.96.

Considering that the Raman features of **1** **a**, either in its molecularly dispersed or aggregated state, are strongly dependent on its electronic and molecular structure, we employed quantum chemical calculations to underpin such structure–property relationship and gather information regarding possible changes in the nature of the π‐connector in this CPA dimer. With this aim, the rotational energy barrier of **1** **a** as a function of the dihedral angle between its two CPA subunits (thereafter referred as Θ) was computed (Figure 8a). On one hand, theoretical studies in gas phase showed that the two planar conformations (i.e., Θ=0° or 180°) are the most energetically stable ones with a minor energy difference between them, being the *anti*‐coplanar arrangement (i.e., Θ=0°) the energy minimum (Figure S1.2). On the other hand, the most unstable conformations were those having the two CPAs arranged in an orthogonal fashion (i.e., Θ=90° or 270°). The latter two conformers are ca. 3 kcal/mol higher in energy than the coplanar ones (Figure 8a). Considering that the thermal energy (ET) of a system can be estimated from the formula ET=RT, at 298 K, ET would amount to 0.59 kcal/mol. This implies that, at this temperature, a significantly large population fraction of **1** **a** conformers display a Θ between 0° and 20° (Figure 8a). Increasing the temperature to 90 °C (ET=0.72 kcal/mol) results in a shift of the conformer population towards higher values of Θ (i.e., Θ ~30°). As expected, a similar energy profile is seen for “bulky” CPA dimer **1** **b** (Figure S9.2a).

Next, the theoretical vibrational Raman spectra of **1** **a** (Figures 8b and S8.7, and Table S5) and **1** **b** (Figure S9.2c) as a function of Θ were simulated. These studies show, in both cases, strong active Raman bands between 2100 and 2200 cm^−1^, corresponding to the stretching modes of the two chemically different acetylene groups (i.e., ν_(C≡C)*central*_ and ν_(C≡C)*external*)_. Interestingly, in both CPA dimers, a large (i.e., ~30 cm^−1^) and linear downfield shift of the calculated ν_(C≡C)*central*_ Raman peak is observed upon varying Θ from 90° to 0° (Figures 8c and S9.2c), in a process in which the nature of the acetylene π‐connector, that ultimately determines the Raman ν_(C≡C)*central*_ mode, is strongly influenced by the degree of communication between the two CPA units. From the electronic point of view, the wavenumber downshift of the calculated ν_(C≡C)*central*_ Raman band of **1** **a** and **1** **b** for Θ approaching 0° could be accounted for an increased degree of “cumulenization” of the inner acetylene bridge moving towards a close‐to‐planar conformation of the CPA dimers.

**Figure 9:**
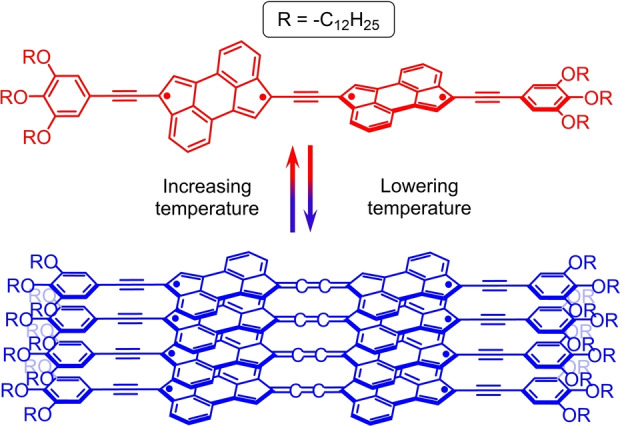
Reversible, temperature‐induced acetylene‐to‐cumulene transformation in CPA dimer **1** **a**.

In light of the Θ‐dependent simulated Raman analysis of **1** **a** and **1** **b**, the presence in **1** **a** of an intense Raman peak at 2140 cm^−1^ in MCH at 25 °C suggests a more planar conformation adopted by this dimer with respect to its “bulky” analogue **1** **b** due to the possibility of the former derivative to self‐aggregate. In turn, such aggregation‐induced planarization of the two prodiradicaloid CPAs promotes a higher π‐electron delocalization through the central acetylene bridge thus increasing its cumulenic character (Figure 9).

Theoretical calculations were also performed to determine the diradical character (y_0_) of CPA dimer **1** **a** (i.e., y_0_=0 for closed‐shell systems and y_0_=1 for open‐shell diradical species), a feature somehow connected to the cumulenic character of the conjugate. For **1** **a**, a y_0_ value of 0.08 was calculated suggesting that **1** **a** presents a closed‐shell electronic configuration with an incipient diradical character.^[44]^

In order to substantiate such hypothesis and test the generality of the proposed acetylene‐to‐cumulene transformation, the rotational energy barrier (Figure S9.2a) and simulated Raman spectra (Figure S9.2d‐f) of three additional acetylene‐bridged dimers as a function of Θ were calculated (see molecular structures in Figure S9.1). Interestingly, for all those three dimers, including a positional isomer of **1** **a** in which two CPAs are connected at their six‐member rings by an acetylene bridge (i.e., **1a_6,6_** in Figure S9.1), a reduced downfield shift of the calculated ν_(C≡C)*central*_ Raman peak for Θ approaching 0° was observed (see Section 9 in the Supporting Information). Such result could be rationalized attending at the number of Clar's sextets of these five dimers in their acetylenic and cumulenic electronic configuration. In this context, only **1** **a** and **1** **b**, which are the dimers which present the highest downfield shift of the calculated ν_(C≡C)*central*_ Raman peak, experience a gaining (i.e., from two to four) in the number of Clar's sextets going from the acetylenic to the cumulenic electronic configuration, whereas for the other dimers this number remains the same or decreases (Figure S9.1).

## Conclusions

Herein, we report a novel strategy aimed at enhancing the cumulenic character of some 1D acetylene‐based conjugates which consists in bridging two pro‐diradicaloid species, namely CPAs, at their pro‐radical positions by a triple bond. To achieve this goal, CPA dimers **1** **a** and **1** **b** which differ in the nature of their peripheral groups (i.e., long aliphatic dodecyloxy chains in the case of **1** **a** and bulky 2,4,6‐isopropylphenyl units in the case of **1** **b**) were prepared and investigated using a wide range of spectroscopic techniques. In this context, low temperature UV/Vis absorption and ^1^H‐RMN studies showed that only **1** **a** is able to form supramolecular aggregates as a result of the planarization of its two CPA moieties in MCH (through a cooperative polymerization process) and in CHCl_3_. Moreover, Raman experiments on **1** **a** in MCH showed a marked downfield shift of the main ν_(C≡C)*central*_ peaks upon lowering the temperature, something not observed for “bulky” CPA dimer **1** **b**. Interestingly, Θ‐dependent simulated Raman analysis on both CPA dimers predicted a downfield shift of the ν_(C≡C)*central*_ Raman peaks moving towards a more planar dimers’ conformation. A rationale behind such result points out to a higher π‐electronic delocalization of the pro‐diradicaloid CPA units through the central acetylene spacer upon the dimers’ planarization which, in turn, increases the cumulenic character of the π‐bridge, a feature further enhanced in the case of **1** **a** at low temperature and in MCH due to the aggregation‐induced planarization taking place for this CPA dimer. We reckon that covalently linking (pro‐)diradicaloid species at their (pro‐)radical positions by an acetylene/polyyne bridge represents an interesting approach towards the realization of 1D systems which cumulenic character of the π‐connector could be enhanced in a reversible fashion and under mild conditions. This may open the way towards the realization and study of stimuli‐responsive, electronically switchable 1D π‐conjugated systems.

## Conflict of Interests

The authors declare no conflict of interest.

1

## Supporting information

As a service to our authors and readers, this journal provides supporting information supplied by the authors. Such materials are peer reviewed and may be re‐organized for online delivery, but are not copy‐edited or typeset. Technical support issues arising from supporting information (other than missing files) should be addressed to the authors.

Supporting Information

## Data Availability

The data that support the findings of this study are available in the supplementary material of this article.
