# Supplementary material for: Enhancing the Cumulene Character of One‐Dimensional Acetylene‐Based Systems by Stimuli‐Induced Planarization of Their Two Pro‐diradicaloid Cyclopenta[h,i]aceanthrylene Units
Source: Angew Chem Int Ed Engl. 2024 Nov 21;64(7):e202419832. doi: 10.1002/anie.202419832 (PMC11811688; doi:10.1002/anie.202419832)
Supplement: Supplementary file 1 — Supporting Information [file ANIE-64-e202419832-s001.pdf]

Angewandte Chemie
Eine Zeitschrift der Gesellschaft Deutscher Chemiker

Supporting Information

Enhancing the Cumulene Character of One-Dimensional Acetylene-Based Systems by Stimuli-Induced Planarization of Their Two Pro-diradicaloid Cyclopenta[*h,i*]aceanthrylene Units

Á. C. Fernández, S. M. Rivero, S. Chaurasia, T. Torres, J. Casado*, G. Bottari**

Supporting Information for

Enhancing the Cumulene Character of One-Dimensional Acetylene-Based Systems by Stimuli-Induced Planarization of Their Two Pro-diradicaloid Cyclopenta[*h,i*]aceanthrylene Units

Álvaro Corrochano Fernández,[+,a,b] Samara Medina Rivero,[+,d] Sumit Chaurasia,[a] Tomás Torres,*[,a,b,c] Juan Casado,*[,d] Giovanni Bottari*[,a,b,c]

[a] Departamento de Química Orgánica, Universidad Autónoma de Madrid, Campus de Cantoblanco, 28049 Madrid (Spain)

[b] IMDEA-Nanociencia, Campus de Cantoblanco, 28049 Madrid (Spain)

[c] Institute for Advanced Research in Chemical Sciences (IAdChem), Universidad Autónoma de Madrid, 28049, Madrid (Spain)

[d] Department of Physical Chemistry, University of Málaga, Andalucia-Tech, Campus de Teatinos s/n, 29071 Málaga (Spain)

E-mail: giovanni.bottari@uam.es; casado@uma.es; tomas.torres@uam.es

[+] These authors contributed equally to this work.

__**Table of Content**__

Page

1. **Materials and methods……………………….……………………………… S-3**
2. **Synthesis and characterization of CPA dimers 1a and 1b …………….…….. S-6**
3. **Steady state and time resolved UV-vis absorption studies on CPA dimers 1a and 1b and reference compounds, electrochemistry of CPA dimer 1a, and spectroelectrochemistry (UV-vis-NIR and IR electronic absorption) of CPA dimer 1a and CPA monomer 4a ………………………………………………… S-23**
4. **Nucleation-elongation model for cooperative supramolecular polymerizations………………………………………………………........ S-30**
5. **AFM studies on CPA dimers 1a and 1b…………………………...…..…. S-32**
6. **VT ^{1}H-NMR analysis on CPA dimers 1a and 1b……………………………… S-34**
7. **Molecular modelling of CPA dimer 1a and possible organization arrangement on HOPG………………………………………………………… S-38**
8. **Experimental and calculated Raman spectra of CPA dimers 1a and 1b, and CPA monomer 4a ……………………………………………………….……. S-39**
9. **Calculated rotational energy barrier and Raman spectra of 1a and reference PAH dimers ……………………………………………………………………… S-46**
10. **Theoretical calculations on CPA dimer 1a and CPA monomer 4a …………. S-49**
11. **Optimized coordinates……………………..………………………………… S-51**
12. **Supporting Information References……………..…………………….…. S-100**

Abbreviations:

AFM = atomic force microscopy; APCI = atmospheric pressure chemical ionization; CPA = cyclopenta[*hi*]aceanthrylene; DCTB = *trans*-2-[3-(4-*tert*-butylphenyl)-2-methyl-2-propenylidene]malononitrile; FMO = frontier molecular orbital; FT-IR (ATR) = Fourier-transform infrared (attenuated total reflection); HOPG = highly ordered pyrolytic graphite; MALDI-TOF = matrix-assisted laser desorption/ionization time-of-flight; MCH = methylcyclohexane; MS = mass spectrometry; NEt_3 = triethylamine; R_f = retention factor; r.p.m. = revolutions per minute; r.t. = room temperature; THF = tetrahydrofurane; TLC = thin layer chromatography; VT = variable temperature.

1. Materials and methods

Chemicals and solvents were purchased from commercial suppliers (Aldrich, Acros Organics, Alfa Aesar, TCI, and Fluorochem) and used without further purification. All dry solvents were freshly distilled under argon over an appropriate drying agent before use. Column chromatography was carried out on silica gel Merck-60 (230-400 mesh, 0.040-0.063 nm). Analytical TLC was performed on aluminum sheets precoated with silica gel 60 F-254 (0.2 nm thick) from Merck. In specific cases, size exclusion chromatography was performed using Bio-Beads S-X1 (200- 400 mesh, Bio-Rad). ^{1}H- and ^{13}C-NMR spectra were recorded with a Bruker DPX 400 MHz instrument. Chemical shifts values (δ) are referred to tetramethylsilane. VT-^{1}H-NMR spectra were recorded in a BRUKER AC-500 (500 MHz) instrument.

IR spectra were recorded on a Bruker ALPHA Platinum-ATR system.

UV-vis spectra were recorded with a UV-Vis JASCO V660 spectrophotometer using spectroscopic grade solvents and 10 × 10 mm quartz cuvette.

MALDI-TOF MS spectra were obtained in a Bruker ULTRAFLEX III (MALDI-TOF/TOF) spectrometer. APCI-MS spectra were obtained from a Bruker MAXIS II spectrometer.

AFM measurements were carried out in a commercial AFM system (Ntegra Prima, NT-MDT) in semicontact (dynamic) mode using scanning by sample configuration in ambient conditions. Rectangular silicon cantilevers HA_NC (NT-MDT) were used with a tip radius of 10 nm. Their nominal spring constant is 3.5 N/m and its resonance frequency is around 140 kHz. AFM samples were prepared by either drop-casting or spin-coating a solution of the sample dissolved in MCH at different concentrations onto HOPG substrates.

Vibrational Raman Spectroscopy. FT–Raman spectra of solid samples at room temperature (298K) were measured using the RAMII FT-Raman module of a VERTEX 70 FT-IR spectrometer. A continuous–wave Nd–YAG laser working at 1064 nm was employed for excitation, at a laser power in the sample not exceeding 10 mW. Raman scattering radiation was collected in a back–scattering configuration with a standard spectral resolution of 4 cm^{-1}. 10000 scans were averaged for each spectrum.

VT Raman spectra were recorded in MCH (Sigma-Aldrich; anhydrous, ≥99%, CAS number: 108-87-2) using a JASCO Model RFT-6000 FT-Raman accessory in conjunction with the JASCO Model FT/IR-6300 spectrometer. RFT-6000 FT-Raman accessory is equipped with a 1064 nm YAG laser and a InGaAs detector. Each Raman spectrum is an average of 10000 scans collected in scattering mode with a laser power in the sample lower than 10 mW. The ability to

co-add successive sample scans for the Fourier Transform technique compensates for the loss of sensitivity inherent to the weak Raman signals.

Quantum Chemical Calculations. DFT calculations performed with the GAUSSIAN16 suite of programs.[1] Molecular geometry optimizations and theoretical vibrational Raman spectra were performed with the B3LYP functional and the 6-311G** standard basis set.[2,3] Energy optimizations were performed by allowing all geometric parameters to vary independently. The optimum energy structures were found to be a true minimum in the ground state potential energy surface. On the resulting ground-state optimized geometries, harmonic vibrational frequencies and Raman intensities were calculated. We performed an often-employed adjustment of the calculated harmonic vibrational frequencies where all are uniformly scaled down by a constant factor of 0.96.[4] The theoretical Raman spectra were obtained by convoluting the scaled frequencies with Lorentzian functions. Vertical transition energies were computed with the time-dependent version of DFT (TDDFT) formalism[5,6] for which up to the fifty low-lying energy states were considered.

The diradical index (y_0) of CPA dimer **1a** was calculated using the restricted active space spin-flip (RAS-SF) method[7,8] with the Q-Chem program.[9] For that, RAS-SF ab initio calculations with 4 electrons in 4 orbitals in the active space and with the 6-31G** basis set were carried out in the previously calculated DFT optimized geometry at the (U)B3LYP/6-31G** level of theory. y_0 was quantified using the Doehnert-Koutecký definition:[10]

$$y_0 = N_{LUNO}$$

where N_{LUNO} is the lowest unoccupied natural orbital occupation (LUNO) number.

Femtosecond Transient Electronic Absorption Spectroscopy. Pump and probe beams were generated using a Ti:sapphire regenerative amplifier (Spitfire Ace, Spectra-Physics) providing 800 nm pulses (120 fs full-width at half-maximum-FWHM, ≤ 1 kHz repetition rate, average power of 5W). Spitfire Ace is operated with a Mai Tai Ti:Sapphire seed laser (tunable range: 690-1040 nm, 100 fs FWHM, 80 MHz, 3W, Spectra-Physics) and a Nd:YLF Empower 45 Q-switched pump laser (527 nm, 10 ns FWHM, 1 KHz, 15W, Spectra-Physics). Tunable narrowband pump pulses at 730 nm were generated in a TOPAS Prime optical parametric amplifier (output tunable range: 290-1600 nm, Spectra-Physics) with an output power of 0.5

mW. Probe pulses spanning the range 800-1600 nm were generated by focusing a portion of the 800 nm beam through a sapphire crystal. Pump-probe delay was controlled using a direct-drive high-speed optical delay line with a standard 8 ns time window. Detection was carried out using a Helios automated femtosecond transient absorption spectrometer equipped with InGaAs (NIR: 800−1600 nm, spectral resolution: 3.5 nm) detector. The pump and probe polarizations were set to the magic angle. Each spectrum corresponds to an average of at least 4 scans performed over 2 aliquots of solution. Femtosecond transient absorption spectra of CPA dimer **1a** and “bulky” CPA dimer **1b** were recorded in fresh degassed $CHCl_3$ and MCH at *ca.* 10^{-5} M.

2,7-diiodoCPA,[11] CPA monomer **4a**,[12] and ethynyl-based derivatives **2a**[13] and **2b**[14,15] were prepared according to previously reported synthetic procedures showing identical spectroscopic properties to those reported therein (Figure S1.1).

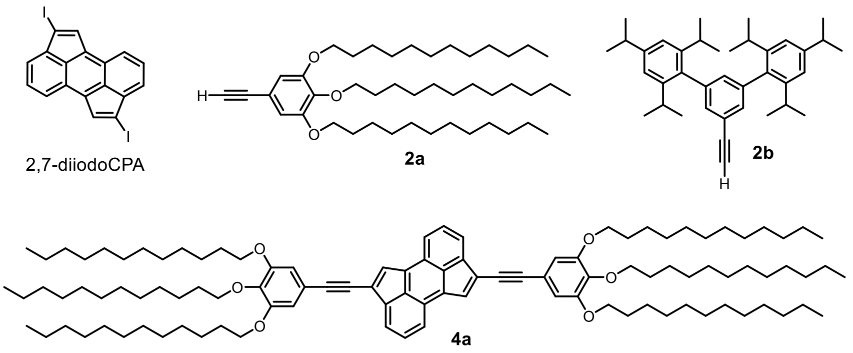

Figure S1.1. Molecular structure of derivatives 2,7-diiodoCPA, CPA monomer **4a**, and acetylene-based derivatives **2a** and **2b**.

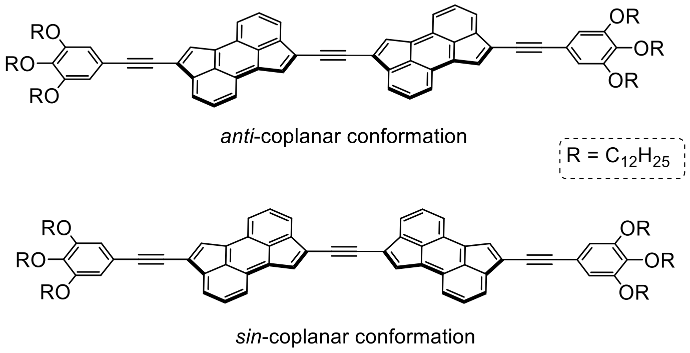

Figure S1.2. *Anti-* (top) and *sin*-coplanar (bottom) conformation of CPA dimer **1a**.

2. Synthesis and characterization of CPA dimers 1a and 1b

*Synthesis and characterization of CPA derivative **3a***

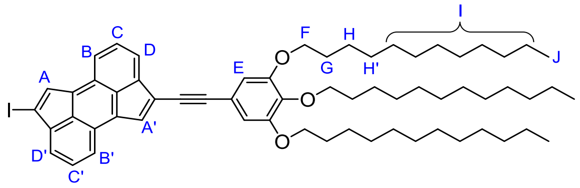

2,7-diiodo CPA[11] (0.66 g, 1.72 mmol), $Pd(PPh_3)_4$ (200 mg, 0.17 mmol), CuI (33 mg, 0.17 mmol) was loaded in a 500 mL round-bottomed flask and three cycles of vacuum/argon backfilling were applied. Next, freshly distilled and degassed THF (280 mL) and NEt_3 (100 mL) were added under argon. Afterwards, compound **2a** (1.05 g, 1.6 mmol) was dissolved in dry THF (20 mL) and slowly added dropwise to the reaction mixture. Finally, the mixture was stirred under argon atmosphere at 35 °C overnight. After this time, the solvent was evaporated under reduced pressure and the crude product was subjected to column chromatography (SiO_2, *n*-heptane/$CHCl_3$ 4:1 v/v). The solvents were removed under reduced pressure and the crude product was subjected to size exclusion chromatography (BioBeads) in THF. The solvent was removed *in vacuo* and the crude suspended in MeOH and sonicated. The resulting suspension was filtered, and the residue washed with MeOH (10 mL) and dried under vacuum to give **3a** as a dark green solid (yield: 375 mg, 32%).

^{1}H-NMR (400 MHz, 25 ºC, $CDCl_3$) δ (ppm) 8.23 (d, *J* = 8.3 Hz, 1H), 8.15 (d, *J* = 8.3 Hz, 1H), 8.01 (d, *J* = 6.4 Hz, 1H), 7.83 (d, *J* = 6.4 Hz, 1H), 7.76-7.70 (m, 3H), 7.60 (s, 1H), 6.83 (s, 2H), 4.05-4.01 (m, 6H), 1.86-1.74 (m, 6H), 1.52 (s, 6H), 1.27 (s, 48H), 0.90-0.86 (m, 9H); **^{13}C-NMR** (101 MHz, 25 ºC, $CDCl_3$) δ (ppm) 153.3, 140.6, 139.7, 139.2, 137.2, 137.0, 130.2, 129.5, 129.0, 127.7, 127.4, 127.2, 126.8, 126.2, 125.8, 125.6, 124.0, 123.7, 123.3, 119.4, 118.0, 110.5, 97.7, 84.5, 73.8, 69.4, 32.1, 32.1, 30.5, 29.9, 29.9, 29.9, 29.8, 29.8, 29.6, 29.6, 29.5, 26.3, 22.9, 14.3; **FT-IR** (ATR) ν (cm^{-1}): 2913, 2844, 2182, 1570, 1509, 1463, 1429, 1371, 1341, 1280, 1231, 1113, 1025, 984, 896, 785, 740, 713, 656, 618; **MS** (MALDI, DCTB) 1004.5-1008.6 m/z $[M]^{\bullet+}$; Chemical formula and formula weight of **3a**: $C_{62}H_{85}IO_3$ and 1005.26; **UV-vis** ($CHCl_3$): λ_{max} (log ε) =660 (4.36), 621 (4.36), 468 (4.36), 440 (4.44), 389 (4.60), 306 (4.90); **R_f** = 0.40 (*n*-heptane/$CHCl_3$ 4:1 v/v).

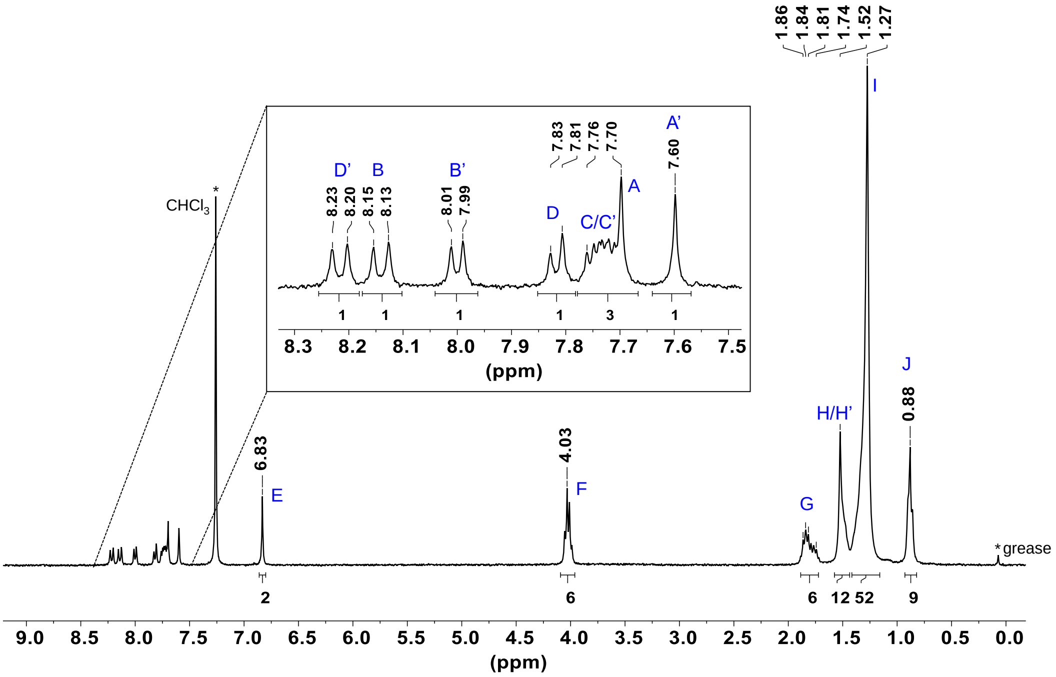

Figure S2.1. ^{1}H-NMR spectrum (25 ºC) of CPA derivative **3a** in $CDCl_3$. Letters refer to protons' assignment of **3a** in the structure above.

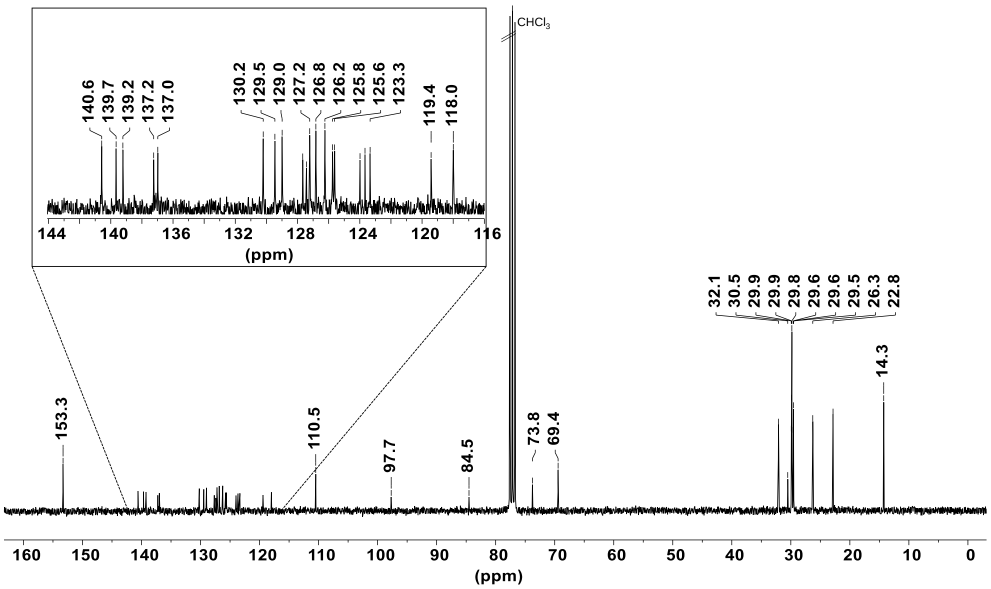

Figure S2.2. ^{13}C-NMR spectrum (25 ºC) of CPA derivative **3a** in $CDCl_3$.

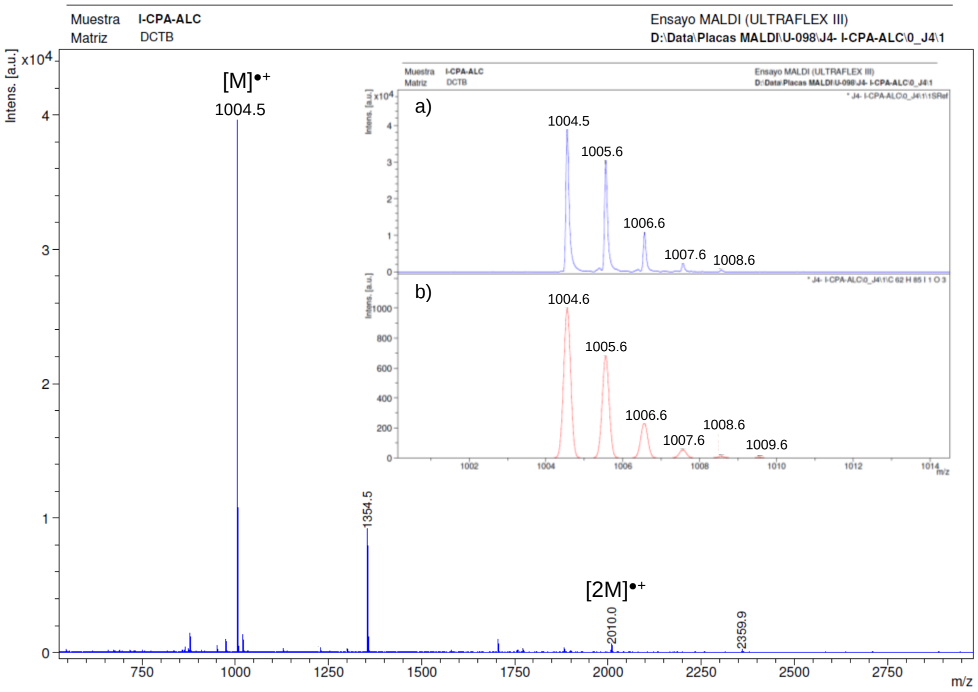

Figure S2.3. MALDI-TOF MS spectrum of CPA derivative **3a**. Insets: a) isotopic distribution of the APCI peaks between 1000 and 1014 m/z; b) Calculated isotopic pattern for **3a**.

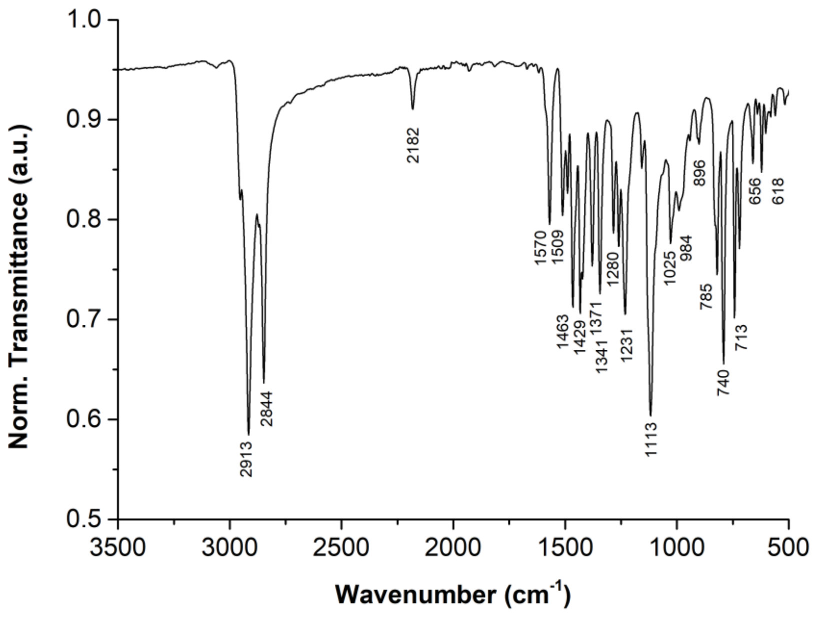

Figure S2.4. FT-IR (ATR) MS spectrum of CPA derivative **3a**.

Synthesis and characterization of CPA derivative **5a**

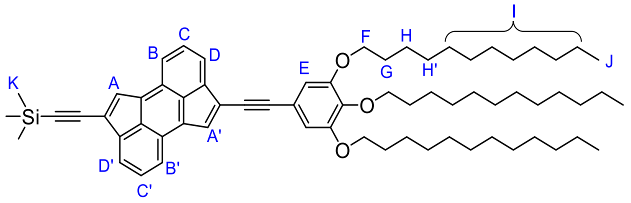

Compound **3a** (100 mg, 0.1 mmol), $Pd(PPh_3)_4$ (7.3 mg, 0.01 mmol), CuI (2.1 mg, 0.01 mmol) were dissolved under argon atmosphere in freshly distilled and degassed THF (15 mL) and NEt_3 (5 mL). Next, trimethylsylysacetylene (15 mg, 0.15 mmol) was added and the whole mixture was stirred at r.t. overnight. After this time, the solution was reduced to dryness under reduced pressure and the crude was purified by column chromatography (SiO_2, *n*-heptane/$CHCl_3$ 4:1 v/v). The solvents were removed under reduced pressure and the crude product was subjected to size exclusion chromatography (BioBeads) in THF. The solvent was eliminated under reduced pressure and the crude suspended in MeOH and sonicated. The resulting suspension was filtered, and the residue washed with MeOH (5 mL) and dried under vacuum to give **5a** as a dark green solid (yield: 82 mg, 80%).

^{1}H-NMR (400 MHz, 25 ºC, $CDCl_3$) δ (ppm) 8.18 (dd, *J* = 8.3, 2.1 Hz, 2H), 8.00 (d, *J* = 6.5 Hz, 1H), 7.93 (d, *J* = 6.9 Hz, 1H), 7.76-7.66 (m, 4H), 6.83 (s, 2H), 4.02 (m, 6H), 1.88-1.72 (m, 6H), 1.55-1.46 (m, 6H), 1.27 (s, 48H), 0.90-0.86 (m, 9H), 0.34 (s, 9H); **^{13}C-NMR** (101 MHz, 25 ºC, $CDCl_3$) δ (ppm) 153.3, 140.5, 139.7, 138.7, 137.6, 131.3, 130.1, 129.4, 128.2, 128.1, 126.4, 126.3, 125.7, 125.5, 124.6, 124.4, 123.7, 123.0, 118.0, 110.5, 102.8, 100.7, 97.9, 84.6, 73.8, 69.4, 32.1, 32.1, 30.5, 29.9, 29.9, 29.9, 29.8, 29.8, 29.6, 29.6, 29.5, 26.3, 22.8, 14.3, 0.3; **FT-IR** (ATR) ν (cm^{-1}): 2955, 2917, 2848, 2182, 2132, 1569, 1513, 1486, 1463, 1433, 1380, 1337, 1276, 1235, 1113, 1056, 1022, 927, 854, 831, 789, 744, 713, 641, 538; **MS** (MALDI-TOF, DCTB): 974.8-978.8 m/z $[M]^{\bullet+}$; Chemical formula and formula weight of **5a**: $C_{67}H_{94}O_3Si$ and 975.57; **UV-vis** ($CHCl_3$): λ_{max} (log ε) =669 (4.39), 626 (4.38), 476 (4.34), 449 (4.41), 397 (4.51), 307 (4.89); **R_f** = 0.38 (*n*-heptane/$CHCl_3$ 4:1 v/v).

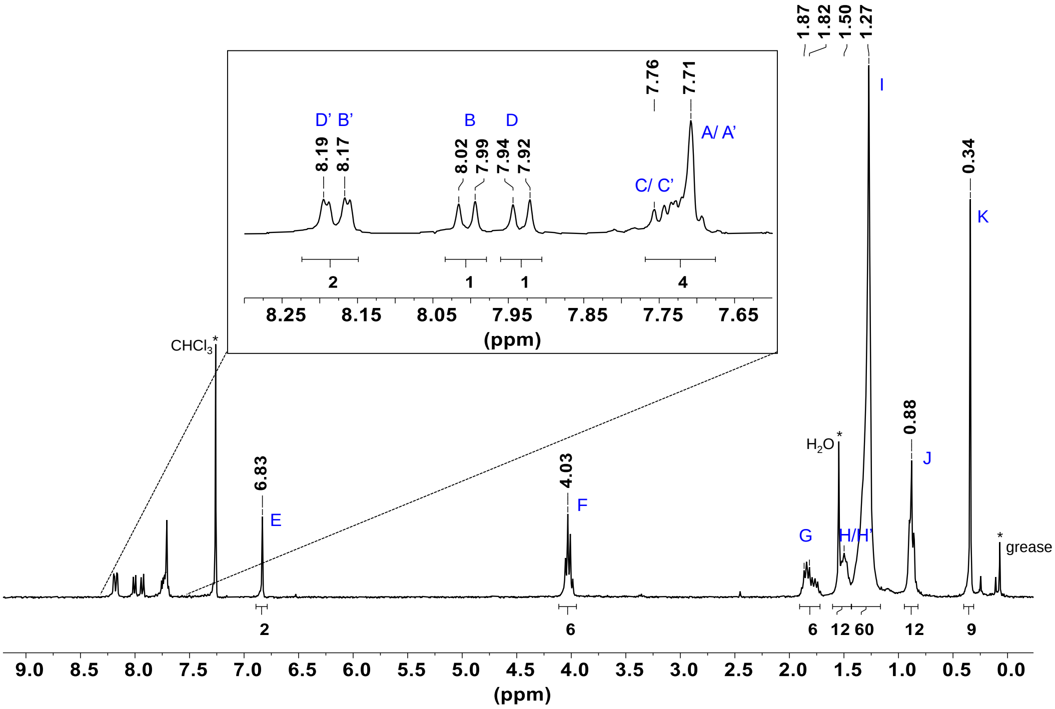

Figure S2.5. ^{1}H-NMR spectrum (25 ºC) of CPA derivative **5a** in $CDCl_3$. Letters refer to protons' assignment of **5a** in the structure above.

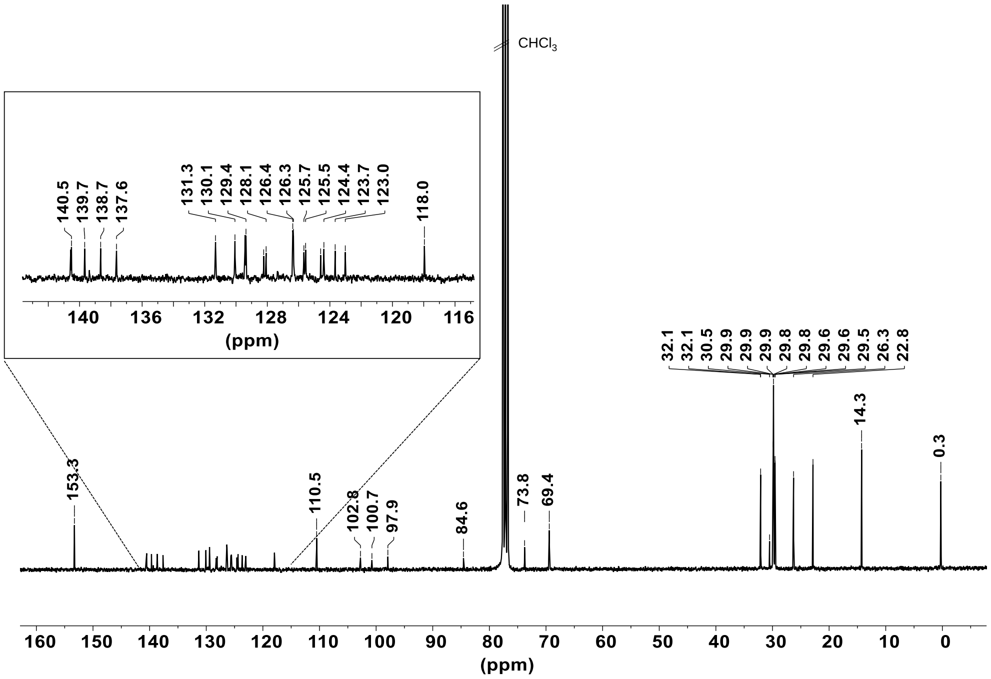

Figure S2.6. ^{13}C-NMR spectrum (25 ºC) of CPA derivative **5a** in $CDCl_3$.

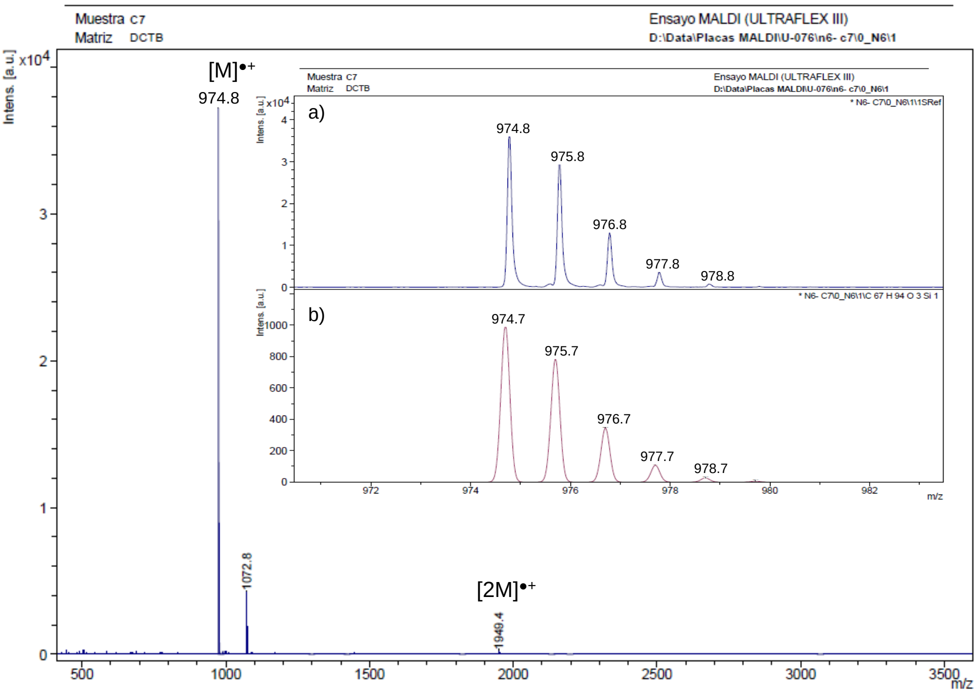

Figure 2.7. APCI MS spectrum of CPA derivative **5a**. Insets: a) isotopic distribution of the APCI peaks between 971 and 983 m/z; b) calculated isotopic pattern for **5a**.

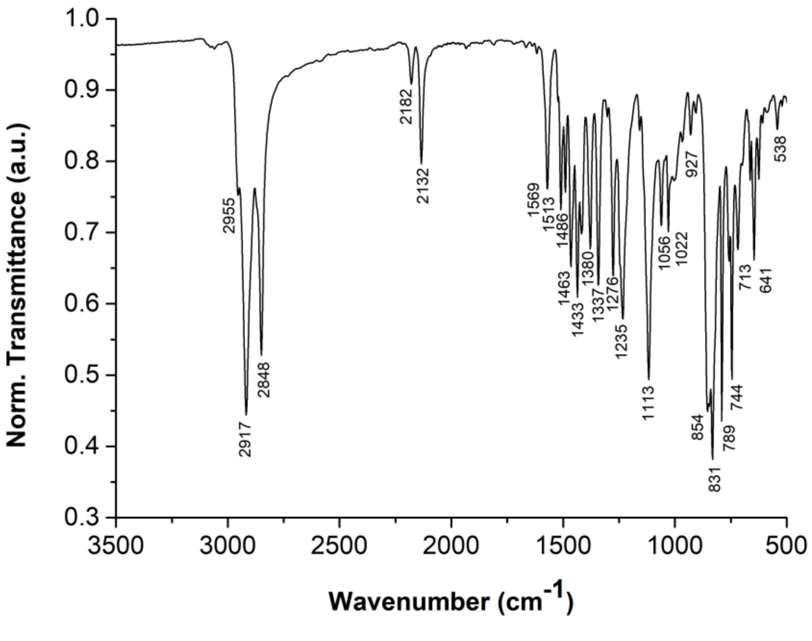

Figure S2.8. FT-IR (ATR) spectrum of CPA derivative **5a**.

*Synthesis and characterization of CPA dimer **1a***

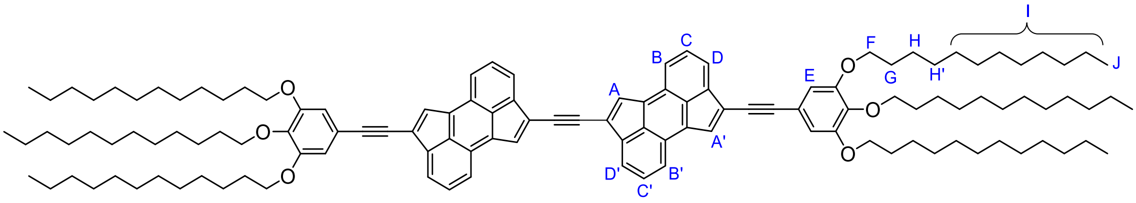

Compound **5a** (9.74 mg, 0.01 mmol) was dissolved in a mixture of DCM (6 mL) and MeOH (3 mL) and K_2CO_3 (7 mg, 0.05 mmol) added while stirring at r.t. for 3 h. After this time, the solution was extracted with DCM (10 mL) and the organic layer subsequently washed with water (2 × 5 mL), dried over $MgSO_4$, and the filtrate concentrated under reduced pressure. The resulting crude was dissolved in dried and degassed THF (2 mL) and the solution was poured dropwise into a mixture of **3a** (9.56 mg, 0.01 mmol), $Pd(PPh_3)_4$ (0.12 mg, 0.0001 mmol), CuI (0.02 mg, 0.0002 mmol), THF (4 mL) and NEt_3 (2 mL). The resulting mixture was stirred at r.t. for 2 h. After this time, the solvents were evaporated under vacuum, and the resulting solid purified by column chromatography (*n*-heptane/$CHCl_3$ (from 3:1 to 1:1 v/v)). The solvent was removed under reduced pressure and the crude subjected to size exclusion chromatography (BioBeads) in THF. The solvent was removed under reduced pressure and the crude suspended in MeOH and sonicated. The suspension was then filtered, and the solid washed with MeOH (3 mL) and dried under vacuum to yield CPA dimer **1a** as a dark green solid (yield: 4.8 mg, 27%).

An alternative and higher yielding synthesis of CPA dimer **1a** was also investigated with the aim of overcoming the poor stability of trimethylsylyethynyl-terminated CPA derivative **5a**. In this synthetic route, compound **3a** (10 mg, 0.01 mmol), $Pd(PPh_3)_4$ (1.15 mg, 0.001 mmol), CuI (0.38 mg, 0.002 mmol) were dissolved under argon atmosphere in freshly distilled and degassed THF (6 mL) and NEt_3 (2 mL). Next, trimethylsylysacetylene (7 µL, 0.05 mmol) was added and the reaction mixture stirred at r.t. for 16 hrs. After this time, a TLC was performed showing the disappearance of starting material **3a** and the formation of a new spot corresponding to compound **5a**. Next, K_2CO_3 (6.91 mg, 0.05 mmol) and MeOH (3 mL) were added to the reaction mixture and the solution stirred at r.t. for 1 hr. After this time, a TLC was performed showing the disappearance of the *in-situ* generated **5a** and the formation of a new spot attributable to the corresponding ethynyl-terminated CPA derivative. Next, the reaction mixture was transferred into a separating funnel and H_2O (10 mL) and DCM (10 mL) added. Upon vigorous shaking, the organic and aqueous layers were separated, and the aqueous phase extracted with DCM (2 × 10 ml). The organic phases were combined, washed with brine (1 × 10 mL), dried over

$MgSO_4$, and filtered. The filtrate was concentrated under reduced pressure and the resulting crude was dissolved in dried and degassed THF and the solution poured dropwise into a mixture of **3a** (10 mg, 0.01 mmol), $Pd(PPh_3)_4$ (1.15 mg, 0.001 mmol), CuI (0.38 mg, 0.002 mmol), THF (6 mL) and NEt_3 (2 mL). The resulting mixture was stirred at r.t. for 2 h. After this time, the solvents were evaporated *in vacuo*, and the resulting solid was purified by column chromatography (*n*-heptane/DCM (from 4:1 to 1:1 v/v)). The solvent was removed under reduced pressure and the crude product was subjected to size exclusion chromatography (BioBeads) in THF. The solvent was removed under reduced pressure and the crude solid was suspended in MeOH, sonicated, filtered, washed with MeOH and dried under vacuum to yield CPA dimer **1a** as a dark green solid (yield: 4.8 mg, 27%, calculated from the number of mmol of starting material **3a**).

^{1}H-NMR (400 MHz, 25 ºC, $CDCl_3$): δ (ppm) 8.21 (d, *J* = 8.3 Hz, 1 H), 8.11 (d, *J* = 6.7 Hz, 1 H), 8.01 (d, *J* = 6.7 Hz, 1H), 7.81 (s, 3H), 7.78 (d, *J* = 1.7 Hz, 1H), 7.76 (d, *J* = 1.9 Hz, 1H), 7.74 (d, *J* = 1.7 Hz, 1 H), 7.71 (s, 2H), 6.84 (s, 4H), 4.05-3.99 (m, 12H), 1.87-1.75 (m, 12H), 1.55-1.28 (m, 108H), 0.90-0.86 (m, 18 H); **^{13}C-NMR** (125 MHz, 25 ºC, $CDCl_3$): δ (ppm)= 153.3, 140.5, 140.4, 139.6, 138.5, 138.4, 135.4, 130.4, 130.0, 129.4, 129.4, 128.6, 128.4, 127.8, 126.5, 126.4, 125.6, 124.5, 124.4, 123.6, 123.5, 118.0, 110.4, 98.1, 94.8, 84.8, 73.8, 69.4, 53.6, 32.1, 30.5, 29.9, 29.9, 29.9, 29.9, 29.8, 29.8, 29.6, 29.6, 29.5, 26.3, 22.9, 14.3; **FT-IR** (ATR) ν (cm^{-1}): 2962, 2916, 2844, 2175, 1570, 1505, 1467, 1436, 1413, 1375, 1337, 1253, 1227, 1090, 1010, 861, 785, 763, 717, 663, 618; **MS** (MALDI-TOF, DCTB): 1779.3-1785.4 m/z $[M]^{•+}$; Chemical formula and formula weight of **1a**: $C_{126}H_{170}O_6$ and 1780.74; **UV-vis** ($CHCl_3$): $λ_{max}$ (log ε) =808 (4.57), 739 (4.61), 484 (4.29), 456 (4.34), 395 (4.42), 322 (4.86); **R_f** = 0.35 (*n*-heptane/$CHCl_3$ 2:1 v/v).

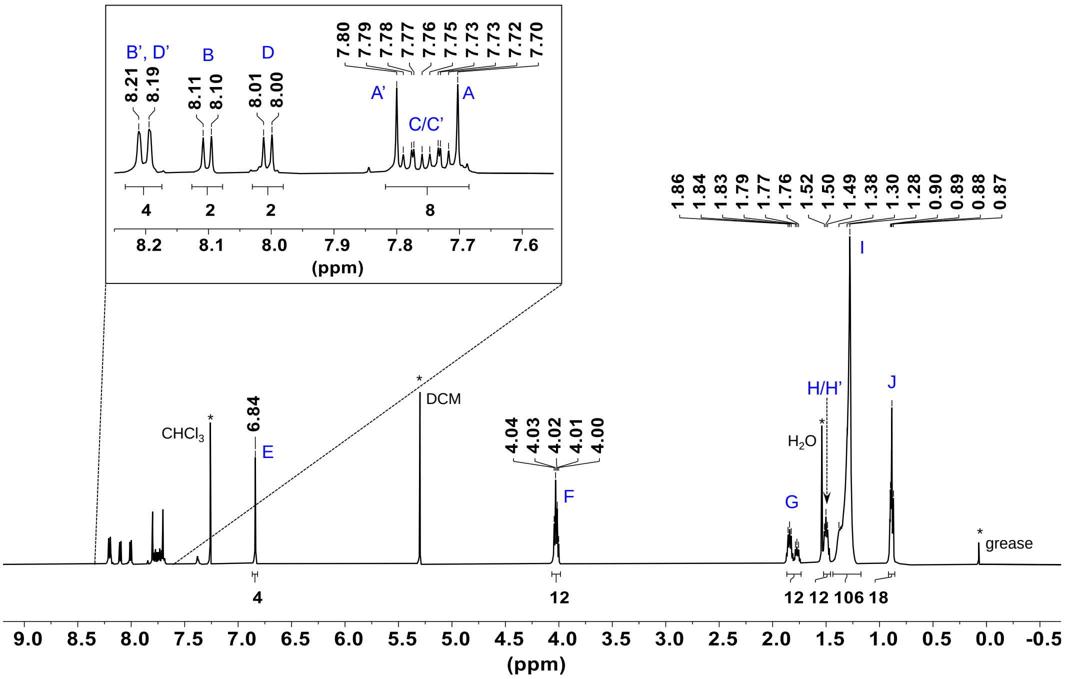

Figure S2.9. ^{1}H-NMR spectrum (25 ºC) of CPA dimer **1a** in $CDCl_3$. Letters refer to protons' assignment of **1a** in the structure above.

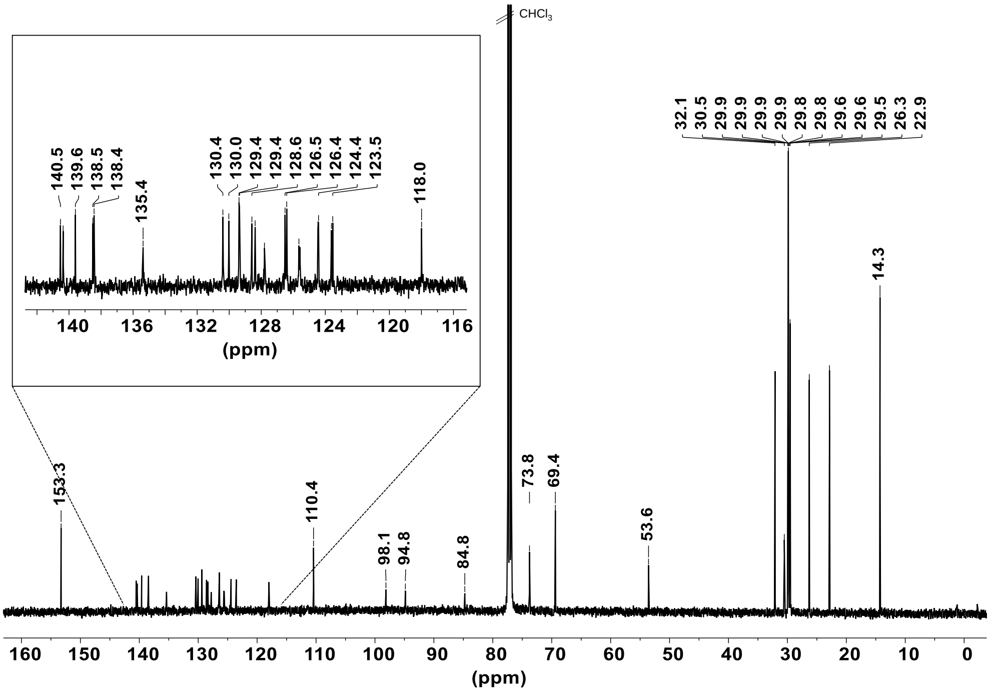

Figure S2.10. ^{13}C-NMR spectrum (25 ºC) of CPA dimer **1a** in $CDCl_3$.

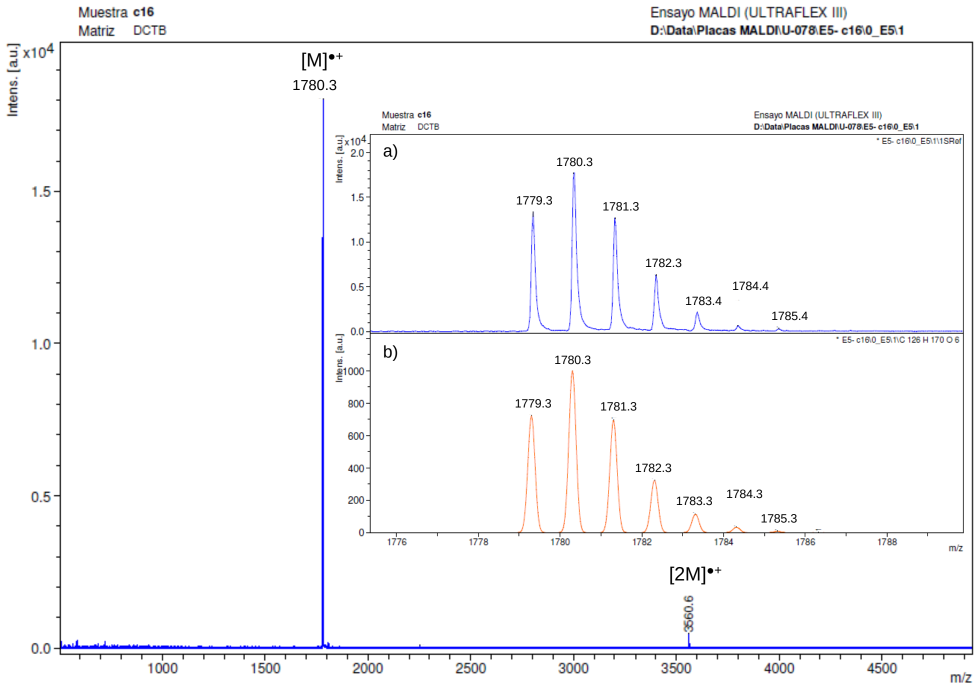

Figure S2.11. MALDI-TOF MS spectrum of CPA dimer **1a**. Inset: a) isotopic distribution of the peaks between 1776 and 1789 m/z; b) calculated isotopic pattern for **1a**.

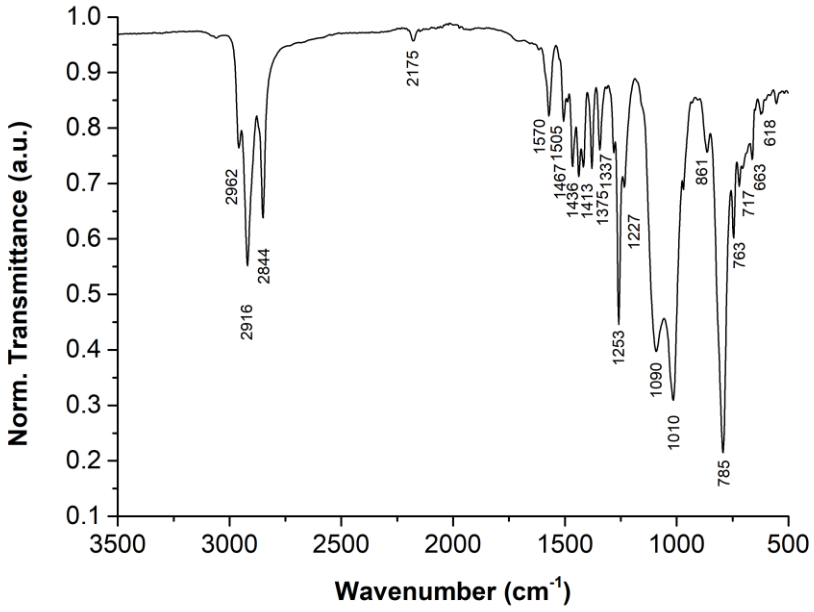

Figure S2.12. FT-IR (ATR) spectrum of CPA dimer **1a**.

*Synthesis and characterization of CPA derivative **3b***

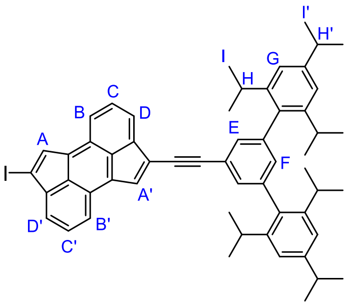

2,7-diiodo CPA[11] (300 mg, 0.63 mmol), $Pd(PPh_3)_4$ (76 mg, 0.063 mmol), CuI (26 mg, 0.13 mmol) was loaded in a 250 mL round-bottomed flask and three cycles of vacuum/argon backfilling were applied. Next, freshly distilled and degassed THF (110 mL) and NEt_3 (40 mL) were added under argon. Afterwards, compound **2b** (250 mg, 0.47 mmol) was dissolved in dry THF (10 mL) and slowly added dropwise to the reaction mixture. Finally, the mixture was stirred under argon atmosphere at 35 °C overnight. After this time, the solvent was evaporated under reduced pressure and the crude product was subjected to column chromatography (SiO_2, *n*-heptane/$CHCl_3$ 6:1 v/v). The solvent was removed under reduced pressure and the crude subjected to size exclusion chromatography (BioBeads) in THF. The solvent was removed under reduced pressure and the crude suspended in MeOH and sonicated. The filtrate was washed with MeOH (5 mL) and the solid dried under vacuum to give **3b** as a dark green solid (yield: 100 mg, 20%).

^{1}H-NMR (400 MHz, 25 ºC, $CDCl_3$) δ (ppm) 8.21 (d, *J* = 8.5 Hz, 1H), 8.14 (d, *J* = 8.5 Hz, 1H), 7.96 (d, *J* = 6.6 Hz, 1H), 7.86 (s, 1H), 7.77-7.69 (m, 3H), 7.64 (d, *J* = 6.6 Hz, 1H), 7.46 (s, 2H), 7.07 (s, 4H), 7.05 (s, 1H), 2.98-2.91 (m, 2 H), 2.78-2.75 (m, 4H), 1.31 (d, *J* = 6.9 Hz, 12H), 1.20 (d, *J* = 6.9 Hz, 12H), 1.07 (d, *J* = 6.9 Hz, 12H); **^{13}C-NMR** (101 MHz, 25 ºC, $CDCl_3$) δ = 141.8, 141.2, 140.5, 138.8, 137.2, 136.1, 134.7, 132.2, 131.9, 131.1, 130.5, 129.7, 129.1, 128.6, 127.7, 127.6, 126.8, 126.3, 124.0, 123.5, 123.3, 123.0, 120.7, 97.9, 89.9, 85.9, 34.5, 30.6, 29.9, 24.5, 24.4, 24.3, 24.2; **FT-IR** (ATR) ν (cm^{-1}): 2955, 2921, 2864, 1607, 1581, 1459, 1429, 1379, 1356, 1310, 1258, 1162, 1098, 1065, 1029, 999, 942, 877, 820, 785, 736, 713, 652, 603, 557; **MS** (MALDI, DCTB) m/z: 856.4-860.4 m/z $[M]^{\bullet+}$; Chemical formula and formula weight of **3b**: $C_{56}H_{57}I$ and 856.98; **UV-vis** ($CHCl_3$): λ_{max} (log ε) = 655 (4.33), 616 (4.34), 469 (4.40), 441 (4.46), 391 (4.61), 315 (4.94); **R_f** = 0.45 (*n*-heptane/$CHCl_3$ 6:1 v/v).

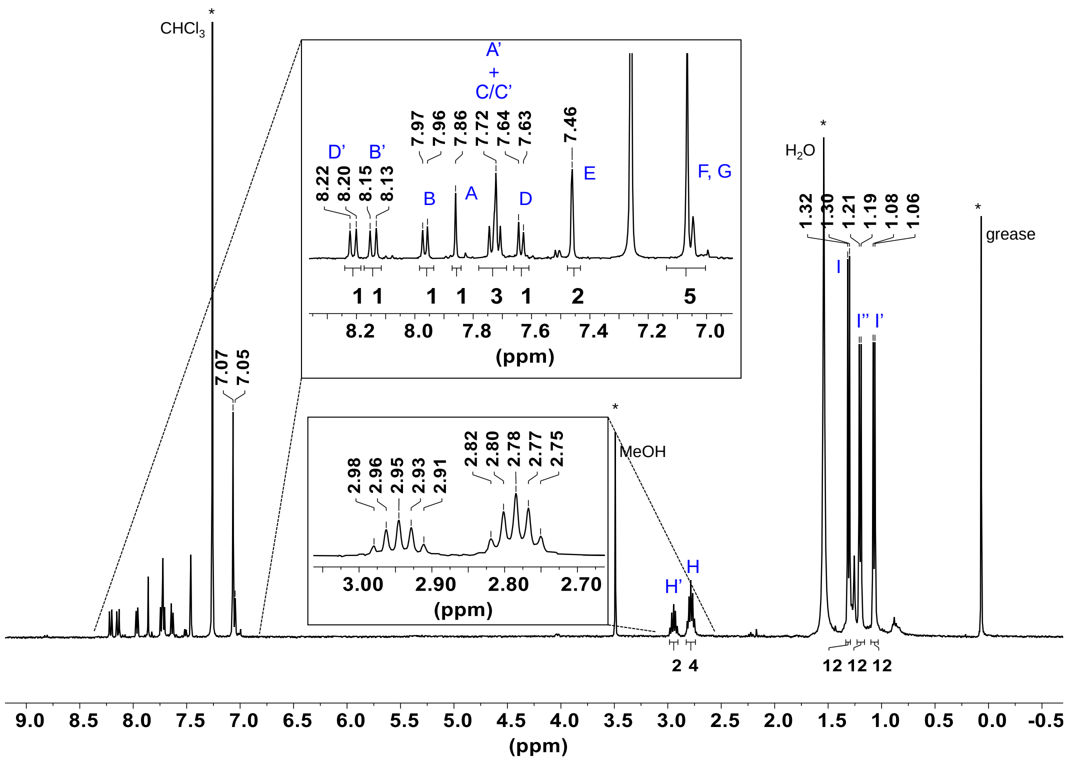

Figure S2.13. ^{1}H-NMR spectrum (25 ºC) of CPA derivative **3b** in $CDCl_3$. Letters refer to protons’ assignment of **3a** in the structure above.

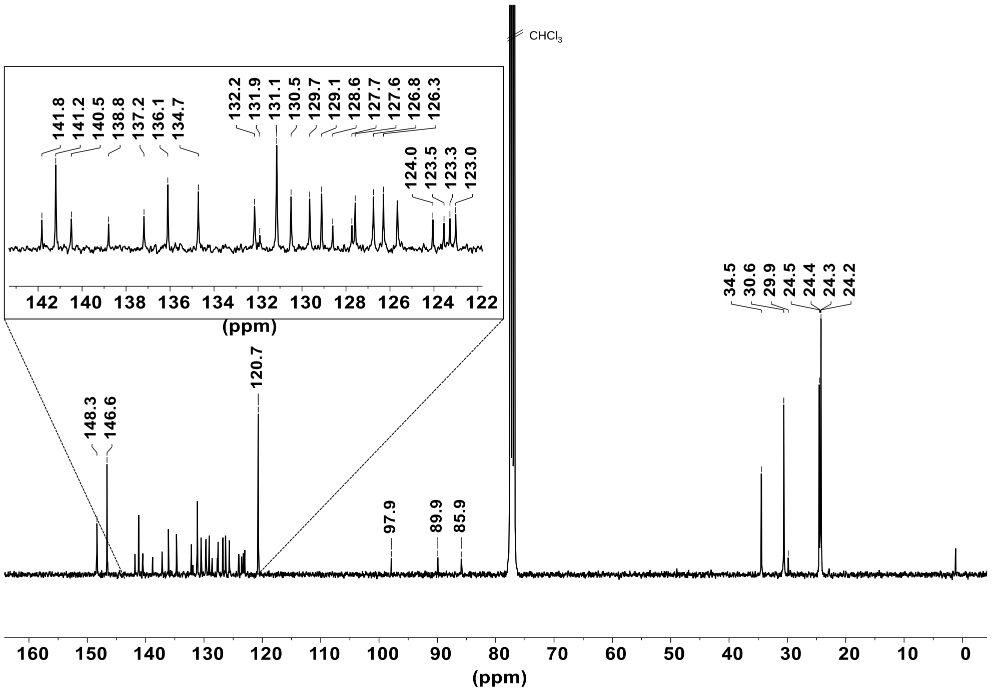

Figure S2.12. ^{13}C-NMR spectrum (25 ºC) of CPA derivative **3b** in $CDCl_3$.

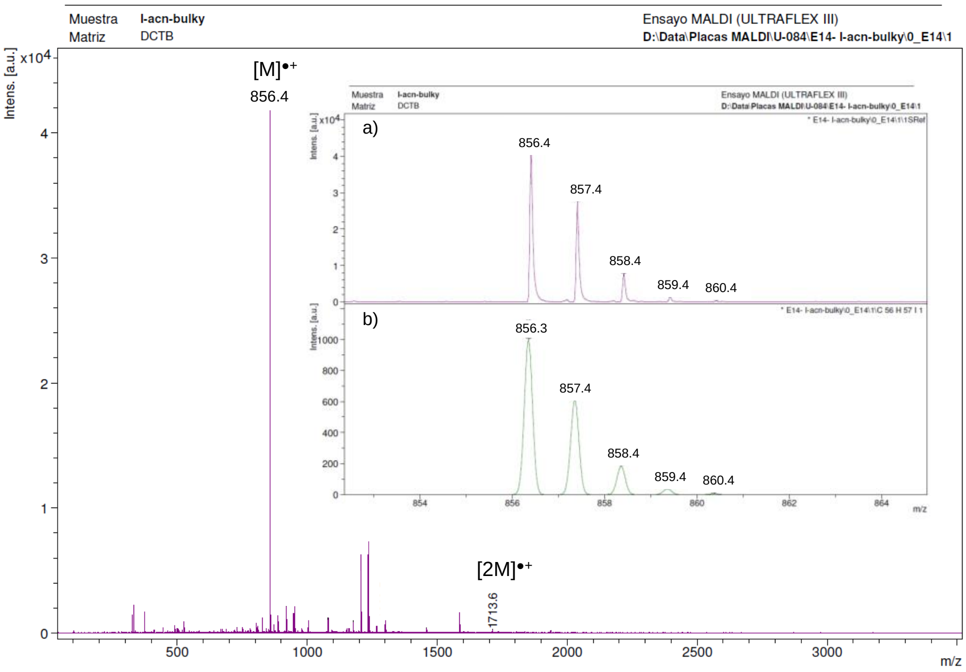

Figure S2.15. MALDI-TOF MS spectrum of CPA derivative **3b**. Insets: a) isotopic distribution of the peaks between 853 and 865 m/z; b) calculated isotopic pattern for **3b**.

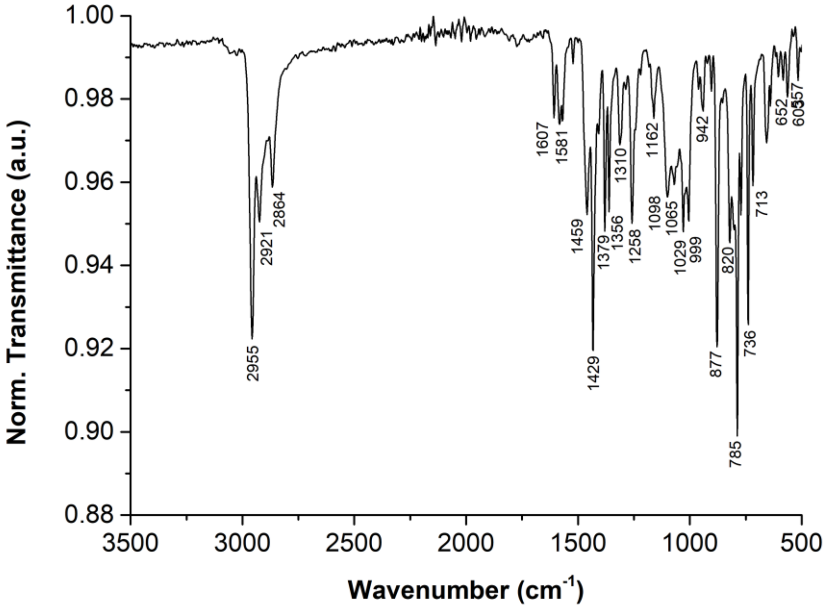

Figure S2.16. FT-IR (ATR) spectrum of CPA derivative **3b**.

*Synthesis and characterization of CPA dimer **1b***

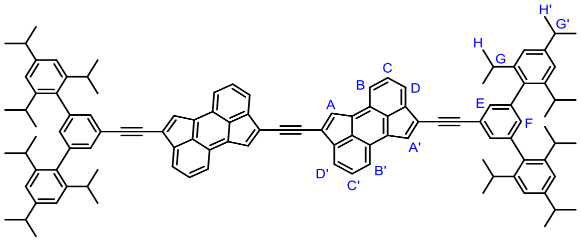

Compound **3b** (30 mg, 0.035 mmol), $Pd(PPh_3)_4$ (4.05 mg, 0.0035 mmol), CuI (1.33 mg, 0.0070 mmol) were dissolved under argon atmosphere in freshly distilled and degassed THF (9 mL) and NEt_3 (3 mL). Next, trimethylsylysacetylene (7 µL, 0.052 mmol) was added and the reaction mixture stirred at r.t. for 16 hrs. After this time, a TLC was performed showing the disappearance of starting material **3b**.* Next, K_2CO_3 (9.70 mg, 0.07 mmol) and MeOH (5 mL) were added to the reaction mixture and the solution stirred at r.t. for 1 hr. After this time, a TLC was performed showing the disappearance of the *in-situ* generated trimethylsylyethynyl-terminated CPA species and the formation of a new spot attributable to the corresponding ethynyl-terminated CPA derivative. Next, the reaction mixture was transferred into a separating funnel and H_2O (10 mL) and DCM (10 mL) added. Upon vigorous shaking, the organic and aqueous layers were separated, and the aqueous phase extracted with DCM (2 × 10 ml). The organic phases were combined, washed with brine (1 × 10 mL), dried over $MgSO_4$, and filtered. The filtrate was concentrated under reduced pressure and the resulting crude was dissolved in dried and degassed THF and the solution poured dropwise into a mixture of **3b** (30 mg, 0.035 mmol), $Pd(PPh_3)_4$ (4.05 mg, 0.0035 mmol), CuI (1.33 mg, 0.007 mmol), THF (9 mL) and NEt_3 (3 mL). The resulting mixture was stirred at r.t. for 2 h. After this time, the solvents were evaporated *in vacuo*, and the resulting solid was purified by column chromatography (*n*-heptane/DCM (from 4:1 to 1:1 v/v)). The solvent was removed under reduced pressure and the crude product was subjected to size exclusion chromatography (BioBeads) in THF. The solvent was removed under reduced pressure and the crude solid was suspended in MeOH, sonicated, filtered, washed with MeOH and dried under vacuum to yield CPA dimer **1b** as a dark green solid (yield: 9.4 mg, 18%).

* The synthesis of CPA dimer **1b** had to be carried out from compound **3b** following a one-pot strategy due to stability problems related with the trimethylsylyethynyl-terminated and ethynyl-terminated CPA species. The yield of CPA dimer **1b** has been calculated taking into account the number of mmoles of starting material **3b** used (*i.e.*, 0.035).

^{1}H-NMR (400 MHz, 25 ºC, $CDCl_3$): δ (ppm) 8.24 (dd, *J* = 8.4, 2.5 Hz, 4H), 8.13 (d, *J* = 6.6 Hz, 2H), 7.99 (d, *J* = 6.6 Hz, 2H), 7.87-7.70 (m, 8H), 7.48 (s, 4H), 7.08 (s, 10H), 2.95 (q, *J* = 6.9 Hz, 5H), 2.80 (dt, *J* = 13.7, 6.9 Hz, 10H), 1.32 (d, *J* = 6.9 Hz, 24H), 1.21 (d, *J* = 6.8 Hz, 24H), 1.08 (d, *J* = 6.8 Hz, 24H); **^{13}C-NMR** (125 MHz, 25 ºC, $CDCl_3$): δ (ppm) 148.4, 146.6, 141.2, 140.5, 140.4, 138.6, 138.5, 136.1, 132.2, 131.2, 130.5, 130.4, 129.4, 128.6, 128.5, 126.6, 126.5, 125.7, 125.6, 124.5, 124.5, 123.7, 123.4, 123.0, 120.7, 98.2, 97.9, 94.8, 86.1, 34.5, 30.6, 29.9, 24.5, 24.3, 24.3, 1.2; **FT-IR** (ATR) ν (cm^{-1}): 2955, 2913, 2867, 1603, 1584, 1463, 1429, 1387, 1356, 1311, 1246, 1227, 1212, 1147, 1113, 1021, 927, 881, 858, 766, 736, 618, 572; **MS** (MALDI-TOF, DCTB): 1482.9-1487.9 m/z $[M]^{\bullet +}$; Chemical formula and formula weight of **1b**: $C_{114}H_{114}$ and 1484.17; **UV-vis** ($CHCl_3$): λ_{max} (log ε) = 805 (4.54), 741 (4.58), 483 (4.33), 453 (4.35), 399 (4.67), 326 (4.86); **R$_f$** = 0.55 (*n*-heptane/$CHCl_3$ 2:1 v/v).

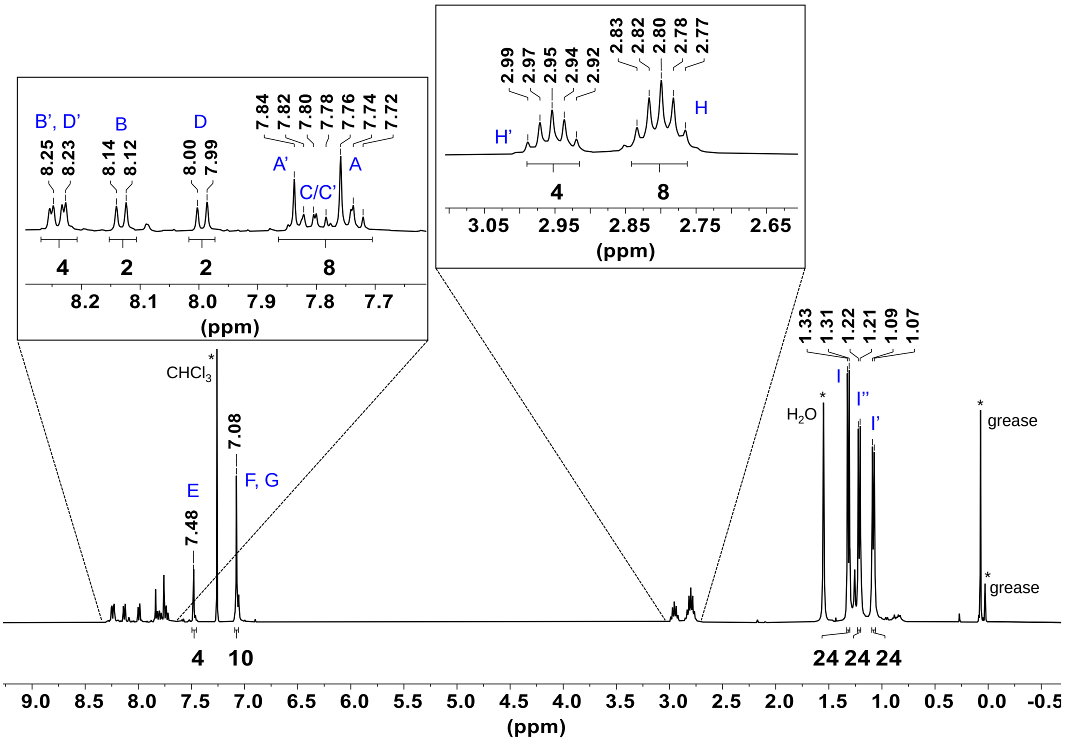

Figure S2.17. ^{1}H-NMR spectrum (25 ºC) of CPA dimer **1b** in $CDCl_3$. Letters refer to protons' assignment of **1b** in the structure above.

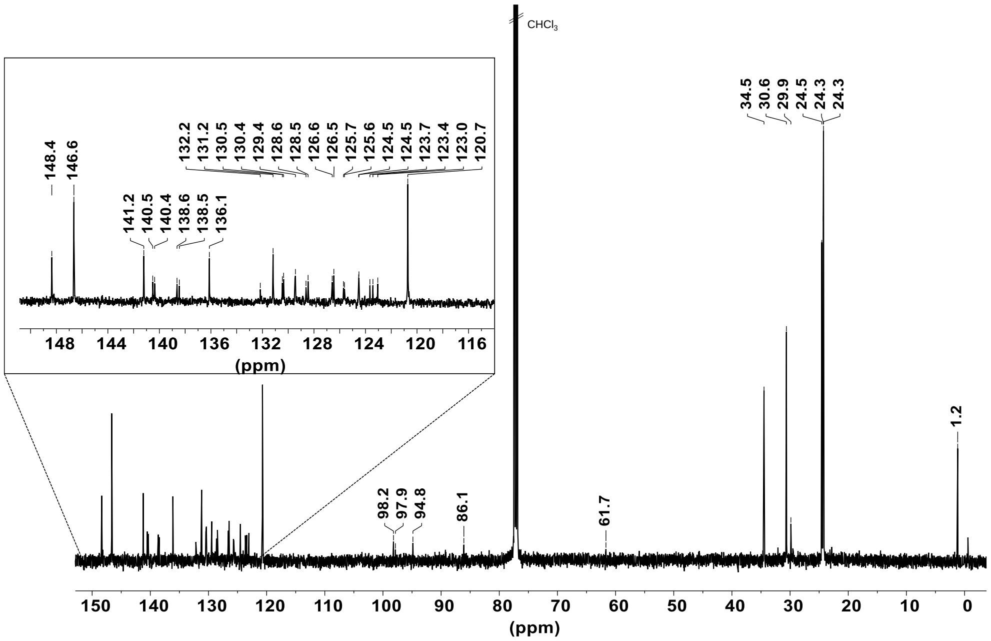

Figure S2.18. ^{13}C-NMR spectrum (25 ºC) of CPA dimer **1b** in $CDCl_3$.

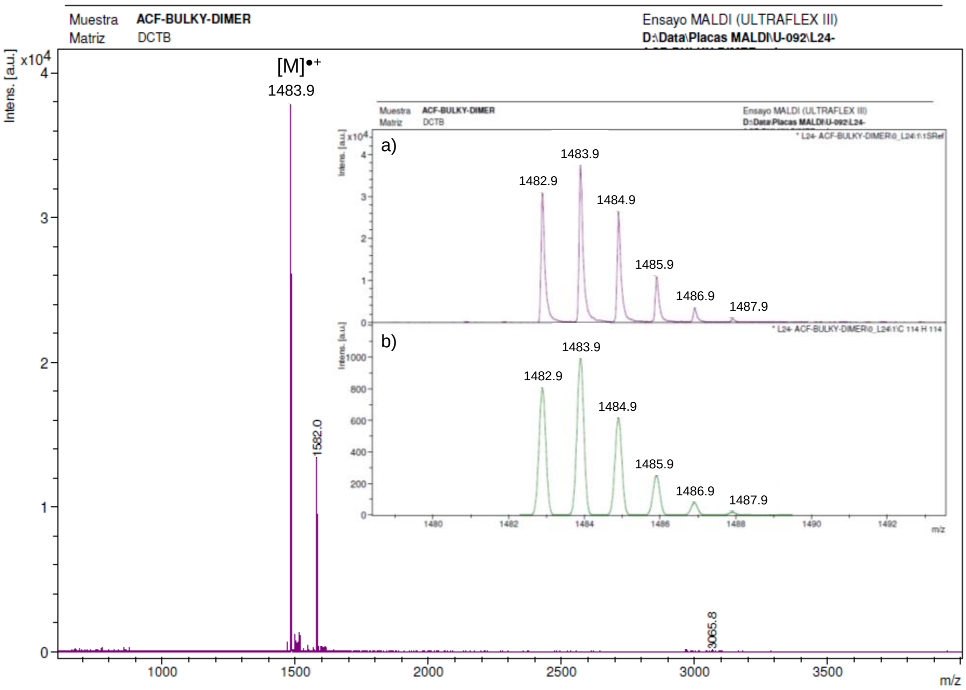

Figure S2.19. MALDI-TOF MS spectrum of CPA dimer **1b**. Insets: a) isotopic distribution of the peaks between 1479 and 1493 m/z; b) Calculated isotopic pattern for **1b**.

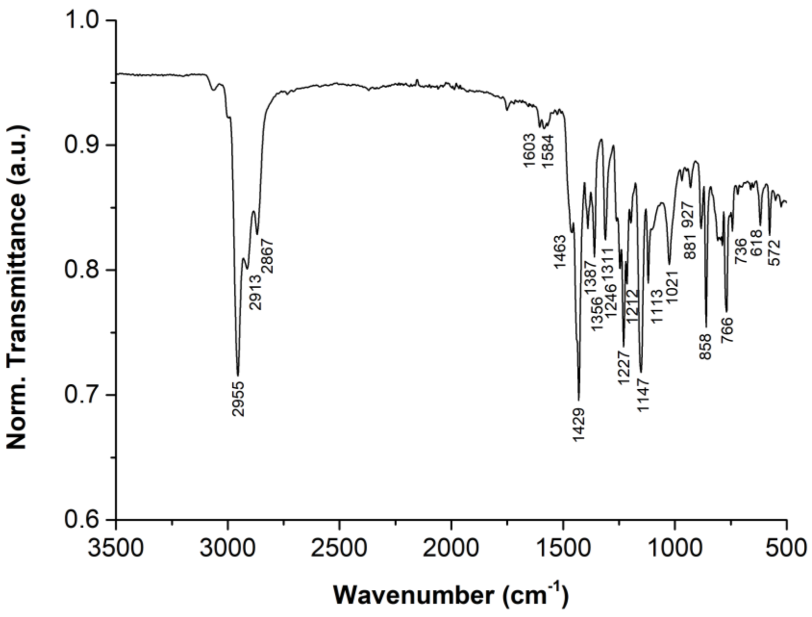

Figure S2.20. FT-IR (ATR) spectrum of CPA dimer **1b**.

3. Steady state and time resolved UV-vis absorption studies on CPA dimers 1a and 1b and reference compounds, electrochemistry of CPA dimer 1a, and and spectroelectrochemistry (UV-vis-NIR and IR electronic absorption) of CPA dimer 1a and CPA monomer 4a

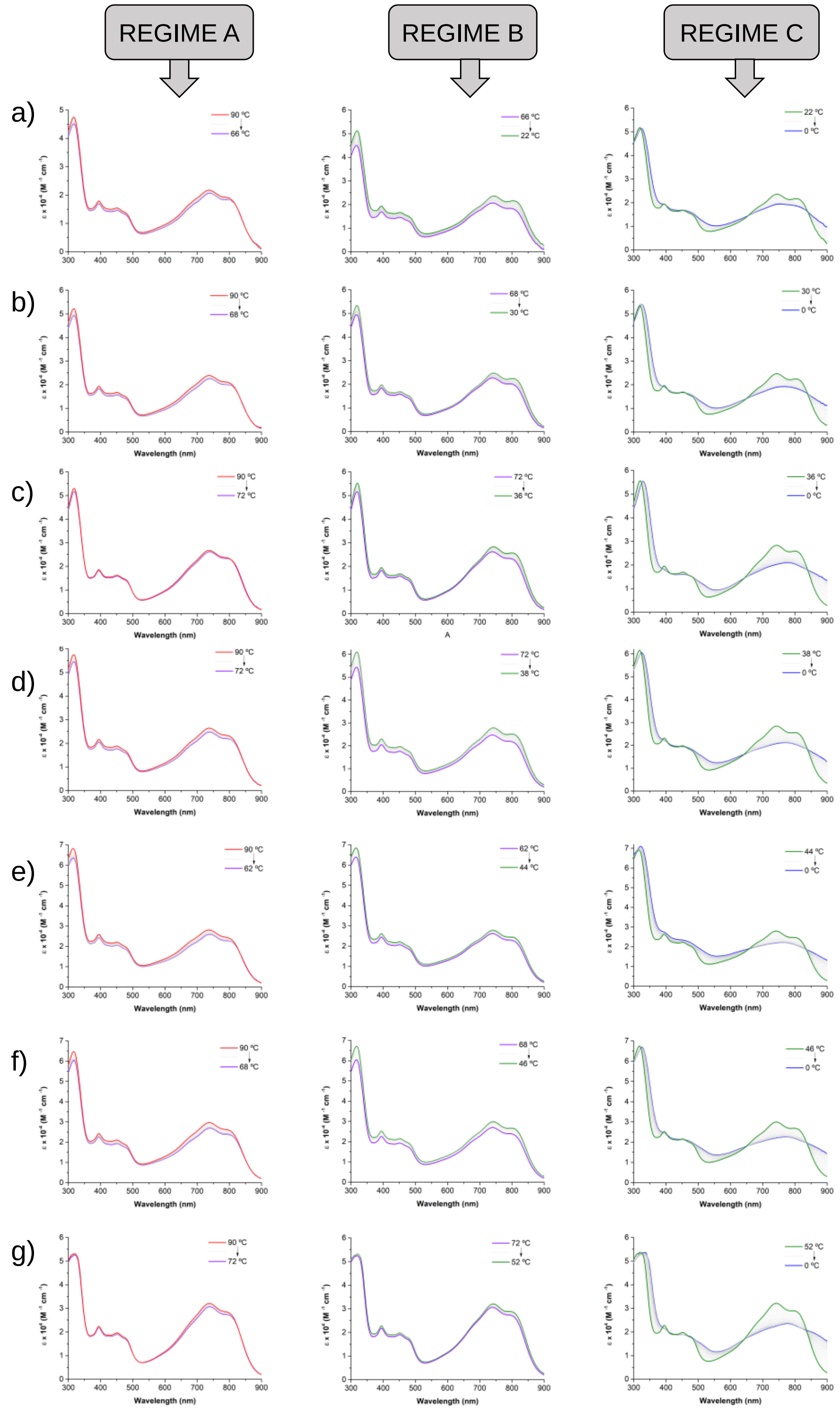

Figure S3.1. VT UV-vis absorption spectra of CPA dimer **1a** in MCH between 90 and 0 ºC at seven different concentrations (a) 6.5 × 10^{-6} M; b) 1.3 × 10^{-5} M; c) 2.0 × 10^{-5} M; d) 2.6 × 10^{-5} M; e) 3.9 × 10^{-5} M; f) 5.2 × 10^{-5} M; g) 7.8 × 10^{-5} M). Intermediate spectra recorded every 2 ºC (dashed grey lines). Cooling rate: 0.2 ºC/min. The three different spectral "regimes" (*i.e.*, regime A (left-handed stacked spectra), regime B (centered stacked spectra), and regime C (right-handed stacked spectra)) are also indicated.

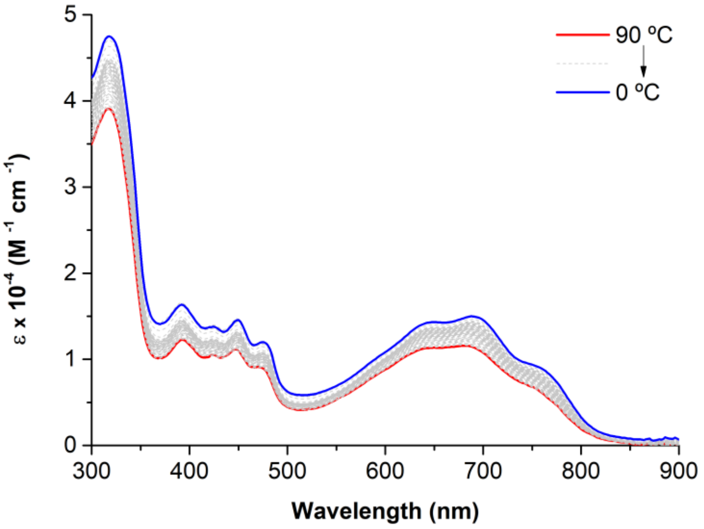

Figure S3.2. VT-UV-vis absorption spectra of CPA monomer **4a** in MCH ([**4a**] = 1.3 × 10^{-5} M) between 90 (red line) and 0 ºC (blue line). Intermediate spectra recorded every 2 ºC (dashed grey lines). Cooling rate: 0.2 ºC/min.

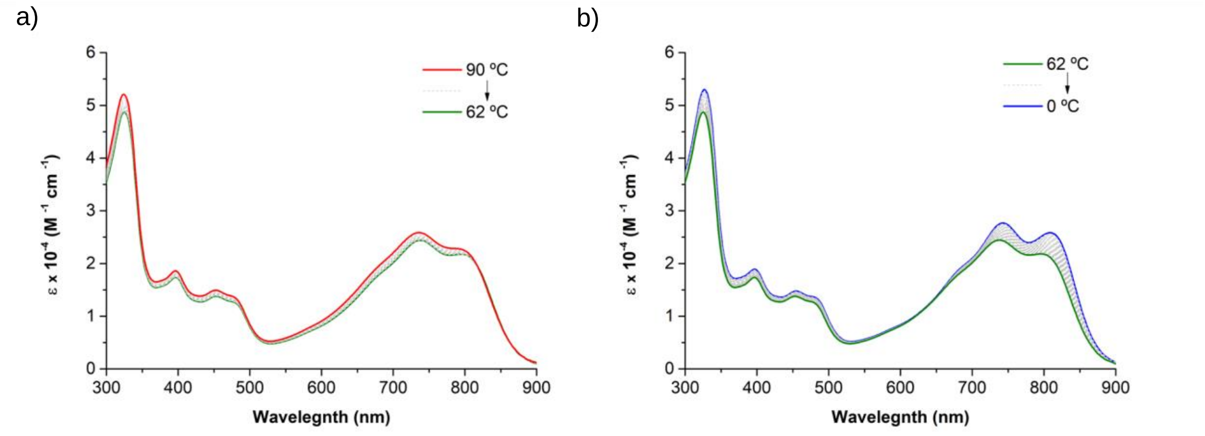

Figure S3.3. VT UV-vis absorption spectra of CPA dimer **1b** in MCH ([**1b**] = 5.2 × 10^{-5} M) between a) 90 (red line) and 62 ºC (green line), and b) 62 (green line) and 0 ºC (blue line). Intermediate spectra in a) and b) recorded every 2 ºC (dashed grey lines). Cooling rate: 0.2 ºC/min.

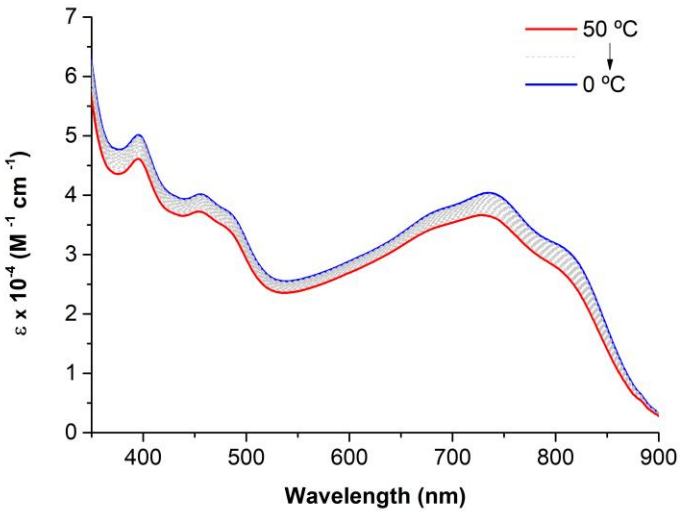

Figure S3.4. VT UV-vis absorption spectra of CPA dimer **1a** in $CHCl_3$ ([**1a**] = 5.2 × 10^{-5} M) between 50 (red line) and 0 ºC (blue line). Intermediate spectra recorded every 2 ºC (dashed grey lines). Cooling rate: 0.2 ºC/min.

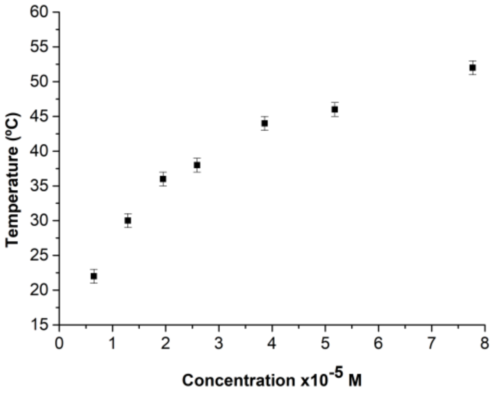

Concentration	Temperature
6.5 × 10^{-6} M	22 ºC
1.3 × 10^{-5} M	30 ºC
2.0 × 10^{-5} M	36 ºC
2.6 × 10^{-5} M	38 ºC
3.9 × 10^{-5} M	44 ºC
5.2 × 10^{-5} M	46 ºC
7.8 × 10^{-5} M	52 ºC

Figure S3.5. Changes in the upper temperature at which regime C sets in (*i.e.*, the transition temperature between regimes B and C) in **1a** in MCH as a function of the CPA dimer concentration.

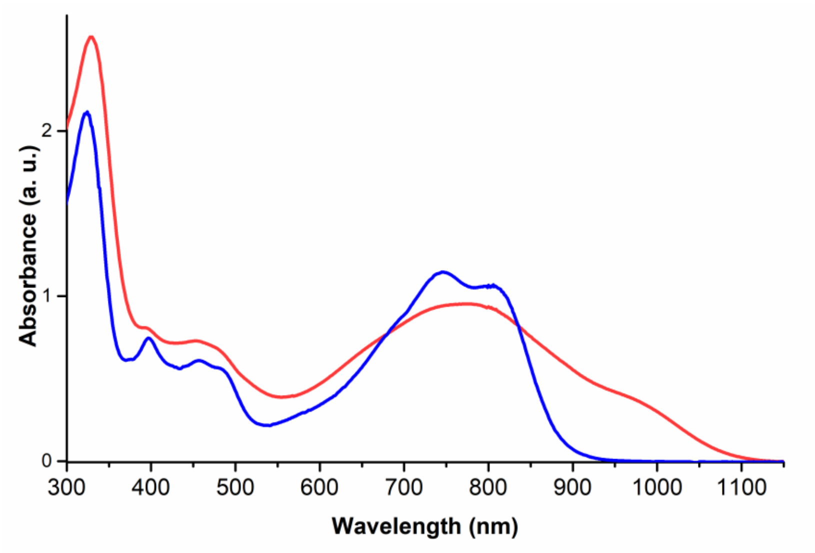

Figure S3.6. UV-vis-NIR absorption spectra of CPA dimer **1a** at 25 °C in MCH (red line) and $CHCl_3$ (blue line) ([**1a**] = 2.6 × 10^{-5} M).

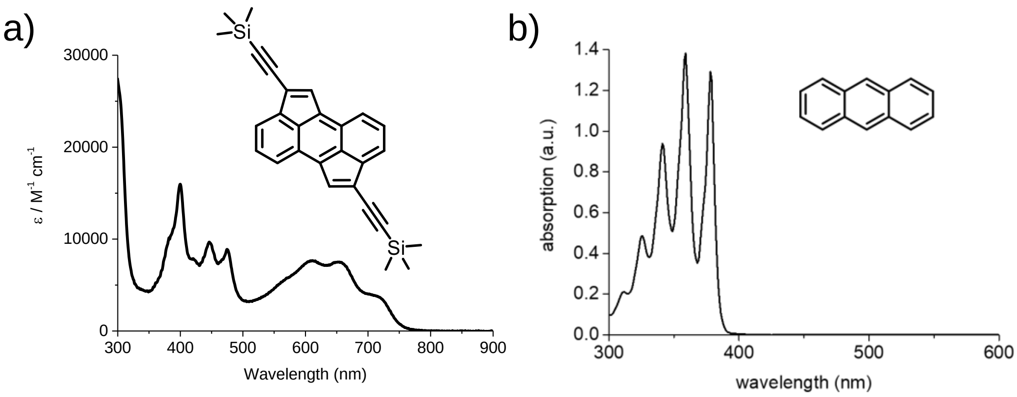

Figure S3.7. UV-vis absorption spectra (25 °C) of a) bis(trimethylsilylethynyl)-CPA and b) anthracene in toluene. Both molecular structures are shown in the inset.

***fs*-Transient Electronic Absorption Spectroscopy (*fs*-TEAS).** Transient electronic absorption spectroscopy of CPA dimers **1a** and **1b** in MCH and $CHCl_3$ and at room temperature were recorded. Photoexcitation at 730 nm of the two CPA dimers gives rise, independently of the solvent used, to a broad excited state absorption (ESA) band at around 1100-1150 nm, which has been assigned in previous works to the singlet excited state of CPA, with a reported lifetime for this singlet state of 8.9 ps.[11,12] Biexponential fitting of the kinetics of "bulky" CPA dimer **1b** in $CHCl_3$ (at 1148 nm) and MCH (at 1147 nm), and of CPA dimer **1a** in $CHCl_3$ (at 1136 nm) resulted in similar lifetimes (*i.e.*, 7.17, 9.19, and 8.20 ps, respectively). These results are consistent with the generation, upon photoexcitation, of a localized singlet excited state, similar

to that formed for a previously reported CPA monomer. However, when **1a** is dissolved in MCH, a significant increase of the lifetime of the excited species was observed (*i.e.*, 196 ps) as a consequence of the *inter*- and *intra*-molecular delocalization of the excited state prompted by the formation of supramolecular aggregates (note that the shape difference between the ESA bands of **1a** in $CHCl_3$ and MCH are mainly due to the difference in the ground state bleaching which is extended towards the NIR region in MCH).

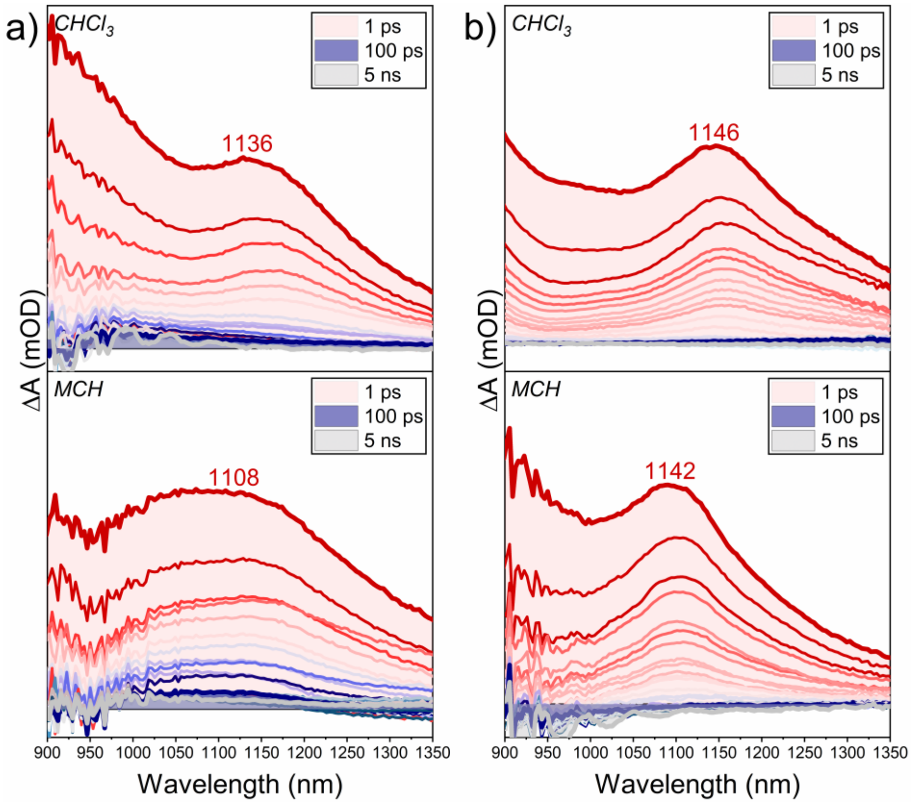

Figure S3.8. *fs*-Transient electronic absorption spectra in the NIR spectral region of CPA dimers a) **1a** and b) **1b** in $CHCl_3$ (top) and MCH (bottom) at room temperature in the full-time window (5 ns) (λ_{exc} = 730 nm).

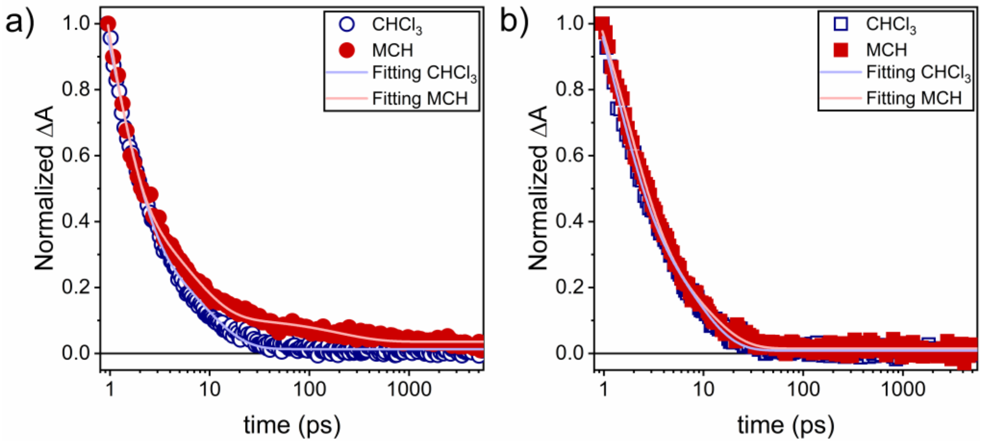

Figure S3.9. TEA kinetics of CPA dimers a) **1a** in $CHCl_3$ at 1136 nm (empty, blue circles) and MCH at 1102 nm (filled, red circles), and b) **1b** in $CHCl_3$ at 1148 nm (empty, blue circles) and MCH at 1147 nm (filled, red circles) together with the corresponding exponential fitting.

Table S1. Time constants (τ) and R square obtained through the exponential fitting of the TEA kinetics in Figure S3.9.

	CPA dimer **1a**		"bulky" CPA dimer **1b**	
	**$CHCl_3$**	**MCH**	**$CHCl_3$**	**MCH**
**τ_1**	0.984 ± 0.037 ps	0.702 ± 0.034 ps	1.099 ± 0.058 ps	1.512 ± 0.025 ps
**τ_2**	8.198 ± 0.346 ps	5.977 ± 0.031 ps	7.172 ± 0.272 ps	9.185 ± 0.493 ps
**τ_3**	—	196.354 ± 20.982 ps	—	—
**R^2**	0.99518	0.99453	0.99567	0.995651

Note: Triexponential fitting for **1a** and **1b** in $CHCl_3$ does not converge. Biexponential fitting of **1a** in MCH gives a $R^2 < 0.90$.

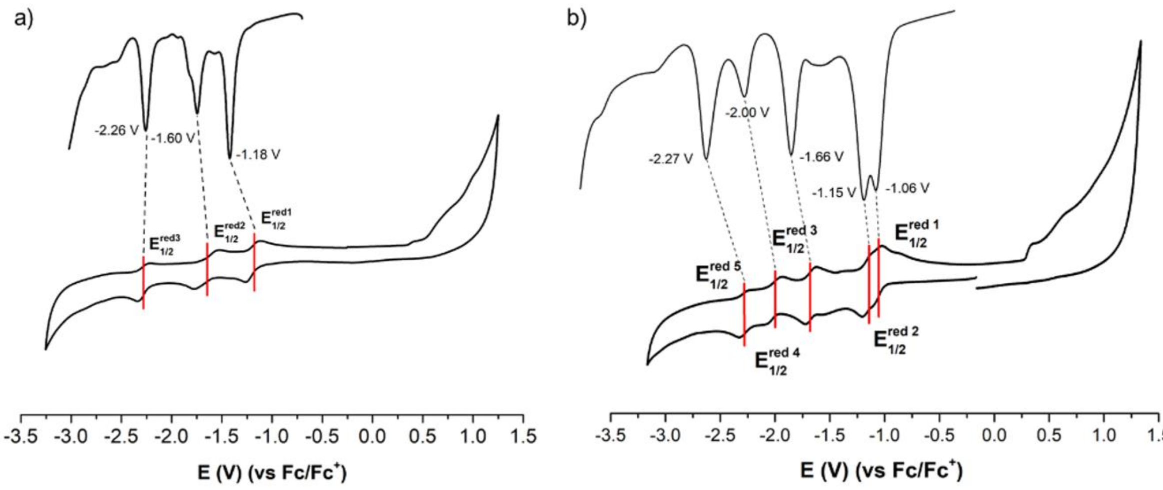

Figure S3.10. CV (bottom) and DPV (top) voltammograms of a) CPA monomer **4a** and CPA dimer **1a** at a scan rate of 0.1 V s^{-1} in a 0.1 M solution of *n*-Bu_4NPF_6 in THF. Potentials are referred to $E_{1/2}$ of the Fc^+/Fc redox couple.

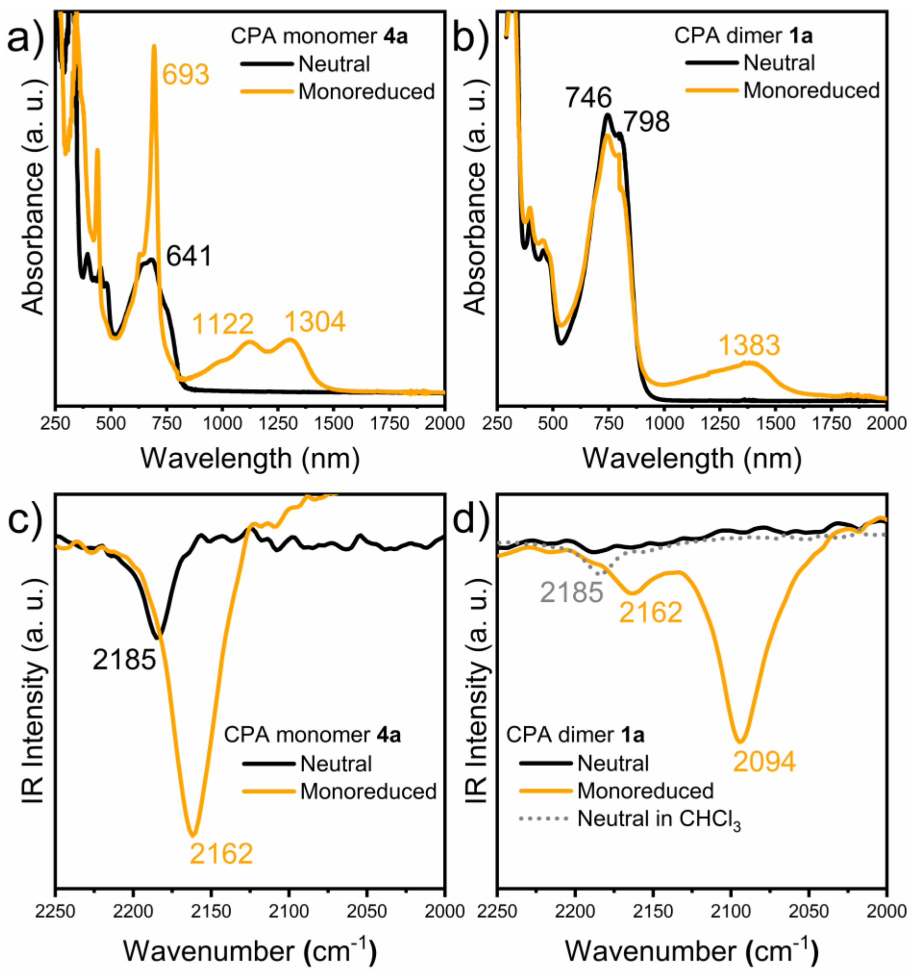

Figure S3.11. a,b) UV-vis-NIR electronic absorption and c,d) IR electronic absorption (in the ν(C≡C) region) spectra of a,c) CPA monomer **4a** and b,d) CPA dimer **1a** in Bu_4NPF_6 0.1M in CH_2Cl_2 at room temperature obtained upon electrochemical reduction; Color code: neutral species = black lines; first reduced species = orange lines. In d), the IR spectrum of the neutral CPA dimer **1a** in $CHCl_3$ has been depicted (dotted grey line) to better identify the position of the internal ν(C≡C) peak.

4. Nucleation-elongation model for cooperative supramolecular polymerizations

Assuming a two-state equilibrium, the degree of polymerization or the molar fraction of aggregated species $\alpha_{agg}(T)$ is related to temperature by means of a sigmoidal relation.

The model developed by ten Eikelder, Markvoort, and Meijer[16,17,18] extends nucleation-elongation based equilibrium models for growth of supramolecular homopolymers to the case of two monomer and aggregate types and can be applied to symmetric or non-symmetric supramolecular copolymerizations.

Considering a symmetric supramolecular polymerization, a cooperative supramolecular mechanism can be divided into nucleation phase (nucleus size of two) followed by an elongation phase. The values T_e, $\Delta H°_{nucl}$, $\Delta H°_e$, and $\Delta S°$ can be obtained from a non-linear least-square analysis of the experimental melting curves and the equilibrium constants associated to the nucleation and elongation phases as well as the cooperativity factor (σ) can be calculated using equations 1, 2 and 3:

$$\text{Nucleation step: } K_{nucl} = e^{\left(\frac{-(\Delta H_e^0 - \Delta H_{nucl}^o) - T\Delta S^o}{RT}\right)} \quad (1)$$

$$\text{Elongation step: } K_e = e^{\left(\frac{-(\Delta H_e^0 - T\Delta S^o)}{RT}\right)} \quad (2)$$

$$\sigma = \frac{K_{nucl}}{K_e} = e^{\left(\frac{\Delta H_{nucl}^o}{RT}\right)} \quad (3)$$

The cooling curves of CPA dimer **1a** in MCH monitored as 875 nm were fitted to a nucleation-elongation model. The fitting was performed on the cooling curves recorded for seven different concentrations ranging between 6.48×10^{-6} M and 7.77×10^{-5} M and in a temperature range from 90 ºC to 0 ºC (Figure S4.1), and the thermodynamic parameters of the cooperative self-assembly of CPA dimer **1a** in MCH determined (Table 1). A cooperative factor (σ) of 0.08 was derived for CPA dimer **1a**.

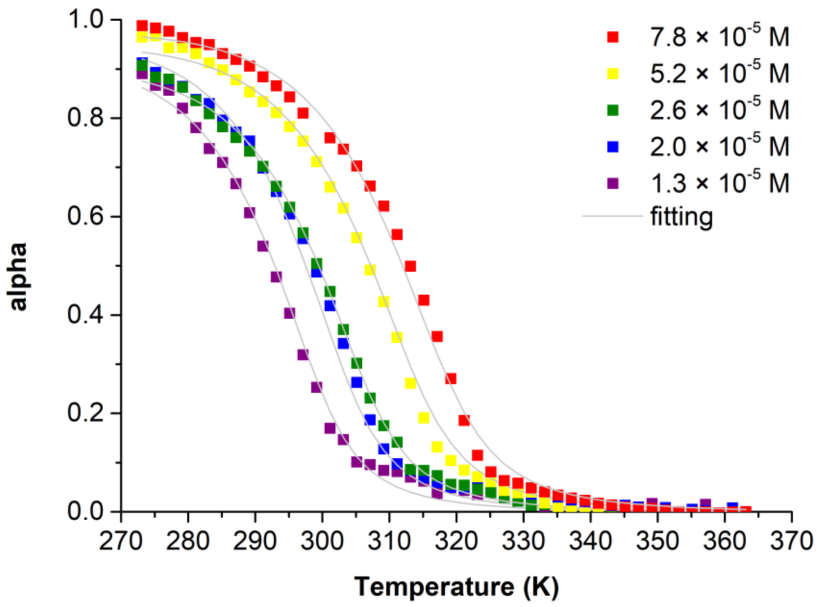

Figure S4.1. Cooling curves data (square dots) of CPA dimer **1a** in MCH monitored at 875 nm fitted to a nucleation-elongation model (solid lines). The fitting was performed for cooling curves recorded for five different concentrations ranging between 7.8 × 10^{-5} M and 1.3 × 10^{-5} M (see legend within the figure) in MCH and in a temperature range from 90 (363 K) to 0 ºC (273 K).

5. AFM studies on CPA dimers 1a and 1b

The propensity of **1a** to form aggregates was also investigated in some condensed phases using atomic force microscopy (AFM). In this context, drop-casting and spin-coating a MCH solution of **1a** on highly ordered pyrolytic graphite (HOPG) revealed the formation of µm-wide islands and µm-long fibers, respectively (Figures S5.1 and S5.2). The average height of such islands and fibers is in good agreement with that obtained by molecular modelling of a supramolecular arrangement of **1a** on HOPG (Figure S7.2).

On the contrary, AFM experiments carried out using a MCH solution of "bulky" CPA dimer **1b** on HOPG did not show any sign of fibers or islands formation (Figure S5.3). This finding supports, once again, the importance of supramolecular interactions (*i.e.*, CPA π-stacking and alkoxy van der Waals interactions) in promoting the organization of **1a** not only in solution but also in condensed phases.

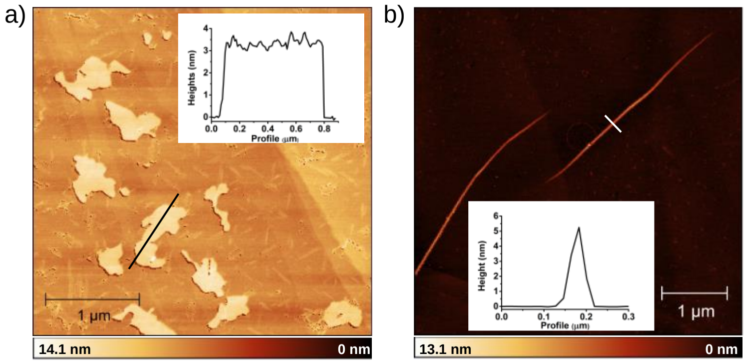

Figure S5.1. AFM topographic images of CPA dimer **1a** a) drop-casted and b) spin-coated on HOPG ([**1a**]: a) = 1 × 10^{-5} M, b) = 1 × 10^{-5} M, both in MCH). The inset in a,b) shows the AFM topographic profile along the indicated lines in the respective images.

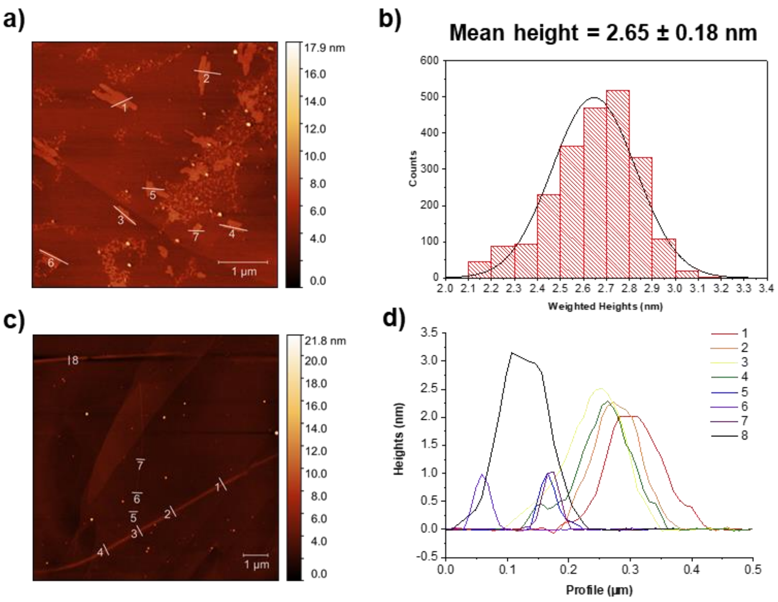

Figure S5.2. a) AFM image of CPA dimer **1a** drop-casted (20 µL of a 1 × 10^{-5} M solution of **1a** in MCH) on HOPG; b) Statistical height distribution of the islands in a) along the indicated white lines; c) AFM image of CPA dimer **1a** (1 × 10^{-5} M solution of **1a** in MCH) spin-coated (500 r.p.m) on HOPG; d) Height profiles of the fibres shown in c) along the indicated white lines.

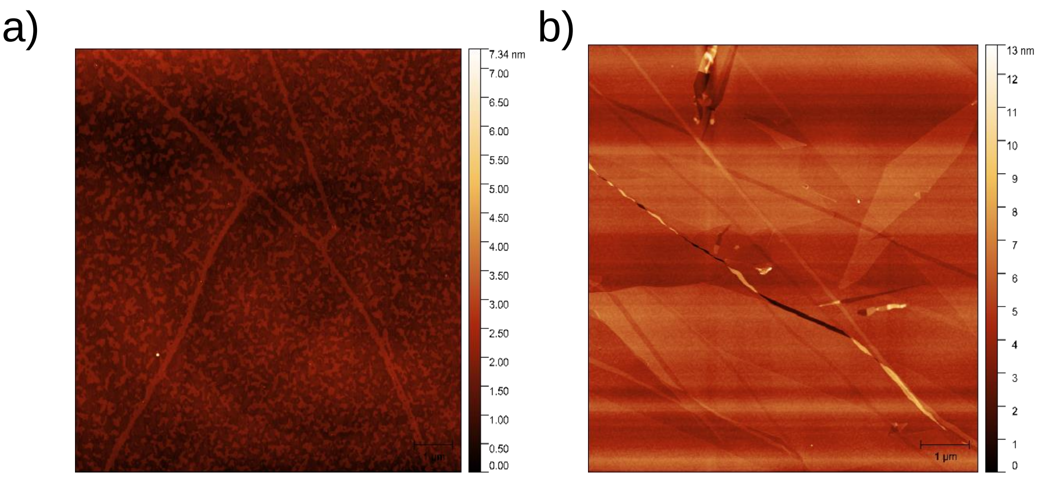

Figure S5.3. a) AFM image of "bulky" CPA dimer **1b** drop-casted on HOPG (10 µL of a 1 × 10^{-5} M solution of **1b** in MCH); b) AFM image of CPA dimer **1b** (100 µL of a 1 × 10^{-5} M solution of **1b** in MCH) spin-coated (2000 r.p.m.) on HOPG.

6. VT ^{1}H-NMR analysis on CPA dimers 1a and 1b

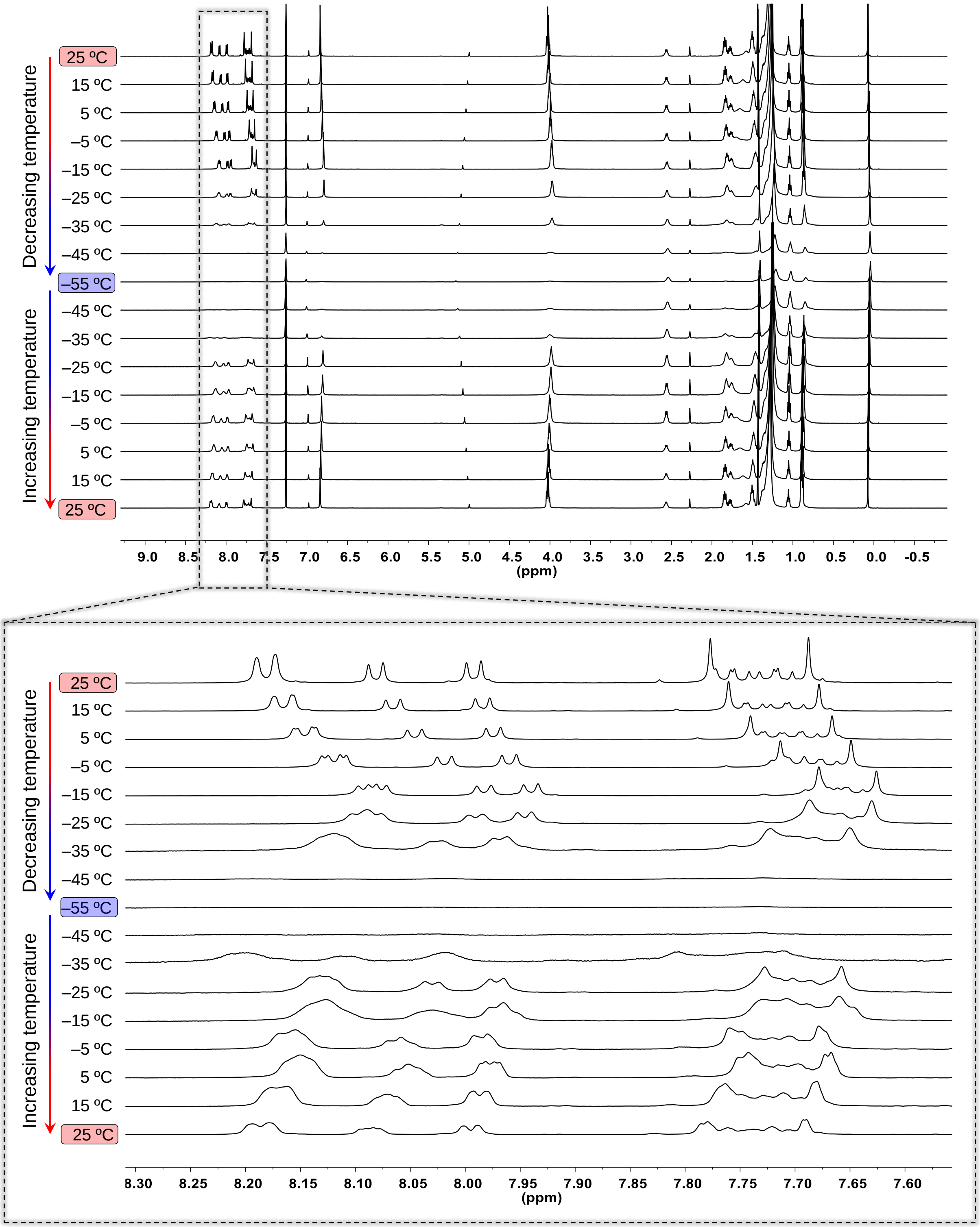

Figure S6.1. VT ^{1}H-NMR spectra of CPA dimer **1a** ([**1a**] = 5 × 10^{-3} M) in $CHCl_3$ recorded from 25 (top) to –55 ºC (middle spectra) and back to 25 ºC (bottom). Intermediate spectra recorded at 10 ºC increments; Cooling and heating rate: 2 ºC/min.

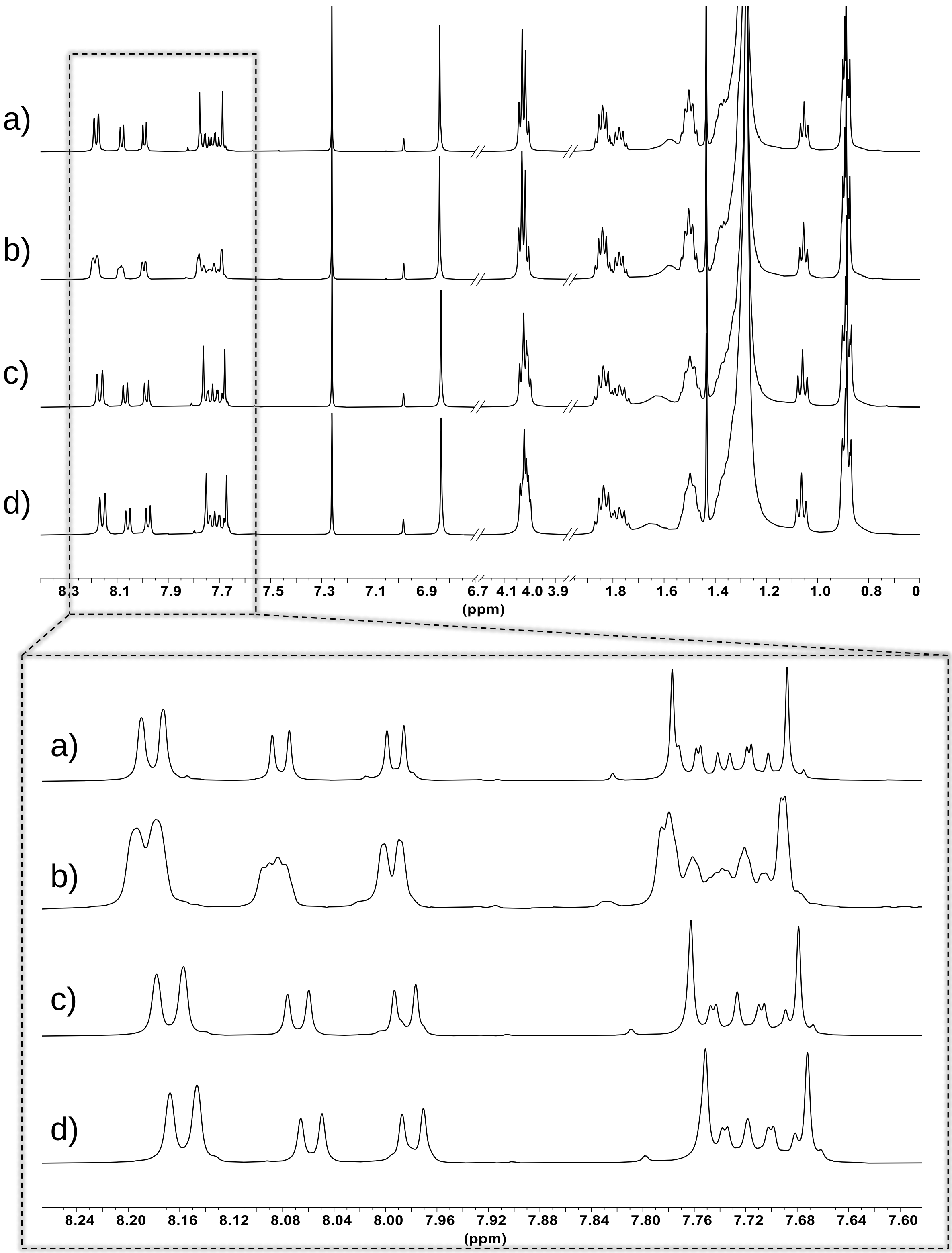

Figure S6.2. ^{1}H-NMR spectra of CPA dimer **1a** ([**1a**] = 5 × 10^{-3} M) in $CHCl_3$ recorded (a) at 25 ºC, (b) at 25 ºC after having cooled down the NMR tube used to record the spectrum in (a) to –55 ºC (cooling and heating rate: 2 ºC/min), (c) at 25 ºC upon leaving the NMR tube used to record the spectrum in (b)

for 2 days at 25 ºC, and (d) at 25 ºC after freezing the NMR tube used to record the spectrum in (c) down to –195.8 ºC using in liquid nitrogen and then heating the frozen solution to 25 ºC abruptly by submerging the NMR tube in a thermostatic bath kept at 25 ºC.

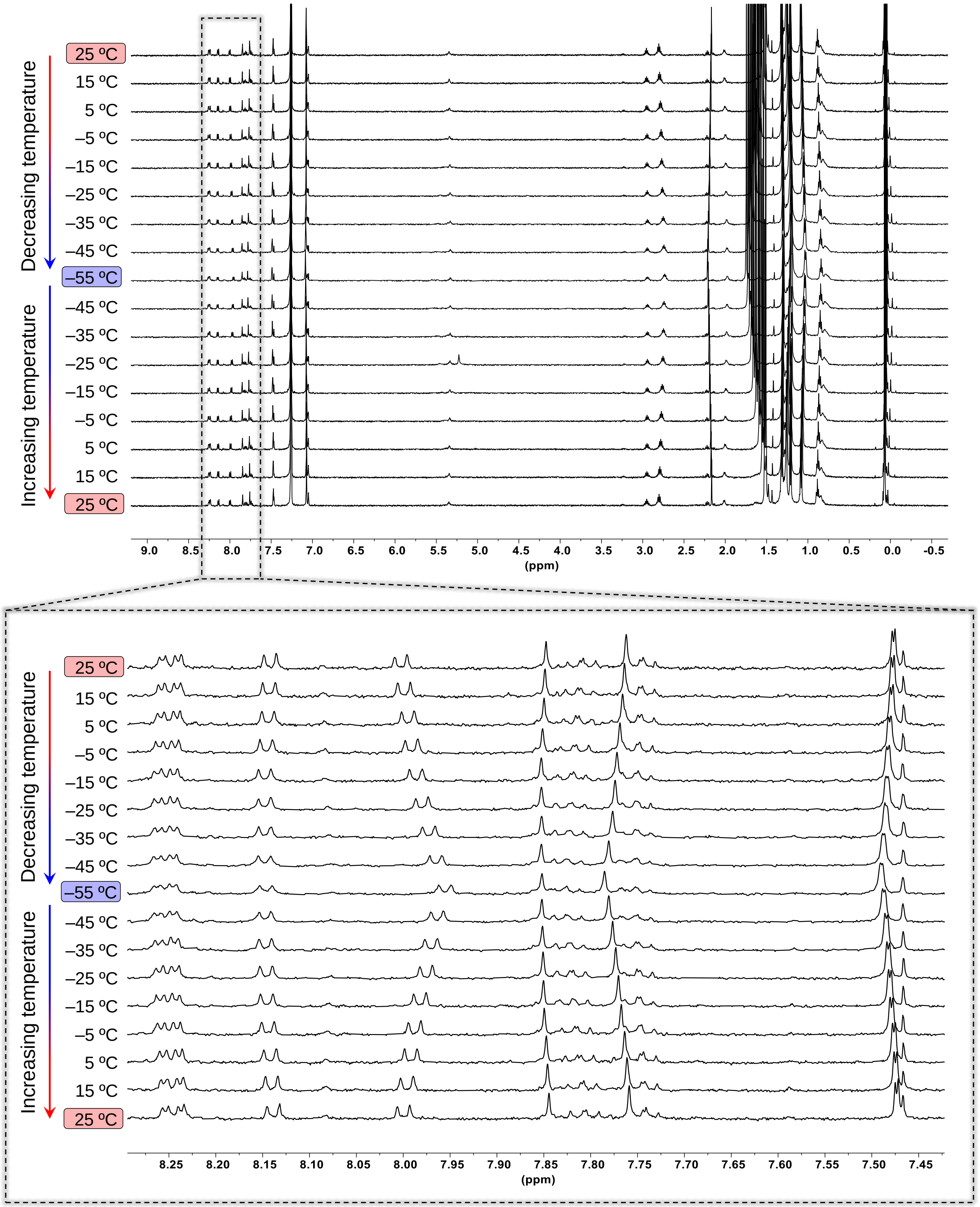

Figure S6.3. VT ^{1}H-NMR spectra of "bulky" CPA dimer **1b** ([**1b**] = 5 × 10^{-3} M) in $CHCl_3$ recorded from 25 (top) to –55 ºC (middle spectra) and back to 25 ºC (bottom). Intermediate spectra recorded at 10 ºC increments; Cooling and heating rate: 2 ºC/min.

7. Molecular modelling of CPA dimer 1a and possible organization arrangement on HOPG

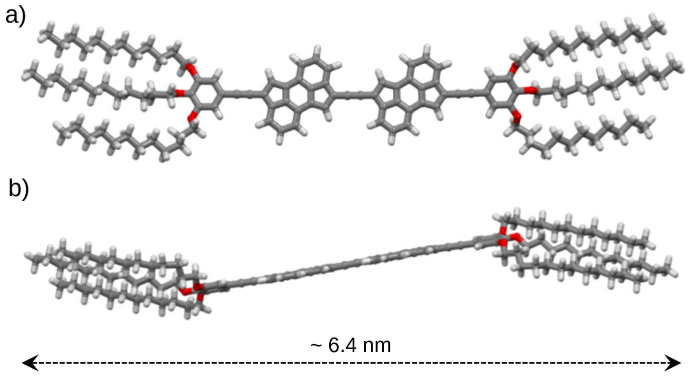

Figure S7.1. a) Top and b) lateral view (with respect to the CPA moieties) of the computer-simulated three-dimensional structure of CPA dimer **1a** obtained by using the HyperChem program with the MM+ force field. Carbon atoms are colored in light grey, hydrogen atoms in white and oxygen atoms in red. The end-to-end distance of the CPA dimer **1a** minimized structure is ~6.4 nm.

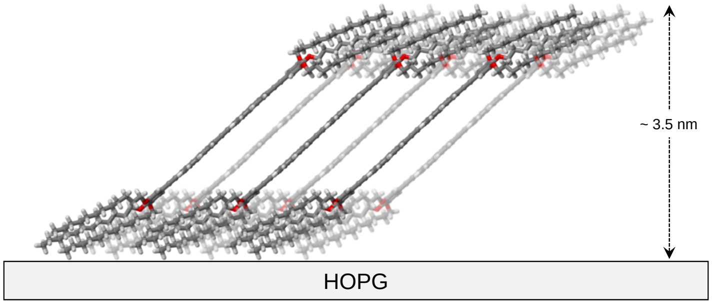

Figure S7.2. Lateral view (with respect to the CPA moieties) of a possible computer-simulated supramolecular arrangement of CPA dimer **1a** on HOPG giving rise to ~3.5 nm-height nanostructures (*i.e.*, the average height of the “nanoislands” formed upon drop-casting a MCH solution of CPA dimer **1a** ([**1a**] = 1 × 10^{-5} M) on HOPG, see Figure 3a and S5.1a). For an easier spatial visualization of the arrangement adopted by **1a**, the color of some CPA dimers have been faded.

8. Experimental and calculated Raman spectra of CPA dimers 1a and 1b, and CPA monomer 4a

Vibrational Raman studies on CPA monomer **4a** and CPA dimers **1a** and **1b** were performed to shed light on the chemical structure changes upon aggregation. Since the aggregation process must take place between planar or *quasi*-planar CPA dimer structures, the Raman fingerprint of the acetylenic bridge between the CPA units is a proper indicator of structural changes related to this phenomenon. The stretching vibration of the acetylene bonds, $\nu_{(C\equiv C)}$, emerge between 2200 cm^{-1} and 2100 cm^{-1}. In this spectral region, two different $\nu_{(C\equiv C)}$ bands are observed for the CPA dimers:

i) The higher energy $\nu_{(C\equiv C)}$ band is associated to the external acetylene groups, $\nu_{(C\equiv C)external}$. This band remains almost invariant with the aggregation phenomenon.
ii) The lower energy band is related to the acetylene bridge between the CPA cores, $\nu_{(C\equiv C)central}$. The planarity of CPA dimers is given by the dihedral angle between the CPA units (Θ), that can be directly related with the spectral changes of $\nu_{(C\equiv C)central}$.

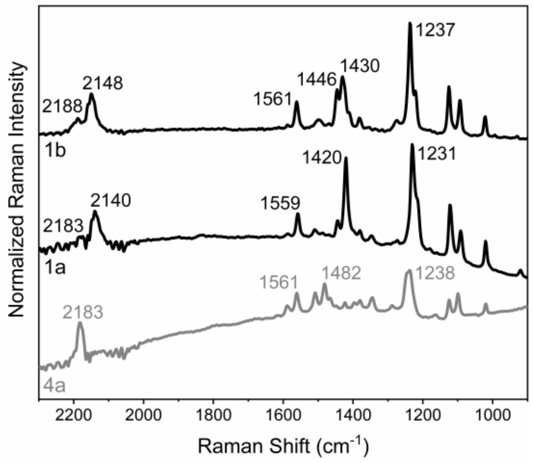

Figure S8.1. Full-range FT-Raman spectra of CPA monomer **4a** (grey line, bottom) and CPA dimers **1a** (black line, middle) and **1b** (black line, top) in the solid state at 25 ºC.

The contribution of several conformers with different values of Θ at room temperature is evident in the detection of structured Raman $\nu_{(C\equiv C)}$ bands. These contributions were resolved by deconvolution of the experimental FT-Raman in the desired spectral region using the Non-Linear Fitting tool in OriginPro 2018, with a Lorentzian function and the second derivative Peak Finding Method. Down-shifted $\nu_{(C\equiv C)central}$ correspond to more planar dimers (Θ close to 180º) since in these conformers the larger π-electron delocalization provokes the weakening of the acetylene bond and, hence, the diminution of its stretching force constant.

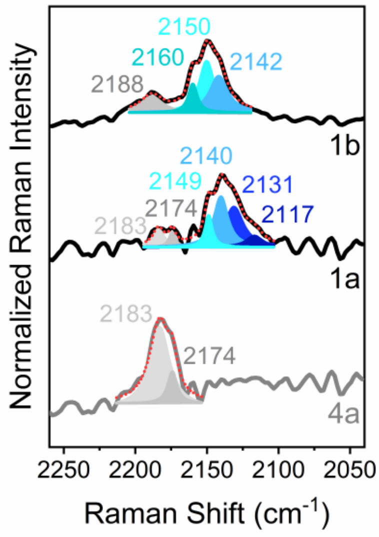

Figure S8.2. Lorentzian fitting of the Raman bands in the $\nu_{(C\equiv C)}$ region of the FT-Raman spectra of CPA monomer **4a** (grey line, bottom) and CPA dimers **1a** (black line, middle) and **1b** (black line, top) in solid state at 25 ºC.

Table S2. Results of the Lorentzian fitting of the Raman bands in the $\nu_{(C\equiv C)}$ region of the FT-Raman spectrum of CPA monomer **4a** in the solid state at 25 ºC.

**Model**	Lorentz	
**Equation**	y = y0 + (2*A/pi)*(w/(4*(x-xc)^2 + w^2))	
**Plot**	**Peak 1**	**Peak 2**
**y0**	0.00125 ± 2.58873E-4	0.00125 ± 2.58873E-4
**xc**	2183.16399 ± 0.84739	2174.16351 ± 0.74281
**w**	16.52752 ± 2.24816	7.65006 ± 2.94448
**A**	0.2058 ± 0.04094	0.03731 ± 0.0227
**Reduced Chi-Sqr**	2.24568×10^{-7}	
**R-Square (COD)**	0.97605	
**Adj. R-Square**	0.9703	

Table S3. Results of the Lorentzian fitting of the Raman bands in the $\nu_{(C\equiv C)}$ region of the FT-Raman spectrum of CPA dimer **1a** in the solid state at 25 ºC.

**Model**	Lorentz					
**Equation**	y = y0 + (2*A/pi)*(w/(4*(x-xc)^2 + w^2))					
**Plot**	**Peak 1**	**Peak 2**	**Peak 3**	**Peak 4**	**Peak 5**	**Peak 6**
**y0**	0.0165 ± 0	0.0165 ± 0	0.0165 ± 0	0.0165 ± 0	0.0165 ± 0	0.0165 ± 0
**xc**	2183.89221 ± 1.09011	2174.19311 ± 0.86416	2148.89261 ± 0.58098	2140.27372 ± 0	2131.20495 ± 1.52863	2116.8917 ± 2.97048

**w**	9.38233 ± 3.61068	5.40381 ± 3.22397	6.22181 ± 2.56843	11.71825 ± 5.12334	15.60202 ± 6.44311	13.51123 ± 9.69465
**A**	0.02568 ± 0.00882	0.01243 ± 0.00696	0.03036 ± 0.01867	0.09078 ± 0.06502	0.09611 ± 0.06636	0.02344 ± 0.0252
**Reduced Chi-Sqr**	1.91274×10^{-7}					
**R-Square (COD)**	0.97126					
**Adj. R-Square**	0.95642					

Table S4. Results of the Lorentzian fitting of the Raman bands in the $\nu_{(C\equiv C)}$ region of the FT-Raman spectrum of CPA dimer **1b** in the solid state at 25 ºC.

**Model**	Lorentz			
**Equation**	y = y0 + (2*A/pi)*(w/(4*(x-xc)^2 + w^2))			
**Plot**	**Peak 1**	**Peak 2**	**Peak 3**	**Peak 4**
**y0**	0.02962 ± 0	0.02962 ± 0	0.02962 ± 0	0.02962 ± 0
**xc**	2188.60773 ± 0.36454	2159.93839 ± 0.21535	2150.26461 ±0.30862	2141.78112±0.69373
**w**	18.82042 ± 1.25219	7.50014 ± 0.83918	12.26572 ± 1.70094	14.93304 ± 1.08753
**A**	0.04909 ± 0.0025	0.03318 ± 0.00584	0.09672 ± 0.02281	0.08393 ± 0.01781
**Reduced Chi-Sqr**	1.45042×10^{-8}			
**R-Square (COD)**	0.99733			
**Adj. R-Square**	0.99645			

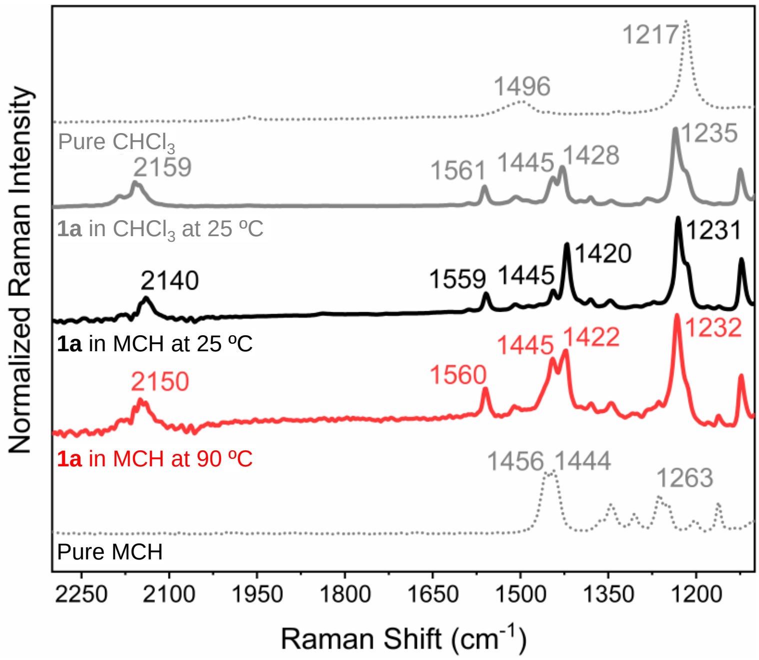

Figure S8.3. Full-range FT-Raman spectra of CPA dimer **1a** at 1 × 10^{-3} M in MCH at 90 ºC (red line) and 25 ºC (black line) and in $CHCl_3$ at 25 ºC (grey line). FT-Raman spectra of $CHCl_3$ (top, grey dotted lines) and MCH (bottom, grey dotted lines) are also showed as reference.

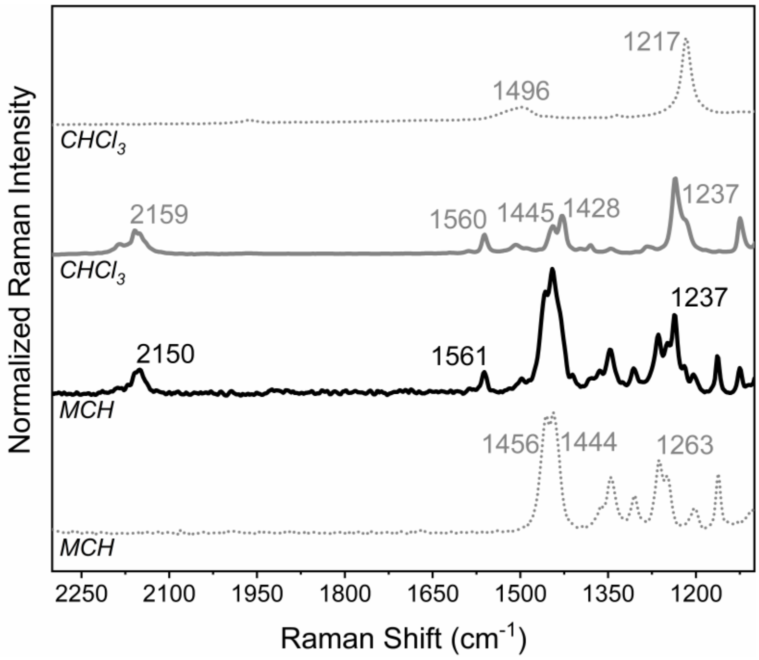

Figure S8.4. Full-range FT-Raman spectra of CPA dimer **1b** at 1 × 10^{-3} M at 25 ºC in MCH (black line) and in $CHCl_3$ (grey line). FT-Raman spectra of $CHCl_3$ (top, grey dotted lines) and MCH (bottom, grey dotted lines) are also showed as reference.

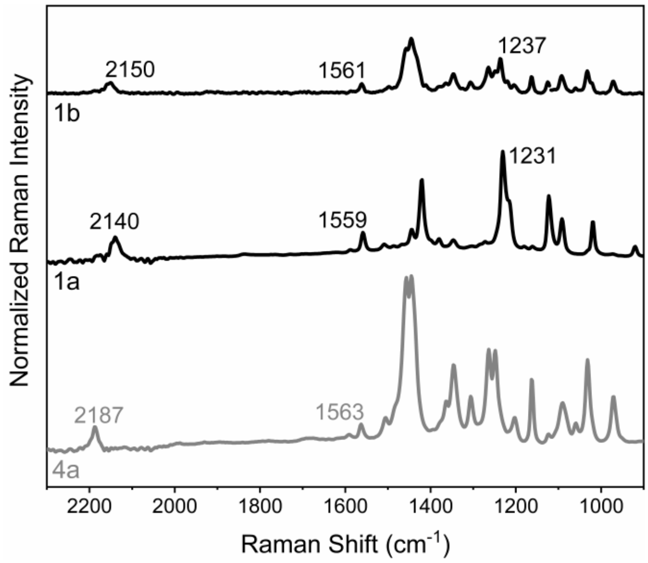

Figure S8.5. Full- range FT-Raman spectra of CPA dimers **1b** (top) and **1a** (middle) and CPA monomer **4a** (bottom) at 1 × 10^{-3} M in MCH at 25 ºC.

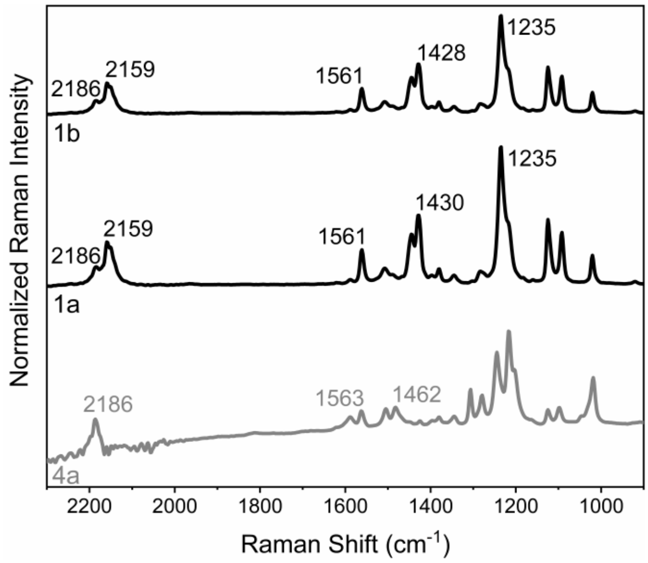

Figure S8.6. Full-range FT-Raman spectra of CPA dimers **1b** (top) and **1a** (middle), and CPA monomer **4a** (bottom) at 1 × 10^{-3} M in $CHCl_3$ (**1a** and **1b**) and tetrachloroethane (**4a**) at 25 ºC.

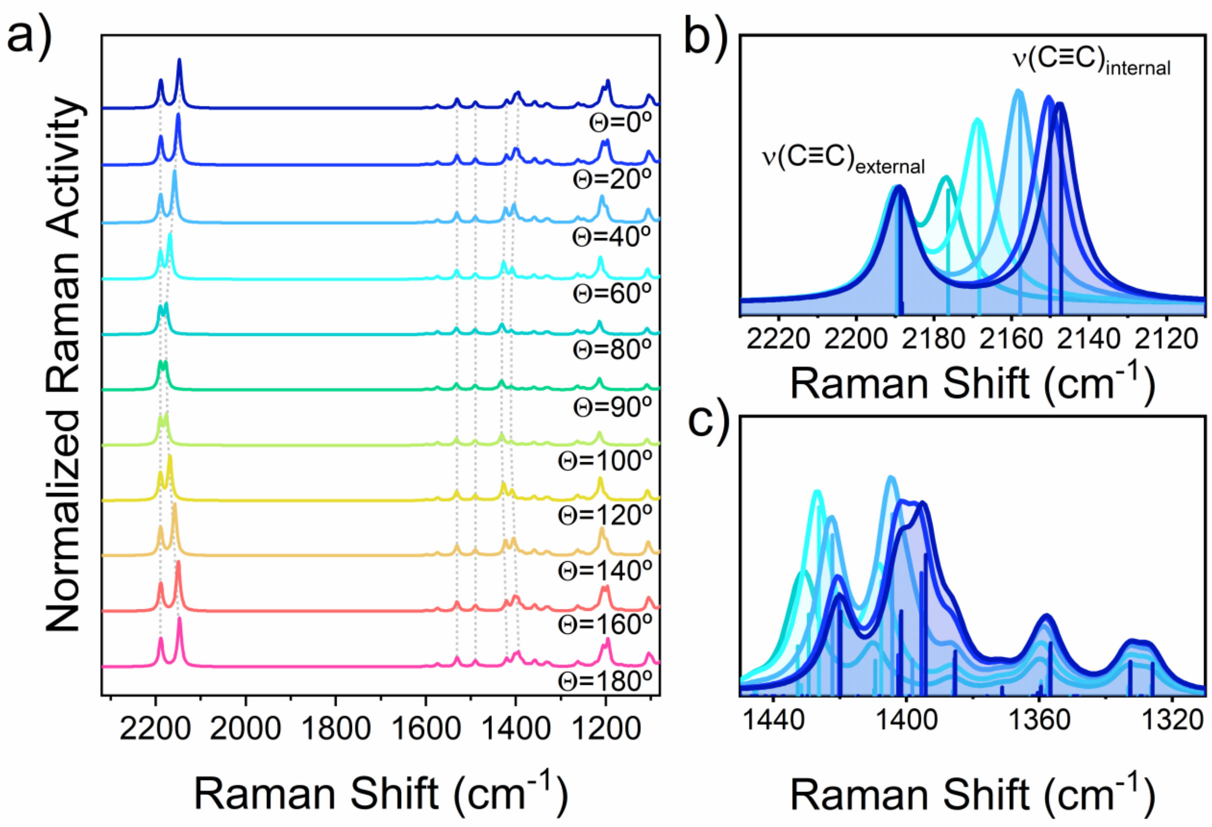

Figure S8.7. a) Theoretical full Raman spectra of CPA dimer **1a** varying the dihedral angle between the two CPA units (thereafter referred as Θ) from 0º (top, *anti*-coplanar conformation, Figure S1.2) to 180º (bottom, *sin*-coplanar conformation, Figure S1.2); b) zoom of the $\nu_{(C\equiv C)}$ region in a) scanning Θ from 0º to 90º (same color code to the one used in a), and c) zoom of the $\nu_{(C=C/C-C)}$ region in a) scanning Θ from 0º to 90º (same color code to the one used in a), calculated at the B3LYP/6-31G** level of theory and scaled down uniformly by a factor of 0.96. The conformer with Θ = 0º corresponds to the optimized geometry.

Table S5. Eigenvectors of the $\nu_{(C\equiv C)}$ and $\nu_{(C=C/C-C)}$ Raman bands in Figure S8.7b,c) of **1a** with the dihedral angle between the two CPA units Θ = 0º (right column) (optimized geometry, *anti*-coplanar conformation, Figure S1.2) and 40º (right column).

	**Θ = 0º**	**Θ = 40º**
**$\nu_{(C\equiv C)}$ region**	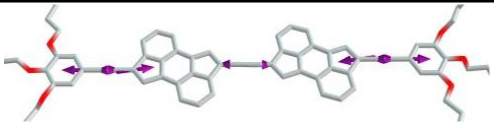 $\nu_{(C\equiv C)external}$ = 2189 cm^{-1}	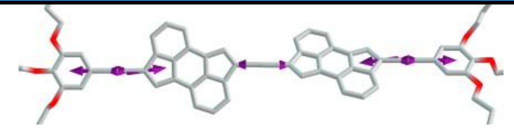 $\nu_{(C\equiv C)external}$ = 2189 cm^{-1}
	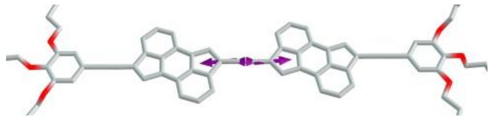 $\nu_{(C\equiv C)central}$ = 2147 cm^{-1}	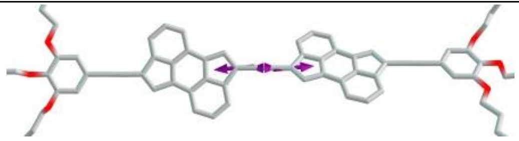 $\nu_{(C\equiv C)central}$ = 2158 cm^{-1}
**$\nu_{(C=C/C-C)}$ region**	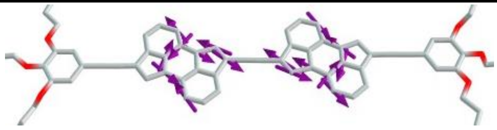 1420 cm^{-1}	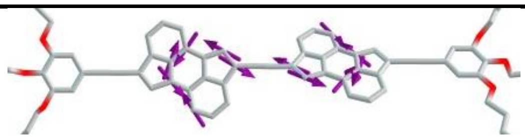 1422 cm^{-1}
	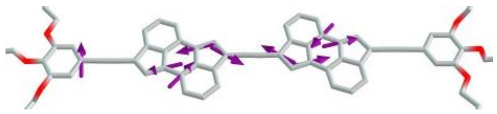 1402 cm^{-1}	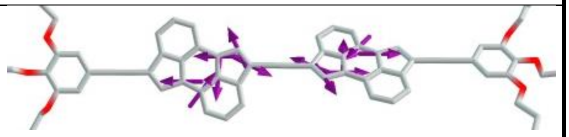 1402 cm^{-1}
	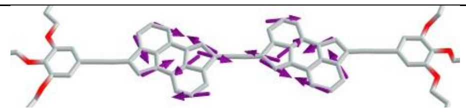 1394 cm^{-1}	

9. Calculated rotational energy barrier and Raman spectra of 1a and reference PAH dimers

To unravel if the contribution of a more "cumulenic" electronic structure of CPA dimer **1a** approaching a close-to-planar conformation is something exclusive of this system, quantum chemical calculations were performed on CPA dimer **1a** as well as on some reference dimers in which the two CPAs were replaced by phenyls (**Ph_2-1TB**) and hexabenzocoronenes (**HBC_2-1TB**) units (Figure S9.1). Calculations on "bulky" CPA dimer **1b** and CPA dimer **$1a_{6,6}$**, a regioisomer of **1a** in which the connection between the two CPA moieties takes place through their six-membered rings, were also carried out (Figure S9.1).

For these five PAH dimers (*i.e.*, **1a**, **1b**, **$1a_{6,6}$**, **HBC_2-1TB**, **Ph_2-1TB**), the variation of the energy barrier as a function of the dihedral angle (Θ) between the two bridged aromatic units (Figure S9.2a) as well as the corresponding theoretical vibrational Raman spectra for the different dimers' conformers were calculated (Figure S9.2b-f).

These studies showed that, besides CPA dimers **1a** and **1b**, only one of the other four systems (*i.e.*, pyracyl dimer **Py-1TB**) shows a significant variation (*i.e.*, 20 cm^{-1}) of the calculated $\nu_{(C\equiv C)central}$ Raman peak as a function of the Θ between the two PAH units, as also observed for CPA dimers **1a** and **1b** (*i.e.*, 30 and 31 cm^{-1} variation, respectively) (Figure S9.2b-f). For the other three calculated dimers having the acetylene bridge connecting two phenyl (*i.e.*, **Ph-1TB**), hexabenzocoronene (*i.e.*, **HBC-1TB**), or CPA units (*i.e.*, **$1a_{6,6}$**), no significant variation of the calculated $\nu_{(C\equiv C)central}$ Raman peak is seen upon varying the Θ between the two aromatic units (Figure S9.2b-f). This finding is particularly striking in the case **$1a_{6,6}$**, a regioisomer of **1a** having the two CPA units connected through their six member rings, which one may think would behave as its parent isomers **1a** and **1b**. A possible explanation of the observed trend can be found attending at the contribution of the "cumulenic" resonance form of the five dimers upon adopting a planar conformation. In this context, in **1a** and **1b** the number of Clar's sextets increases from two (in the "acetylenic" electronic configuration) to four (in the "cumulenic" resonance structure) (Figure S9.1). On the other hand, in the case of **Ph-1TB**, **HBC-1TB**, and **$1a_{6,6}$**, the number of Clar's sextets is reduced moving from the "acetylenic" to the "cumulenic" electronic structure in the case of **Ph-1TB** and **HBC-1TB** (*i.e.*, from two to zero, and from 14 to four, respectively), whereas it remains the same (*i.e.* two) in the case of **$1a_{6,6}$**.

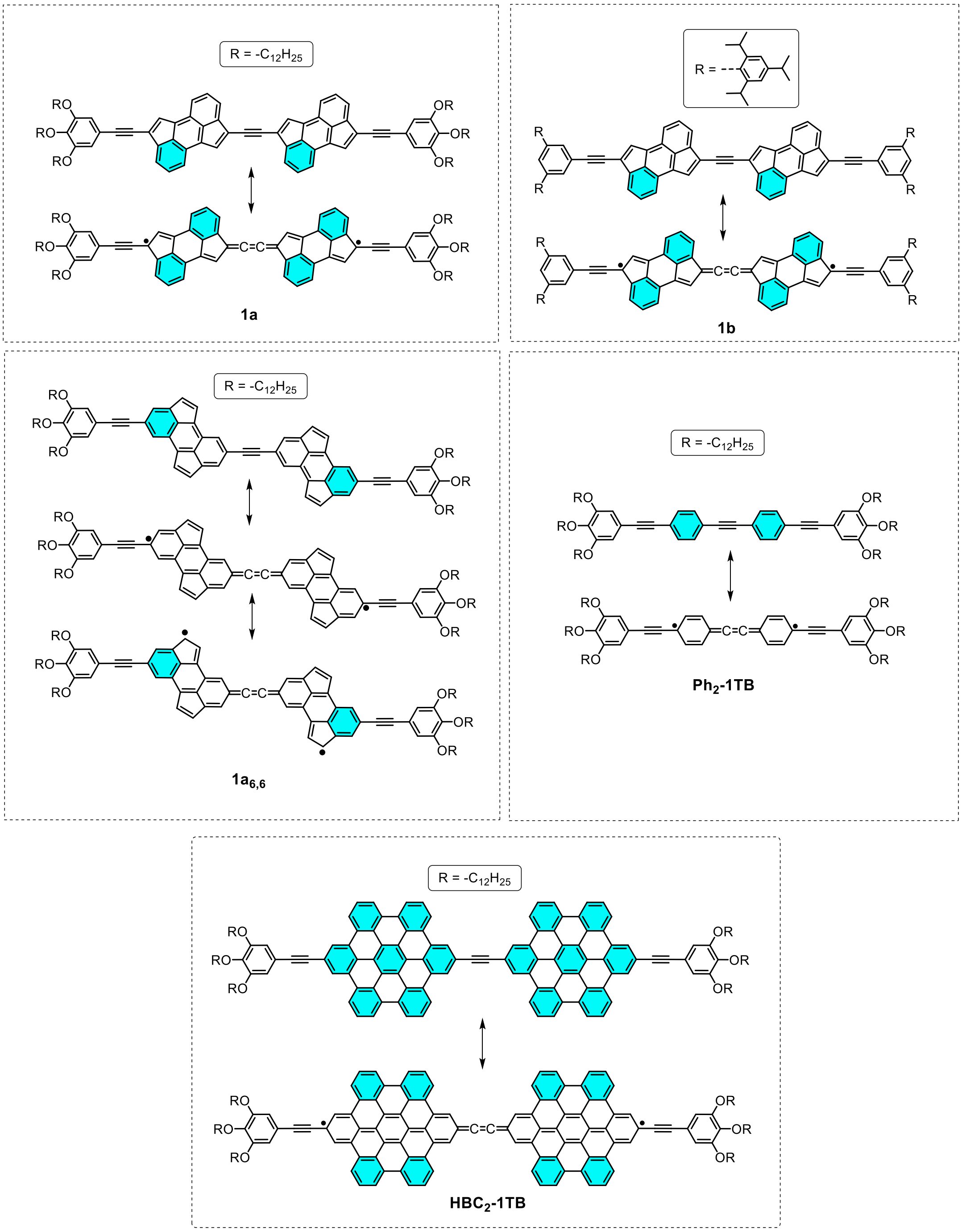

Figure S9.1. Representation of some canonical forms of the dimers **1a**, **1b**, **1a$_{6,6}$**, **HBC$_2$-1TB**, and **Ph$_2$-1TB**. Clar's sextets are coloured in cyano.

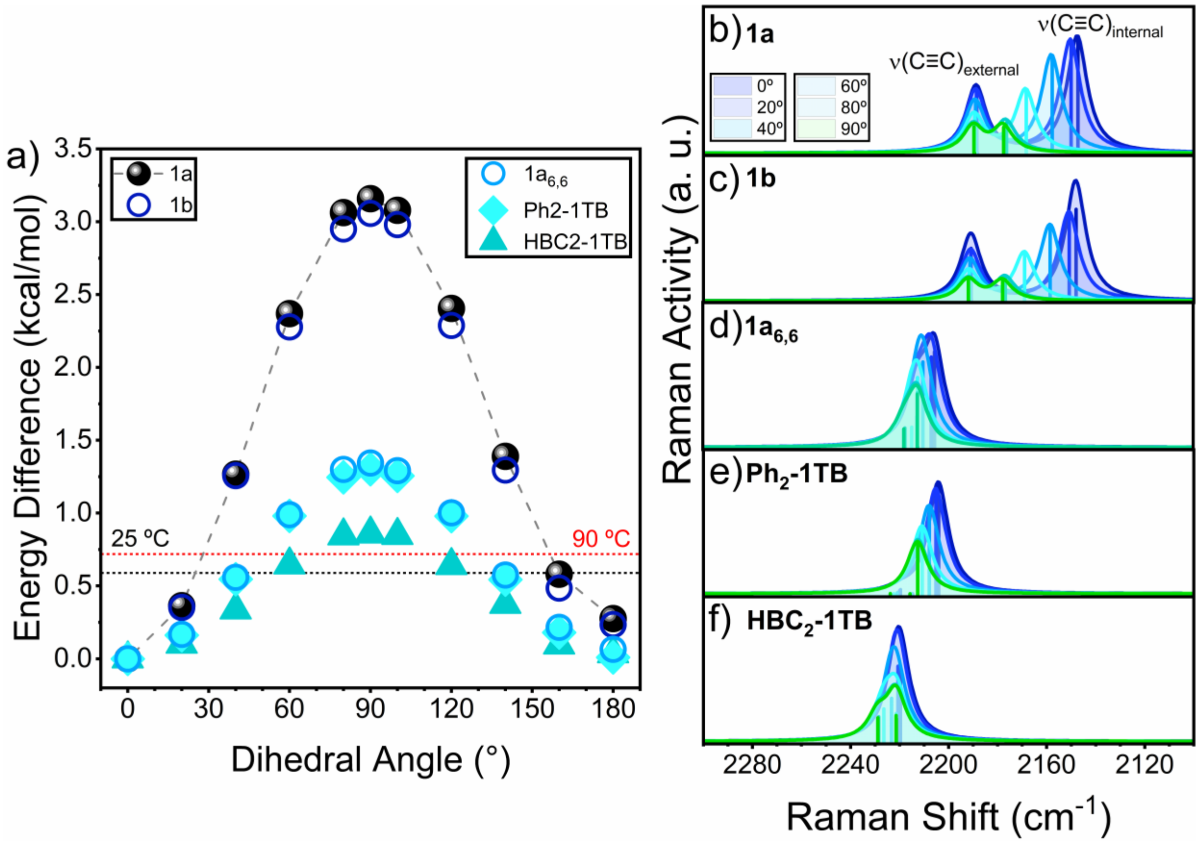

Figure S9.2. a) Calculated energy difference (kcal/mol) in the gas phase of the acetylene-bridged systems represented in Figure S9.1a (optimized geometry) as a function of the dihedral angle between the two π-conjugated PAH units (thereafter referred as Θ) (0º ≤ Θ ≤ 180º). The two horizontal lines indicates the thermal energy (E_T) of **1a** at 25 ºC (black dashed line) and 90 ºC (red dashed line). b-f) Calculated Raman spectra highlighting the variation of the $\nu_{(C\equiv C)central}$ and $\nu_{(C\equiv C)external}$ Raman peaks of PAH dimers represented in Figure S9.1a as a function of the Θ between the two π-conjugated moieties (0º ≤ Θ ≤ 180º). Calculations were performed at the B3LYP/6-31G** level of theory and theoretical Raman spectra were scaled down uniformly by a factor of 0.96.

10. Theoretical calculations on CPA dimer 1a and CPA monomer 4a

Theoretical calculations were used to pinpoint the origin of the low-energy optical transitions in **1a**. TD-DFT quantum chemical calculations at the B3LYP/6-31G** level on this CPA dimer predict an almost coplanar conformation of the two CPA units (Figure 7a) and assign its experimental NIR absorption to a HOMO→LUMO transition (*i.e.*, calculated value = 984 nm) (Figure S10.1 and Table S6). Looking at the HOMO and LUMO of **1a**, its FMOs are formed by symmetric combinations of the HOMO and LUMO of the acetylene spacer mixed with the antisymmetric combinations of the CPA units (Figure S10.2). Such situation in **1a** makes its HOMO and LUMO orbitals to be delocalized over the whole molecule leading to orbital topologies typical of alternant hydrocarbons. The destabilization and stabilization of the HOMO and LUMO of **1a**, respectively, with respect to its fragments (*i.e.*, larger for the LUMO given the two bonding couplings between the central acetylene and the two CPAs) produces a further reduction of the HOMO-LUMO gap compared to that of CPA monomer **4a** (Figure S10.3).

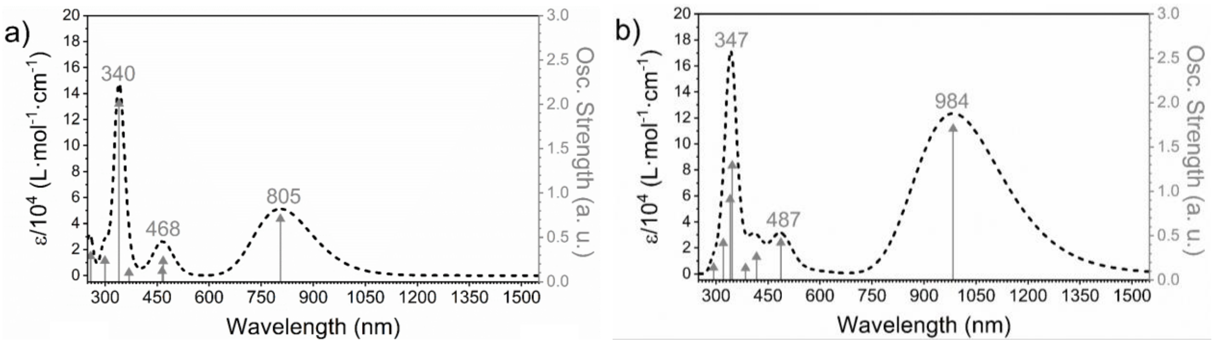

Figure S10.1. TD-DFT/B3LYP/6-31G** calculation in gas phase of a) CPA monomer **4a** and b) CPA dimer **1a**. Only transitions with oscillator strength (*f*) higher than 0.1 are shown for clarity.

Table S6. Main optical vertical transitions of CPA monomer **4a** and CPA dimer **1a** computed at the TD-DFT/B3LYP/6-31G** level of theory in in gas phase. H = HOMO, L = LUMO.

System	Wavelength (nm)	*f* (a. u.)	Main contribution
**4a**	805	0.7086	H → L (97%)
	468	0.2331	H–4 → L (45%) ; H–2 → L (36%) ; H–5 → L (13%)
	465	0.1192	H–2 → L (63%) ; H–4 → L (30%)
	340	2.0063	H → L+2 (94%)
**1a**	984	1.7036	H → L (97%)
	487	0.4217	H–6 → L (83%)
	417	0.2625	H–5 → L+1 (40%) ; H–7 → L+1 (35%)
	347	1.2899	H→ L+4 (67%) ; H–11 → L+1 (19%)

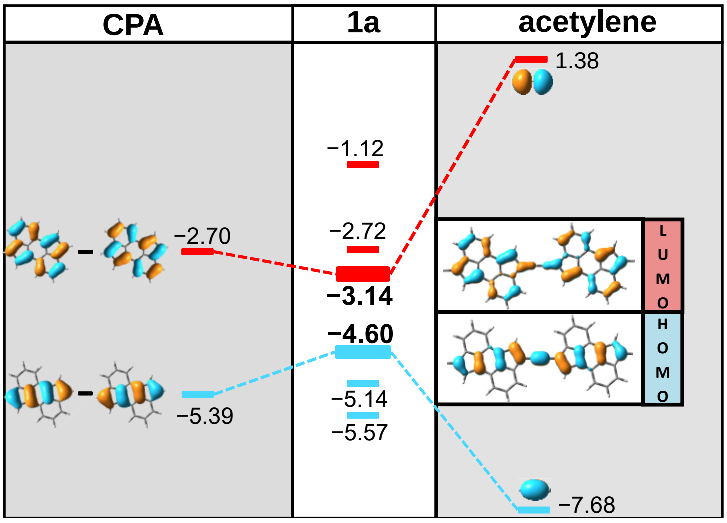

Figure S10.2. Evolution of the FMOs of CPA dimer **1a** (middle) from its CPA (left) and acetylene (right) components. HOMO and LUMO calculated at the B3LYP76-31G** level. The peripheral phenyl acetylene units have been omitted for clarity.

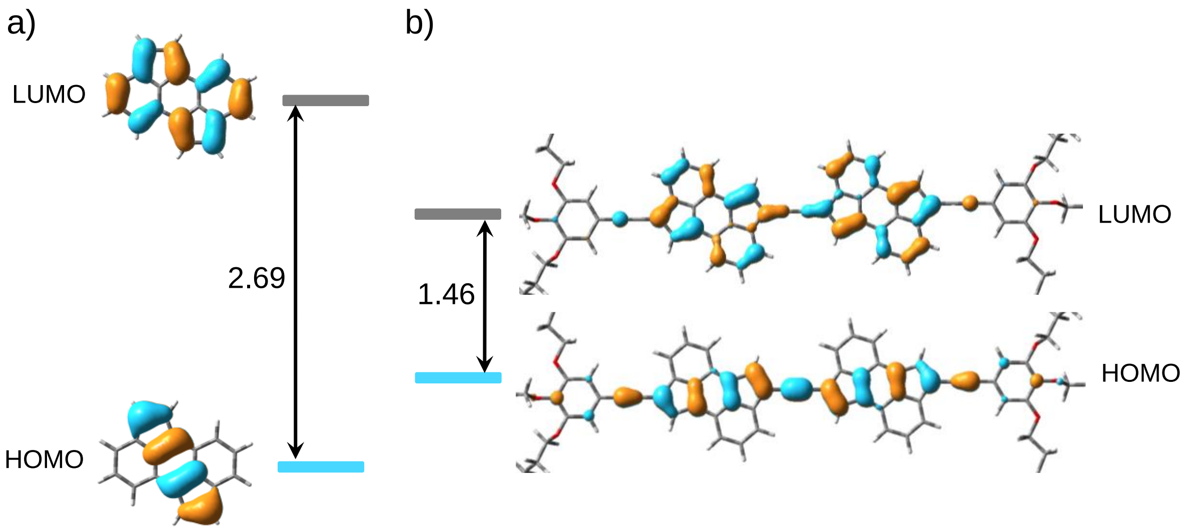

Figure S10.3. Wavefunction topologies and energies of the HOMO and LUMO of a) unsubstituted CPA and b) CPA dimer **1a** calculated at the B3LYP/6-31G** level (isovalue = 0.03). In the case of CPA dimer **1a**, part of the peripheral alkoxy chains have been omitted for clarity.

11. Optimized coordinates

CPA dimer 1a

C	-2.58661500	-2.51762300	0.46076900
C	-3.68412000	-3.41704500	0.62239400
C	-4.99774100	-3.00610800	0.51033200
C	-5.30430400	-1.64063500	0.22335400
C	-4.19908200	-0.77126600	0.06800500
C	-2.85140100	-1.19722700	0.18395100
C	-6.57586500	-1.02842200	0.07101400
C	-6.61051900	0.36098000	-0.21490100
C	-5.50665400	1.23085100	-0.37052000
C	-4.23349000	0.61888300	-0.21819700
C	-7.95960900	0.78536500	-0.32974800
C	-8.22478000	2.10558200	-0.60624500
C	-7.12796900	3.00594200	-0.76846400
C	-5.81387400	2.59598400	-0.65713000
C	-2.86600400	1.07318600	-0.28260000
C	-8.79927100	-0.41531100	-0.10053200
C	-7.94712100	-1.48288500	0.13596900
C	-10.20352700	-0.42853200	-0.12570100
C	-11.42357700	-0.41246400	-0.15220400
C	-12.84403500	-0.41425300	-0.17800300
C	-13.55669800	-1.60665300	0.03196200
C	-14.95102400	-1.59900400	0.01370900
C	-15.65518600	-0.40883800	-0.22219700
C	-14.94047200	0.79073600	-0.44103000
C	-13.54659500	0.78685900	-0.42009100
C	8.22437500	-2.10785800	0.60483000
C	7.12767300	-3.00835400	0.76704700
C	5.81352900	-2.59857400	0.65564700
C	5.50613900	-1.23349300	0.36896600
C	6.60989700	-0.36348500	0.21334600
C	7.95904300	-0.78768800	0.32826700
C	4.23289900	-0.62169700	0.21657700
C	4.19832300	0.76843700	-0.06968900
C	5.30343700	1.63794000	-0.22505000
C	6.57507200	1.02589800	-0.07263800
C	2.85059500	1.19422400	-0.18568100

C	2.58564600	2.51457300	-0.46256300
C	3.68303800	3.41412500	-0.62420800
C	4.99671000	3.00336000	-0.51209700
C	7.94626800	1.48053900	-0.13755500
C	2.00971900	-0.00641900	0.04307400
C	2.86546500	-1.07616900	0.28096800
C	0.61186000	-0.01904800	0.01583200
C	-0.61252200	0.01585100	-0.01754400
C	-2.01038200	0.00331800	-0.04477400
C	10.20280500	0.42656800	0.12426900
C	8.79855200	0.41308600	0.09902000
C	11.42285600	0.41081700	0.15085200
C	12.84331300	0.41308700	0.17676100
C	13.55555100	1.60572400	-0.03331300
C	14.94987500	1.59860500	-0.01494600
C	15.65446700	0.40873500	0.22118700
C	14.94017600	-0.79106500	0.44017600
C	13.54629800	-0.78772200	0.41909100
O	-15.62270000	-2.76423500	0.29021900
C	-24.23400300	-14.77517000	-3.30970400
C	-23.54184500	-13.50677900	-3.81747400
C	-22.78987400	-12.74503200	-2.71917100
C	-22.09359400	-11.47343900	-3.21881700
C	-21.34178500	-10.71232200	-2.12001000
C	-20.64538700	-9.44060400	-2.61968600
C	-19.89333200	-8.68036000	-1.52044500
C	-19.19666800	-7.40858500	-2.01964700
C	-18.44417800	-6.65030400	-0.91958100
C	-17.74759700	-5.37834500	-1.41899600
C	-16.99508100	-4.62858600	-0.31296800
C	-16.31615500	-3.36565100	-0.82017300
O	-17.02511400	-0.42158200	-0.27889600
C	-30.85898400	1.98375200	5.58156100
C	-29.34701300	2.03659300	5.82004300
C	-28.52548300	1.57962900	4.60830700
C	-27.01024800	1.62947500	4.83844900
C	-26.18904600	1.17285300	3.62640500
C	-24.67373100	1.22325200	3.85702800
C	-23.85249500	0.76732700	2.64474800

C	-22.33723500	0.81829200	2.87571000
C	-21.51652900	0.36348200	1.66286900
C	-20.00131700	0.41514200	1.89531800
C	-19.18756700	-0.03886200	0.67730000
C	-17.68812500	0.01846300	0.92258500
O	-15.71071600	1.89573600	-0.65177200
C	-18.66594500	16.52802300	-3.52645300
C	-17.58556500	15.45958100	-3.33431100
C	-18.15586700	14.06495000	-3.04836700
C	-17.08067600	12.98913300	-2.85346000
C	-17.65130300	11.59465300	-2.56763500
C	-16.57542900	10.51974500	-2.37018400
C	-17.14613600	9.12526000	-2.08456700
C	-16.06973600	8.05160600	-1.88306800
C	-16.64126500	6.65753700	-1.59800300
C	-15.56349600	5.58601900	-1.39104200
C	-16.14392000	4.19497800	-1.10822300
C	-15.06297800	3.14489900	-0.89603900
O	17.02438400	0.42205000	0.27799600
C	30.85999700	-1.98168200	-5.57898900
C	29.34808300	-2.03506500	-5.81771800
C	28.52620500	-1.57783600	-4.60631800
C	27.01102500	-1.62822300	-4.83670800
C	26.18947400	-1.17133700	-3.62500000
C	24.67421600	-1.22227500	-3.85587100
C	23.85263000	-0.76609300	-2.64392400
C	22.33742600	-0.81759200	-2.87513800
C	21.51637000	-0.36253600	-1.66262700
C	20.00121500	-0.41472300	-1.89533000
C	19.18711200	0.03951700	-0.67763700
C	17.68773000	-0.01833300	-0.92317400
O	15.62113100	2.76405100	-0.29156800
C	24.22969500	14.77725200	3.30746400
C	23.53786100	13.50870800	3.81529300
C	22.78591300	12.74682800	2.71706600
C	22.08995900	11.47508100	3.21677400
C	21.33819000	10.71381300	2.11804300
C	20.64212000	9.44194400	2.61778900
C	19.89014700	8.68151200	1.51862100

C	19.19381000	7.40959100	2.01790700
C	18.44146900	6.65106500	0.91790700
C	17.74520600	5.37897400	1.41742800
C	16.99293600	4.62888500	0.31145600
C	16.31429400	3.36585300	0.81879600
O	15.71081500	-1.89572700	0.65126000
C	18.67438900	-16.52437400	3.53520100
C	17.59326700	-15.45702200	3.34117900
C	18.16264700	-14.06200700	3.05527000
C	17.08672300	-12.98725700	2.85853200
C	17.65645500	-11.59241000	2.57271100
C	16.57988500	-10.51850900	2.37357900
C	17.14975100	-9.12369100	2.08790800
C	16.07271600	-8.05094400	1.88497400
C	16.64348600	-6.65659300	1.59977300
C	15.56516500	-5.58584300	1.39170000
C	16.14494000	-4.19458100	1.10864300
C	15.06355800	-3.14509300	0.89577300
H	-1.56791700	-2.88233900	0.55723000
H	-3.47187500	-4.45938500	0.84104800
H	-5.80232500	-3.72357400	0.64091000
H	-9.24374200	2.46983600	-0.70208200
H	-7.34093700	4.04824200	-0.98688200
H	-5.00996300	3.31420100	-0.78795100
H	-2.54149000	2.08589100	-0.48366500
H	-8.27152500	-2.49549100	0.33751500
H	-13.03778600	-2.53794200	0.22474300
H	-12.98349000	1.69587800	-0.58376300
H	9.24337900	-2.47198000	0.70071800
H	7.34077100	-4.05061700	0.98551700
H	5.00970700	-3.31689000	0.78647000
H	1.56690300	2.87914900	-0.55906500
H	3.47066800	4.45643000	-0.84291600
H	5.80120600	3.72092200	-0.64268600
H	8.27055100	2.49317900	-0.33912700
H	2.54107700	-2.08890600	0.48207500
H	13.03630400	2.53678900	-0.22627300
H	12.98352900	-1.69693200	0.58286000
H	-24.76104000	-15.29524200	-4.11628100

H	-23.50999900	-15.47660200	-2.87955100
H	-24.96792100	-14.54112600	-2.53007700
H	-24.28694700	-12.84082500	-4.27374200
H	-22.83971300	-13.76940300	-4.62051800
H	-22.04460000	-13.41189600	-2.26211800
H	-23.49256000	-12.48271500	-1.91513100
H	-22.83944400	-10.80742900	-3.67593500
H	-21.39149900	-11.73663000	-4.02292600
H	-20.59596700	-11.37833600	-1.66295700
H	-22.04383000	-10.44917700	-1.31587500
H	-21.39112400	-8.77424500	-3.07639200
H	-19.94343600	-9.70361300	-3.42392500
H	-19.14761000	-9.34676500	-1.06386700
H	-20.59521000	-8.41740800	-0.71612400
H	-19.94224900	-6.74147300	-2.47544500
H	-18.49501600	-7.67131600	-2.82420300
H	-17.69789300	-7.31648300	-0.46416300
H	-19.14507800	-6.38691800	-0.11486000
H	-18.49401300	-4.71087800	-1.87223800
H	-17.04790100	-5.64215600	-2.22465200
H	-16.23578700	-5.28118400	0.13571200
H	-17.68700000	-4.35340400	0.49291600
H	-17.04836100	-2.65861100	-1.22516200
H	-15.59008000	-3.60343100	-1.61138300
H	-31.41636100	2.31559000	6.46360700
H	-31.15064300	2.62647000	4.74306700
H	-31.18749200	0.96531700	5.34475400
H	-29.09103000	1.41167400	6.68651500
H	-29.05449600	3.06066100	6.08942800
H	-28.78222100	2.20474000	3.74091200
H	-28.81869200	0.55484500	4.33836100
H	-26.75453800	1.00432100	5.70596300
H	-26.71819700	2.65434700	5.10893200
H	-26.44478600	1.79788900	2.75884400
H	-26.48079000	0.14793600	3.35598500
H	-24.41785400	0.59788300	4.72428300
H	-24.38206100	2.24811000	4.12784300
H	-24.10834800	1.39258900	1.77741000
H	-24.14375000	-0.25761400	2.37399700

H	-22.08111700	0.19238700	3.74246400
H	-22.04604500	1.84313600	3.14705000
H	-21.77121200	0.98930400	0.79591400
H	-21.80627600	-0.66145200	1.39140700
H	-19.74677800	-0.21206100	2.76131800
H	-19.71221700	1.44008800	2.16762500
H	-19.42254300	0.59060400	-0.18979600
H	-19.46119300	-1.06580500	0.40440000
H	-17.39840800	-0.63744100	1.75467700
H	-17.37038700	1.04144600	1.15936700
H	-18.22725100	17.51058900	-3.72858900
H	-19.32461100	16.27914200	-4.36634600
H	-19.29376100	16.62193800	-2.63301800
H	-16.92171200	15.75435400	-2.51014900
H	-16.95215900	15.41407500	-4.23071600
H	-18.82022400	13.76982300	-3.87331900
H	-18.79086600	14.11083800	-2.15187800
H	-16.41693600	13.28530000	-2.02851600
H	-16.44582000	12.94434200	-3.74996500
H	-18.31368000	11.29754000	-3.39325500
H	-18.28735100	11.63968200	-1.67203300
H	-15.91349400	10.81675800	-1.54414800
H	-15.93894100	10.47470400	-3.26546000
H	-17.80590100	8.82673100	-2.91169700
H	-17.78450300	9.17065800	-1.19068100
H	-15.41069100	8.34979000	-1.05518200
H	-15.43075700	8.00607600	-2.77649000
H	-17.29666400	6.35620900	-2.42716600
H	-17.28229500	6.70254300	-0.70634300
H	-14.90958800	5.88721000	-0.56035500
H	-14.92182400	5.54156200	-2.28235000
H	-16.78341000	3.87572400	-1.93994200
H	-16.78153200	4.22743600	-0.21618300
H	-14.42414700	3.41049400	-0.04119800
H	-14.41921700	3.06351300	-1.78362500
H	31.41762800	-2.31372300	-6.46079700
H	31.18816400	-0.96305200	-5.34254700
H	31.15169400	-2.62396600	-4.74017600
H	29.05591400	-3.05933100	-6.08672800

H	29.09207200	-1.41058300	-6.68449600
H	28.81906500	-0.55285300	-4.33674900
H	28.78297200	-2.20250900	-3.73861700
H	26.71932300	-2.65329300	-5.10681600
H	26.75528500	-1.00350500	-5.70452800
H	26.48087100	-0.14622200	-3.35495300
H	26.44524300	-1.79593800	-2.75713300
H	24.38289400	-2.24733000	-4.12631800
H	24.41830900	-0.59733700	-4.72342800
H	24.14354000	0.25904300	-2.37354000
H	24.10850800	-1.39092700	-1.77628600
H	22.04658300	-1.84262900	-3.14612100
H	22.08128100	-0.19210700	-3.74218800
H	21.80577300	0.66258900	-1.39151800
H	21.77107500	-0.98794100	-0.79537700
H	19.71246100	-1.43985900	-2.16729300
H	19.74665100	0.21207000	-2.76162000
H	19.46039100	1.06664500	-0.40508600
H	19.42210900	-0.58954400	0.18974600
H	17.37032500	-1.04150100	-1.15958300
H	17.39798600	0.63714000	-1.75559700
H	24.75672200	15.29741700	4.11398800
H	24.96356800	14.54337500	2.52774400
H	23.50549400	15.47854200	2.87741100
H	22.83577300	13.77117000	4.61842800
H	24.28315600	12.84289800	4.27145500
H	23.48855400	12.48467200	1.91293500
H	22.04044400	13.41354600	2.26011800
H	21.38789700	11.73811200	4.02096500
H	22.83600100	10.80922400	3.67380000
H	22.04020400	10.45082400	1.31383000
H	20.59218000	11.37967100	1.66107600
H	19.94017700	9.70480400	3.42208400
H	21.38804500	8.77575800	3.07444000
H	20.59202300	8.41870200	0.71425100
H	19.14424000	9.34774000	1.06208600
H	18.49212300	7.67219100	2.82247600
H	19.93957000	6.74268500	2.47371200
H	19.14241400	6.38779400	0.11318800

H	17.69501400	7.31703500	0.46246400
H	17.04541100	5.64268600	2.22303000
H	18.49178100	4.71175400	1.87077000
H	17.68497200	4.35376000	-0.49434700
H	16.23351100	5.28123800	-0.13736000
H	15.58806400	3.60357500	1.60988300
H	17.04665200	2.65908700	1.22398600
H	18.23634700	-17.50724700	3.73725900
H	19.30341900	-16.61813900	2.64260500
H	19.33176300	-16.27447800	4.37580400
H	16.95869100	-15.41166300	4.23676400
H	16.93072400	-15.75281000	2.51632700
H	18.79879300	-14.10774300	2.15958800
H	18.82571700	-13.76588100	3.88089900
H	16.45074600	-12.94259500	3.75424800
H	16.42424700	-13.28442400	2.03293300
H	18.29354700	-11.63729400	1.67784500
H	18.31764900	-11.29434900	3.39893800
H	15.94240200	-10.47357100	3.26815200
H	15.91908600	-10.81647200	1.54697700
H	17.78898900	-9.16896200	1.19463900
H	17.80851000	-8.82429900	2.91552800
H	15.43293500	-8.00548000	2.77782600
H	15.41460800	-8.34999100	1.05665500
H	17.28515500	-6.70150300	0.70856800
H	17.29812000	-6.35451600	2.42926700
H	14.92294100	-5.54140500	2.28261200
H	14.91193400	-5.88777900	0.56075300
H	16.78288500	-4.22698900	0.21684000
H	16.78397600	-3.87473600	1.94048400
H	14.41951900	-3.06369600	1.78315600
H	14.42508100	-3.41126300	0.04084500

CPA dimer 1b

C	2.63647900	2.51824400	-0.11420300
C	3.75340100	3.40842400	-0.11161700
C	5.05794200	2.95581600	-0.10623100
C	5.33498500	1.55420300	-0.10333200
C	4.21117700	0.69518800	-0.10697400

C	2.87306900	1.16400800	-0.11180800
C	6.59273300	0.89640700	-0.09576900
C	6.59758700	-0.52209000	-0.09279600
C	5.47484400	-1.38208300	-0.09719800
C	4.21578500	-0.72481000	-0.10463800
C	7.93713600	-0.99002800	-0.08236300
C	8.17349900	-2.34429400	-0.07691500
C	7.05699600	-3.23519000	-0.08215200
C	5.75211100	-2.78334200	-0.09187700
C	2.83840600	-1.15399000	-0.10853900
C	8.80218600	0.21370700	-0.07894200
C	7.97418300	1.32513200	-0.08774200
C	10.20705700	0.19159800	-0.06456500
C	11.42592300	0.13843200	-0.04894500
C	12.84685300	0.09525300	-0.02630000
C	-8.18448500	2.36038200	-0.03966100
C	-7.06962200	3.25335100	-0.04179900
C	-5.76392500	2.80401000	-0.05737500
C	-5.48406600	1.40333000	-0.07213100
C	-6.60523600	0.54126100	-0.07036200
C	-7.94565400	1.00661200	-0.05406900
C	-4.22380500	0.74844500	-0.08707800
C	-4.21665400	-0.67150400	-0.09847300
C	-5.33886400	-1.53259400	-0.09731300
C	-6.59776100	-0.87717300	-0.08236200
C	-2.87776000	-1.13786300	-0.11020100
C	-2.63870700	-2.49161300	-0.12203600
C	-3.75398700	-3.38384400	-0.12199200
C	-5.05931700	-2.93365200	-0.11001800
C	-7.97834600	-1.30853100	-0.07408900
C	-2.01317400	0.06807400	-0.10556900
C	-2.84717100	1.18013000	-0.09225300
C	-0.61514500	0.04758100	-0.11084000
C	0.60833300	-0.01818800	-0.11302400
C	2.00634700	-0.04040100	-0.11228100
C	-10.21333200	-0.18014400	-0.04042100
C	-8.80842900	-0.19875400	-0.05656800
C	-11.43237200	-0.13139700	-0.02411600
C	-12.85350600	-0.09519600	-0.00270400

H	1.62581200	2.91640600	-0.11776800
H	3.56362100	4.47765100	-0.11363700
H	5.87779300	3.66782600	-0.10394900
H	9.18406400	-2.74269600	-0.06845800
H	7.24755100	-4.30427100	-0.07800900
H	4.93267000	-3.49583600	-0.09516600
H	2.49224800	-2.17936400	-0.10743500
H	8.32067500	2.35044400	-0.08702300
H	-9.19575200	2.75687800	-0.02657100
H	-7.26213600	4.32203000	-0.03053300
H	-4.94582200	3.51804700	-0.05814100
H	-1.62732200	-2.88785800	-0.13112200
H	-3.56227900	-4.45268700	-0.13145000
H	-5.87788300	-3.64714300	-0.11005100
H	-8.32294100	-2.33446800	-0.07936100
H	-2.50285700	2.20610400	-0.08568800
C	14.99346800	1.26606600	-0.06629900
C	14.91605800	-1.20303200	0.09266800
C	-14.93002900	1.19114700	0.12142600
C	-14.99396000	-1.27703100	-0.05403700
C	15.63650300	-2.52170300	0.30233600
C	16.33498800	-3.16350900	-0.76847200
C	15.66929700	-3.11680100	1.61285900
C	17.13166400	-4.28246300	-0.47447300
C	16.47919700	-4.24396700	1.79904500
C	17.24543000	-4.83123700	0.79485300
H	17.68344500	-4.74363200	-1.27631700
H	16.52447300	-4.69251700	2.77896900
C	15.80171500	2.53740600	-0.24702600
C	16.49726800	3.13563300	0.85037700
C	15.92417800	3.12598700	-1.55524600
C	17.37712700	4.19868600	0.58768800
C	16.81378600	4.19631600	-1.70987600
C	17.57752100	4.73431600	-0.67647200
H	17.92579100	4.62487800	1.41077800
H	16.92705000	4.63789100	-2.68745300
C	13.52227400	-1.14115500	0.00365600
H	12.94101400	-2.05443600	-0.02985900
C	13.59603600	1.28890700	-0.03061700

H	13.06950800	2.23524500	-0.01747300
C	15.63105000	0.01112100	0.02689100
H	16.71442300	-0.02131600	0.04855200
C	15.08891100	2.76854200	-2.83780400
C	14.94186400	1.26058600	-3.12839600
C	13.68587800	3.41735100	-2.72355000
C	15.72741400	3.35324400	-4.12760800
H	15.92049700	0.78466200	-3.22379700
H	14.37167800	0.72808300	-2.37428100
H	14.41702100	1.12955500	-4.08100800
H	13.76944000	4.49134300	-2.52785300
H	13.13560000	3.28597400	-3.66257000
H	13.08621700	2.97313900	-1.92923600
H	15.16178100	2.99122000	-4.99112100
H	15.69217700	4.44543200	-4.16785100
H	16.76682000	3.03369600	-4.25397700
C	16.38397000	2.72294600	2.35634000
C	14.92892600	2.44256000	2.79631600
C	17.29508500	1.50675500	2.64505400
C	16.86623700	3.85654200	3.30263500
H	14.29053900	3.30843500	2.58856500
H	14.48885900	1.57709900	2.31274900
H	14.90278600	2.26257900	3.87640200
H	18.33100100	1.73197200	2.36967800
H	17.27734900	1.26465000	3.71424200
H	16.98705500	0.61812900	2.09617000
H	16.64334300	3.56726800	4.33413500
H	17.94499800	4.02913400	3.25372500
H	16.35448600	4.80344300	3.10375500
C	18.56237400	5.88406100	-0.95972400
C	19.60515900	5.42097300	-2.00472300
C	17.78920500	7.10379300	-1.51496500
C	19.32025900	6.33316000	0.30394800
H	20.17598900	4.56335500	-1.63393700
H	19.13455300	5.12549100	-2.94691200
H	20.31121500	6.22994400	-2.22418100
H	17.04650100	7.45780900	-0.79253300
H	18.47844700	7.92929000	-1.72622700
H	17.26313200	6.86476800	-2.44383900

H	20.01483000	7.14041400	0.04983200
H	18.64162100	6.71386500	1.07418700
H	19.90738300	5.51732000	0.73824100
C	16.30919400	-2.74280300	-2.27630900
C	14.89235500	-2.37680500	-2.77436100
C	17.30141200	-1.58204300	-2.52280500
C	16.76297600	-3.90099100	-3.20661600
H	14.19696900	-3.20497600	-2.59804300
H	14.48354300	-1.48891400	-2.30414400
H	14.92132100	-2.19195300	-3.85355400
H	18.30951200	-1.86757100	-2.20361600
H	17.34402400	-1.33990300	-3.59126100
H	17.02254200	-0.67658600	-1.98613100
H	16.60221100	-3.59626400	-4.24519300
H	17.82644500	-4.13787300	-3.11239200
H	16.18752400	-4.81610200	-3.03478500
C	14.81160200	-2.70495600	2.86401000
C	14.74515500	-1.19007800	3.14780800
C	13.37686700	-3.26823500	2.69975900
C	15.36718200	-3.32292700	4.17661800
H	15.74662900	-0.77303600	3.27554700
H	14.23328400	-0.62684200	2.37448400
H	14.19741500	-1.02512800	4.08205200
H	13.40236100	-4.34592500	2.50812000
H	12.80184800	-3.10096400	3.61801800
H	12.83448900	-2.79070800	1.88368700
H	14.79567500	-2.92288900	5.01921800
H	15.26260500	-4.41059900	4.21800600
H	16.41951000	-3.06819100	4.33821300
C	18.13807600	-6.04529100	1.11297300
C	19.16447500	-5.65665300	2.20347200
C	17.26152600	-7.21149600	1.62836000
C	18.91681200	-6.54126000	-0.12017400
H	19.80700600	-4.83858500	1.86179200
H	18.67551000	-5.33260900	3.12672100
H	19.80439300	-6.51212100	2.44794100
H	16.52793700	-7.51252900	0.87331700
H	17.88390700	-8.08231100	1.86386500
H	16.71385000	-6.93984500	2.53555100

H	19.54460400	-7.39376600	0.15869700
H	18.24732900	-6.87408100	-0.92003200
H	19.57484800	-5.76560800	-0.52549400
C	-15.65078300	2.50873200	0.33704300
C	-15.67933900	3.10022800	1.64380600
C	-16.34311800	3.16145900	-0.73797600
C	-16.46957400	4.24727100	1.83350600
C	-17.11425800	4.29107700	-0.44197900
C	-17.21762200	4.84757000	0.82977300
H	-16.50465000	4.68903000	2.81434200
H	-17.66004200	4.76885800	-1.24119900
C	-15.79475100	-2.55140800	-0.24676600
C	-15.91023700	-3.13033100	-1.56009600
C	-16.49040000	-3.16212600	0.84359800
C	-16.79439200	-4.20348000	-1.72594400
C	-17.36462400	-4.22703100	0.56972000
C	-17.55887600	-4.75326000	-0.69927600
H	-16.90259600	-4.63787200	-2.70729800
H	-17.91364500	-4.66235000	1.38780500
C	-13.59648700	-1.29263000	-0.01665300
H	-13.06507000	-2.23627700	-0.00969300
C	-15.63849100	-0.02622800	0.04776300
H	-16.72197400	-0.00035700	0.07019200
C	-13.53563600	1.13731600	0.03445900
H	-12.95953400	2.05428000	0.01036000
C	-14.83930700	2.67128200	2.90175200
C	-14.76916900	1.15305600	3.16547500
C	-13.40439000	3.24144000	2.76557400
C	-15.41826800	3.26698700	4.21525600
H	-15.77004900	0.72759200	3.26632700
H	-14.23662600	0.60382100	2.39612400
H	-14.24028600	0.97889400	4.10881700
H	-13.43026000	4.32201200	2.59093600
H	-12.84032900	3.06084900	3.68814500
H	-12.85105300	2.77838500	1.94808500
H	-14.86545900	2.84852000	5.06138800
H	-15.31056400	4.35303200	4.28074900
H	-16.47474300	3.01414100	4.35042300
C	-16.32528400	2.73614400	-2.24446000

C	-14.90682700	2.38515300	-2.74921500
C	-17.30695700	1.56455800	-2.48176400
C	-16.79904800	3.88444800	-3.17623100
H	-14.22216800	3.22457900	-2.58444600
H	-14.48258900	1.50733700	-2.27345200
H	-14.93998100	2.19050800	-3.82659600
H	-18.31502900	1.83954300	-2.15334800
H	-17.35670100	1.32281100	-3.54995300
H	-17.01403100	0.66157400	-1.94911800
H	-16.65147900	3.57254700	-4.21460800
H	-17.86272200	4.11484300	-3.06754900
H	-16.22671900	4.80437200	-3.02041900
C	-18.10829700	6.08192700	1.06477500
C	-18.09509200	6.54668700	2.53338300
C	-17.61152300	7.25438200	0.18588000
C	-19.56830200	5.74020700	0.68327000
H	-18.45609800	5.76654500	3.21143200
H	-17.09316000	6.84567000	2.85855700
H	-18.75094300	7.41544000	2.65073600
H	-17.64048300	7.00661900	-0.87916000
H	-18.23968500	8.13934700	0.33881900
H	-16.58029400	7.52238300	0.43812600
H	-20.21690100	6.61031900	0.83597900
H	-19.65587900	5.44023100	-0.36506200
H	-19.95114700	4.91904700	1.29804100
C	-15.07238500	-2.75936900	-2.83722800
C	-14.93276500	-1.24869900	-3.11717200
C	-13.66622700	-3.40143500	-2.72281000
C	-15.70303400	-3.33862900	-4.13335200
H	-15.91379200	-0.77839400	-3.21550000
H	-14.37055400	-0.71728500	-2.35635200
H	-14.40309500	-1.10840700	-4.06579300
H	-13.74466100	-4.47699900	-2.53374400
H	-13.11413500	-3.26159900	-3.65953700
H	-13.07097300	-2.95879600	-1.92435500
H	-15.13635800	-2.96737300	-4.99225100
H	-15.66145700	-4.43029700	-4.18105100
H	-16.74375800	-3.02404300	-4.26128700
C	-16.38365100	-2.76181500	2.35332100

C	-14.93246300	-2.47085500	2.79889600
C	-17.30756400	-1.55737600	2.65049100
C	-16.85627400	-3.90876400	3.28844200
H	-14.28494600	-3.32831200	2.58459200
H	-14.50018500	-1.59664000	2.32429100
H	-14.91040300	-2.30059000	3.88063700
H	-18.34111900	-1.79184800	2.37397700
H	-17.29191300	-1.32177400	3.72115800
H	-17.00950400	-0.66205800	2.10688400
H	-16.63698900	-3.62732100	4.32288100
H	-17.93330200	-4.09103400	3.23699300
H	-16.33554700	-4.84888200	3.08088100
C	-18.53752900	-5.90518500	-0.99482400
C	-19.57946200	-5.43832900	-2.03900000
C	-17.75712100	-7.11671200	-1.55784400
C	-19.29688600	-6.36818500	0.26293500
H	-20.15530300	-4.58641500	-1.66285400
H	-19.10752800	-5.13296100	-2.97736700
H	-20.28114300	-6.24870900	-2.26712500
H	-17.01491500	-7.47330900	-0.83616900
H	-18.44193800	-7.94357100	-1.77798100
H	-17.22943600	-6.86758500	-2.48314000
H	-19.98684100	-7.17664600	0.00024000
H	-18.61861100	-6.75191500	1.03199200
H	-19.88915700	-5.55873100	0.70216500

CPA monomer 4a

C	2.84595300	-2.25837700	0.29981600
C	1.77126300	-3.17290300	0.07507500
C	0.44887400	-2.77672300	0.09223800
C	0.10839900	-1.41118700	0.34056800
C	1.19129800	-0.52736300	0.55969600
C	2.54964800	-0.93833600	0.54127000
C	-1.17659800	-0.81202400	0.39879800
C	-1.24491400	0.57862400	0.66423100
C	-0.16192300	1.46254900	0.88353000
C	1.12297600	0.86348100	0.82534900
C	-2.60331400	0.98981800	0.68316000

C	-2.89928800	2.30989200	0.92521700
C	-1.82455900	3.22429400	1.14992300
C	-0.50218500	2.82799600	1.13232800
C	2.48359800	1.33418700	0.97739800
C	-3.41371400	-0.22241100	0.41311500
C	-2.53735000	-1.28257600	0.24742500
C	4.76323600	0.30231600	0.88001800
C	3.36004200	0.27414800	0.81187800
C	5.98226400	0.30071500	0.93411100
C	7.40165900	0.31584900	0.99989600
C	8.08812100	1.50989200	1.25396100
C	9.48553500	1.53334900	1.31003900
C	10.22093400	0.34963800	1.11497100
C	9.52112500	-0.85945600	0.87171200
C	8.13158100	-0.87658800	0.80174800
C	-4.81706300	-0.25059300	0.34586300
C	-6.03616000	-0.24679600	0.29316600
C	-7.45567600	-0.26197800	0.22932600
C	-8.14362300	-1.46290500	-0.01191900
C	-9.53644600	-1.46786000	-0.07931400
C	-10.26434500	-0.28248700	0.10100200
C	-9.57512500	0.92588200	0.34950100
C	-8.18250500	0.93473100	0.41435700
O	11.58314700	0.35008500	1.28260800
C	25.94154000	-2.62329600	-2.76696200
C	24.49919200	-2.42456800	-3.24221600
C	23.51906400	-2.13159500	-2.09963200
C	22.07218900	-1.93168700	-2.56652600
C	21.09269600	-1.63774500	-1.42369100
C	19.64557300	-1.43894400	-1.89084500
C	18.66676000	-1.14266100	-0.74803400
C	17.21934500	-0.94551800	-1.21504300
C	16.24214700	-0.64552300	-0.07205300
C	14.79437700	-0.45063300	-0.53960300
C	13.82483800	-0.14614600	0.60897400
C	12.39219800	0.03533000	0.13145800
O	10.03982600	2.73931600	1.63182000
C	17.97905000	15.37104500	-1.30417500
C	17.90888800	13.84755700	-1.44483200

C	16.64903500	13.23788000	-0.81786200
C	16.57071300	11.71256900	-0.95441600
C	15.31059700	11.10306100	-0.32788800
C	15.23270300	9.57768000	-0.46578200
C	13.97230900	8.96779900	0.15981300
C	13.89458600	7.44257500	0.01991300
C	12.63373300	6.83281100	0.64429500
C	12.55691600	5.30764600	0.50136600
C	11.29296000	4.70433400	1.12598800
C	11.23730700	3.18949000	0.98009400
O	10.30670600	-1.97002200	0.74338900
C	13.43331800	-16.79107000	-0.56000400
C	12.34108000	-15.71974600	-0.48950000
C	12.89519900	-14.29770300	-0.33861800
C	11.80810000	-13.21854000	-0.26798400
C	12.36254100	-11.79664800	-0.11723200
C	11.27480800	-10.71779400	-0.04802900
C	11.82926000	-9.29588200	0.10244200
C	10.74102300	-8.21737700	0.16946100
C	11.29614300	-6.79587100	0.31958200
C	10.20649600	-5.71832600	0.38343900
C	10.77008900	-4.29996200	0.53435600
C	9.67675300	-3.24258900	0.58953000
O	-10.18126300	-2.64185300	-0.38330500
C	-18.91920500	-14.65947800	2.88270400
C	-18.24333100	-13.39260900	3.41560600
C	-17.45494600	-12.62918000	2.34434700
C	-16.77538100	-11.35875900	2.86936300
C	-15.98615600	-10.59616900	1.79816500
C	-15.30811700	-9.32485200	2.32347700
C	-14.51673400	-8.56358700	1.25293200
C	-13.84104000	-7.29096900	1.77815700
C	-13.04635100	-6.53239100	0.70839200
C	-12.37445100	-5.25781700	1.23426700
C	-11.57566500	-4.50879000	0.16068600
C	-10.92687600	-3.24070000	0.69389900
O	-11.63536000	-0.30749700	0.07534100
C	-25.10053400	1.78326700	-6.69450800
C	-23.57722600	1.84935900	-6.83910800

C	-22.82854600	1.43677900	-5.56582300
C	-21.30248200	1.49998100	-5.70195700
C	-20.55403300	1.08693700	-4.42872200
C	-19.02787300	1.15040700	-4.56556000
C	-18.27929300	0.73658200	-3.29265800
C	-16.75317800	0.80026200	-3.43006500
C	-16.00505900	0.38534300	-2.15745400
C	-14.47891200	0.44923800	-2.29657300
C	-13.73796300	0.03224600	-1.02028500
C	-12.22675200	0.10051300	-1.17353200
O	-10.36640600	2.02600100	0.50025200
C	-13.58851100	16.64520900	3.15385000
C	-12.49163300	15.58509800	3.01711700
C	-13.03621700	14.18530300	2.70721300
C	-11.94429800	13.11772300	2.56824900
C	-12.48895000	11.71795200	2.25853400
C	-11.39603100	10.65121500	2.11912900
C	-11.94016800	9.25117300	1.80975800
C	-10.84614600	8.18563200	1.66971000
C	-11.39016900	6.78558100	1.36103500
C	-10.29392300	5.72215600	1.22021700
C	-10.84562000	4.32451400	0.91344300
C	-9.74528800	3.28248500	0.77353400
H	3.87264200	-2.61280900	0.27772200
H	2.00939000	-4.21529300	-0.11533700
H	-0.33694400	-3.50528200	-0.08361000
H	-3.92593300	2.66442500	0.94816400
H	-2.06261900	4.26657300	1.34096300
H	0.28375300	3.55635000	1.30844600
H	2.78314600	2.35261700	1.18799300
H	-2.83705700	-2.30111200	0.03741300
H	7.55170000	2.43796500	1.40914600
H	7.59288700	-1.79529200	0.61426300
H	-7.60570700	-2.39121800	-0.16238900
H	-7.63848100	1.85064100	0.60241000
H	26.61533900	-2.83032800	-3.60476400
H	26.31575400	-1.73091000	-2.25236300
H	26.01572000	-3.46237300	-2.06577700
H	24.16550400	-3.32024500	-3.78376100

H	24.46334300	-1.60150900	-3.96894100
H	23.85325600	-1.23525200	-1.55750300
H	23.55570700	-2.95494600	-1.37177100
H	21.73885000	-2.82852200	-3.10819000
H	22.03669600	-1.10898300	-3.29502100
H	21.42554900	-0.74043200	-0.88264800
H	21.12864900	-2.45992100	-0.69469600
H	19.31208700	-2.33690700	-2.43048200
H	19.60972200	-0.61774300	-2.62096500
H	18.99952200	-0.24389500	-0.20941500
H	18.70331100	-1.96301900	-0.01707800
H	16.88549800	-1.84534400	-1.75132800
H	17.18295100	-0.12668600	-1.94778400
H	16.57408400	0.25551200	0.46282300
H	16.27825100	-1.46294000	0.66177000
H	14.46142000	-1.35353300	-1.07081300
H	14.75926700	0.36459400	-1.27626000
H	14.14310300	0.76177900	1.13708500
H	13.84339800	-0.95873600	1.34531800
H	12.02328600	-0.87650700	-0.34594900
H	12.32168800	0.85928400	-0.59381000
H	18.88845200	15.77493600	-1.76097000
H	17.12192400	15.85356300	-1.78762500
H	17.97454600	15.67235200	-0.25042200
H	18.79727500	13.39596900	-0.98256300
H	17.95085400	13.57582300	-2.50844200
H	15.75989100	13.69039300	-1.28011700
H	16.60676700	13.51028500	0.24657800
H	17.46022400	11.26105100	-0.49210600
H	16.61376000	11.44129000	-2.01903000
H	14.42108600	11.55476500	-0.78986700
H	15.26763200	11.37364800	0.73682600
H	16.12202500	9.12587200	-0.00346700
H	15.27611900	9.30727200	-1.53057600
H	13.08295800	9.41997900	-0.30192400
H	13.92909000	9.23700200	1.22482100
H	14.78360000	6.99017500	0.48222300
H	13.93831900	7.17362400	-1.04524700
H	11.74440800	7.28473500	0.18291600

H	12.58994600	7.09887400	1.70976800
H	13.44570400	4.85579400	0.96421100
H	12.60226900	5.04272300	-0.56475200
H	10.39795600	5.13905600	0.66417300
H	11.24659300	4.95281000	2.19363200
H	12.11224100	2.71789600	1.43712700
H	11.21016100	2.90439100	-0.08090900
H	13.00603200	-17.79338000	-0.66745300
H	14.10058900	-16.62122200	-1.41271000
H	14.05029100	-16.78832200	0.34583100
H	11.66911600	-15.93682900	0.35201700
H	11.71910600	-15.77107200	-1.39357500
H	13.56857400	-14.08084300	-1.18039000
H	13.51783500	-14.24633300	0.56620400
H	11.13507600	-13.43637200	0.57364900
H	11.18590500	-13.27099100	-1.17292600
H	13.03611000	-11.57896500	-0.95844200
H	12.98395900	-11.74371800	0.78814600
H	10.60120000	-10.93522200	0.79317000
H	10.65345200	-10.77073400	-0.95346800
H	12.50370500	-9.07864900	-0.73814100
H	12.44938700	-9.24216500	1.00859600
H	10.06658600	-8.43409000	1.01010500
H	10.12102300	-8.27091400	-0.73683400
H	11.97126500	-6.57847100	-0.52008700
H	11.91370000	-6.74017100	1.22698900
H	9.53154700	-5.93578200	1.22316600
H	9.58973400	-5.77387300	-0.52476000
H	11.43994900	-4.07135600	-0.30392800
H	11.37281700	-4.22799700	1.44770700
H	8.99937300	-3.42589200	1.43547800
H	9.07586800	-3.25609100	-0.33127500
H	-19.47294700	-15.18075200	3.67039600
H	-19.62682500	-14.42335500	2.07973700
H	-18.18187300	-15.36060800	2.47529600
H	-17.56829600	-13.65736900	4.24087400
H	-19.00264900	-12.72702500	3.84837100
H	-18.13036900	-12.36512400	1.51783100
H	-16.69507300	-13.29550600	1.91116800

H	-16.10119000	-11.62360900	3.69647700
H	-17.53603300	-10.69309400	3.30193100
H	-16.65991700	-10.33222200	0.97044700
H	-15.22468400	-11.26142400	1.36652300
H	-14.63566000	-9.58849300	3.15233500
H	-16.06985800	-8.65885600	2.75352000
H	-15.18849800	-8.30139200	0.42305800
H	-13.75364500	-9.22896800	0.82441400
H	-13.17132300	-7.55267700	2.60980800
H	-14.60449200	-6.62427400	2.20400100
H	-13.71444700	-6.27201000	-0.12470800
H	-12.28029700	-7.19721600	0.28493700
H	-11.71001500	-5.51820300	2.07030800
H	-13.14127800	-4.59091000	1.65296500
H	-12.23109900	-4.24006000	-0.67724500
H	-10.79341700	-5.15934800	-0.24992500
H	-10.24035900	-3.47173800	1.52163500
H	-11.68108200	-2.53462600	1.05836700
H	-25.60492100	2.08322800	-7.61885400
H	-25.43170300	0.76753400	-6.44991500
H	-25.45180400	2.44503100	-5.89457000
H	-23.27996500	2.86935400	-7.11857200
H	-23.26007500	1.20418900	-7.66977200
H	-23.12659300	0.41608800	-5.28584400
H	-23.14642900	2.08216500	-4.73427500
H	-21.00549200	2.52083300	-5.98209400
H	-20.98575600	0.85491000	-6.53403800
H	-20.85081200	0.06605000	-4.14868600
H	-20.87065000	1.73186600	-3.59652500
H	-18.73100700	2.17141600	-4.84513100
H	-18.71133100	0.50583900	-5.39804400
H	-18.57586300	-0.28451500	-3.01326300
H	-18.59566800	1.38096000	-2.45997200
H	-16.45642900	1.82153500	-3.70875000
H	-16.43683900	0.15628100	-4.26303900
H	-16.30053300	-0.63602200	-1.87876800
H	-16.31981500	1.02912100	-1.32402500
H	-14.18376500	1.47097200	-2.57441700
H	-14.16466200	-0.19455200	-3.13019500

H	-14.01784900	-0.99133100	-0.74111000
H	-14.03257200	0.67831500	-0.18415700
H	-11.90454300	1.12184300	-1.41197500
H	-11.88009800	-0.56912300	-1.97225600
H	-13.16798000	17.63170300	3.37462100
H	-14.17290800	16.73220300	2.23074500
H	-14.28522800	16.39279900	3.96136600
H	-11.90228000	15.54655300	3.94339800
H	-11.79078300	15.88348000	2.22552700
H	-13.62682000	14.22408500	1.78054200
H	-13.73791500	13.88675900	3.49937100
H	-11.35399100	13.08012200	3.49501900
H	-11.24309900	13.41730300	1.77618100
H	-13.07949300	11.75536200	1.33194900
H	-13.18966800	11.41791800	3.05078000
H	-10.80531500	10.61398100	3.04559800
H	-10.69548700	10.95118100	1.32668300
H	-12.53119400	9.28805500	0.88350500
H	-12.63997500	8.95047300	2.60248500
H	-10.25489900	8.14890700	2.59581200
H	-10.14665200	8.48604900	0.87654700
H	-11.98130600	6.82080800	0.43511300
H	-12.08795500	6.48307500	2.15441900
H	-9.70269000	5.68788500	2.14620200
H	-9.59705200	6.02462800	0.42571800
H	-11.42962000	4.34550800	-0.01491200
H	-11.53054700	4.00541700	1.70824100
H	-9.15507000	3.21307900	1.69856800
H	-9.05861700	3.54803400	-0.04343600

CPA dimer 1a$_{6,6}$

C	-8.92686800	1.86039600	-0.89354400
C	-9.39990700	0.49253200	-0.90042900
C	-8.50736800	-0.57805300	-0.90474100
C	-7.10122400	-0.35221300	-0.90239400
C	-6.67706700	1.00011500	-0.89559800
C	-7.57883000	2.10689400	-0.89121600
C	-6.05210000	-1.30378300	-0.90612700

C	-4.72670000	-0.82729000	-0.90295100
C	-4.30303600	0.52485400	-0.89645400
C	-5.35227700	1.47700300	-0.89253400
C	-3.82541900	-1.93472900	-0.90775500
C	-2.47705200	-1.68914400	-0.90608200
C	-2.00543900	-0.32146300	-0.89973200
C	-2.89733800	0.75025300	-0.89499900
C	-5.43406500	2.94202200	-0.88585400
C	-4.65857400	-3.14537800	-0.91410900
C	-5.97033600	-2.76869100	-0.91317100
C	-6.74600100	3.31805400	-0.88506100
C	-10.80303800	0.25884400	-0.90277100
C	-12.00584700	0.07096400	-0.90507600
C	-13.41126800	-0.15615700	-0.90811400
C	-14.30253900	0.93786900	-0.90851100
C	-15.67582900	0.71225500	-0.91731600
C	-16.19512900	-0.60714100	-0.89944500
C	-15.29837300	-1.69120600	-0.89814600
C	-13.91815900	-1.46035100	-0.90452300
C	8.92683100	-1.85871900	-0.89397100
C	9.39985700	-0.49084700	-0.90031200
C	8.50730700	0.57973100	-0.90426000
C	7.10116500	0.35387600	-0.90202900
C	6.67702200	-0.99845900	-0.89574800
C	7.57879600	-2.10523100	-0.89176700
C	6.05202900	1.30543500	-0.90545300
C	4.72663500	0.82892700	-0.90249500
C	4.30298600	-0.52322500	-0.89650300
C	5.35223700	-1.47536300	-0.89289900
C	3.82534000	1.93635600	-0.90693700
C	2.47697500	1.69075400	-0.90540600
C	2.00538000	0.32306400	-0.89956100
C	2.89729200	-0.74864100	-0.89517500
C	5.43404100	-2.94038400	-0.88674700
C	4.65848100	3.14701700	-0.91283100
C	5.97024800	2.77034500	-0.91198000
C	6.74598000	-3.31640200	-0.88607300
C	-0.60269600	-0.08669300	-0.89894200
C	0.60264000	0.08826500	-0.89889300

C	10.80298700	-0.25715400	-0.90253300
C	12.00579900	-0.06929400	-0.90475300
C	13.41123000	0.15775300	-0.90768900
C	14.30243500	-0.93632200	-0.90808600
C	15.67574000	-0.71078700	-0.91681300
C	16.19511200	0.60857600	-0.89882600
C	15.29841500	1.69269500	-0.89750000
C	13.91819400	1.46192200	-0.90397300
O	-16.61308600	1.70454700	-0.96914300
C	-21.95858900	15.84803000	-2.51279500
C	-20.71262700	14.96430400	-2.40175800
C	-21.03842600	13.47350200	-2.24878100
C	-19.79672700	12.58071000	-2.13696100
C	-20.12401800	11.09026600	-1.98408300
C	-18.88271100	10.19657700	-1.87280000
C	-19.21219400	8.70660500	-1.72006200
C	-17.97201100	7.81120800	-1.60974100
C	-18.30542100	6.32224700	-1.45736700
C	-17.06611800	5.42513800	-1.34836700
C	-17.41253800	3.93867600	-1.19793900
C	-16.17523200	3.05900600	-1.09002000
O	-17.54507900	-0.82957500	-1.00796900
C	-32.12133200	0.92631200	2.97481300
C	-30.65888400	1.02366600	3.41920600
C	-29.66344600	0.63776800	2.31820800
C	-28.19648500	0.73254500	2.75460600
C	-27.20151700	0.34649300	1.65328400
C	-25.73447700	0.44180000	2.08989500
C	-24.73981300	0.05548500	0.98839400
C	-23.27273800	0.15153400	1.42478900
C	-22.27906700	-0.23505800	0.32273000
C	-20.81198300	-0.13771600	0.75955500
C	-19.82517800	-0.52540000	-0.34856900
C	-18.37490500	-0.41652100	0.09657100
O	-15.67171400	-3.00261300	-0.96487100
C	-21.77451600	-15.88700100	4.31345800
C	-21.92017200	-14.36773300	4.18560100
C	-20.75233200	-13.70682500	3.44308000
C	-20.89000900	-12.18536600	3.31035700

C	-19.72231500	-11.52519700	2.56701700
C	-19.86037000	-10.00362900	2.43400800
C	-18.69327800	-9.34419600	1.68907700
C	-18.83151500	-7.82269200	1.55534400
C	-17.66521100	-7.16497000	0.80800800
C	-17.80451900	-5.64345900	0.67343700
C	-16.63583500	-4.99403200	-0.07765300
C	-16.79521400	-3.48557600	-0.21225500
O	16.61295700	-1.70312700	-0.96858000
C	21.95797500	-15.84673000	-2.51284000
C	20.71204600	-14.96296200	-2.40176500
C	21.03789900	-13.47218100	-2.24870600
C	19.79623300	-12.57934800	-2.13685300
C	20.12357700	-11.08892500	-1.98388900
C	18.88230100	-10.19519500	-1.87258400
C	19.21183600	-8.70524400	-1.71975400
C	17.97168200	-7.80980700	-1.60943100
C	18.30514100	-6.32086800	-1.45695600
C	17.06586500	-5.42371600	-1.34798500
C	17.41233300	-3.93727700	-1.19744500
C	16.17505500	-3.05756000	-1.08959900
O	17.54508100	0.83091000	-1.00726800
C	32.12085000	-0.93887300	2.97116300
C	30.65849600	-1.03321900	3.41651400
C	29.66304300	-0.64798700	2.31529700
C	28.19617500	-0.73979600	2.75264000
C	27.20119300	-0.35436900	1.65111200
C	25.73424200	-0.44679800	2.08864000
C	24.73956700	-0.06102700	0.98695800
C	23.27257600	-0.15434500	1.42422400
C	22.27889800	0.23182600	0.32202200
C	20.81188900	0.13700900	0.75965200
C	19.82508400	0.52442300	-0.34856400
C	18.37486300	0.41779000	0.09728400
O	15.67185400	3.00407900	-0.96412000
C	21.77564500	15.88741800	4.31562800
C	21.92120900	14.36815800	4.18757300
C	20.75333800	13.70741800	3.44495300
C	20.89092400	12.18596900	3.31202800

C	19.72320600	11.52597200	2.56857400
C	19.86114200	10.00440700	2.43540800
C	18.69403200	9.34515300	1.69034700
C	18.83212200	7.82364600	1.55649800
C	17.66579500	7.16610600	0.80903700
C	17.80493400	5.64458800	0.67437700
C	16.63621200	4.99534700	-0.07681400
C	16.79540000	3.48687500	-0.21146000
H	-9.66310900	2.65844600	-0.89063900
H	-8.89964200	-1.58937300	-0.91035100
H	-1.74090300	-2.48718300	-0.90970200
H	-2.50473500	1.76152900	-0.89038900
H	-4.58391900	3.61311400	-0.88229500
H	-4.28297800	-4.16086500	-0.91898900
H	-6.82076000	-3.43939300	-0.91728900
H	-7.12183000	4.33350400	-0.88082200
H	-13.90096700	1.94186700	-0.91961600
H	-13.25437300	-2.31626100	-0.90418200
H	9.66308100	-2.65676100	-0.89133400
H	8.89957200	1.59105700	-0.90950000
H	1.74081700	2.48878500	-0.90877000
H	2.50470100	-1.75992400	-0.89093000
H	4.58390200	-3.61148600	-0.88344600
H	4.28287300	4.16250100	-0.91736500
H	6.82066400	3.44105800	-0.91583100
H	7.12181900	-4.33185000	-0.88219700
H	13.90080800	-1.94029700	-0.91925600
H	13.25445000	2.31786400	-0.90361700
H	-22.56689100	15.56817100	-3.38045500
H	-22.59182800	15.75379700	-1.62322800
H	-21.69290600	16.90468400	-2.62065600
H	-20.10684200	15.29148500	-1.54572100
H	-20.08219800	15.10708700	-3.29000100
H	-21.66996500	13.33077900	-1.36005600
H	-21.64534700	13.14634200	-3.10530100
H	-19.19025100	12.90858600	-1.28054100
H	-19.16576300	12.72413600	-3.02581400
H	-20.75478200	10.94688400	-1.09509700
H	-20.73082100	10.76265100	-2.84031300

H	-18.27566800	10.52359900	-1.01651600
H	-18.25198600	10.33933100	-2.76186100
H	-19.84253900	8.56389500	-0.83070700
H	-19.81979600	8.37999500	-2.57604800
H	-17.36407000	8.13674500	-0.75357700
H	-17.34175900	7.95285100	-2.49923700
H	-18.93452400	6.17976900	-0.56740200
H	-18.91322800	5.99629500	-2.31305800
H	-16.45847700	5.74974600	-0.49184700
H	-16.43739500	5.56695200	-2.23861900
H	-18.03221500	3.78658400	-0.30543900
H	-18.00726700	3.60103100	-2.05524400
H	-15.57294900	3.33942000	-0.21385400
H	-15.54163300	3.16580800	-1.98170000
H	-32.80566800	1.20779400	3.78179800
H	-32.37443600	-0.09417900	2.66540500
H	-32.32173900	1.58705000	2.12365400
H	-30.49832800	0.37851200	4.29375600
H	-30.44604300	2.04741500	3.75608700
H	-29.87686300	-0.38670900	1.98070100
H	-29.82466200	1.28306200	1.44263300
H	-28.03633700	0.08737100	3.63033800
H	-27.98415200	1.75713000	3.09218500
H	-27.41356900	-0.67814800	1.31591100
H	-27.36175900	0.99143000	0.77745500
H	-25.57410400	-0.20304000	2.96578700
H	-25.52228500	1.46652700	2.42703400
H	-24.95154100	-0.96938300	0.65161200
H	-24.90035600	0.69994600	0.11231500
H	-23.11191900	-0.49289100	2.30089500
H	-23.06078000	1.17653600	1.76120900
H	-22.48940100	-1.26024500	-0.01323300
H	-22.43889400	0.40879500	-0.55355600
H	-20.65249800	-0.78166500	1.63612600
H	-20.60194800	0.88784900	1.09475000
H	-20.02070300	-1.55257100	-0.68203400
H	-19.96698100	0.11984700	-1.22399500
H	-18.17840700	-1.07121400	0.95836400
H	-18.13220700	0.61074600	0.38164800

H	-21.71750600	-16.36395100	3.32833100
H	-20.86293200	-16.15307700	4.86065500
H	-22.62266800	-16.32810900	4.84718300
H	-22.01012000	-13.92313100	5.18615000
H	-22.85861600	-14.13248400	3.66518300
H	-19.81299500	-13.94282400	3.96362400
H	-20.66200300	-14.15232800	2.44189000
H	-20.98014400	-11.74074400	4.31186700
H	-21.82989200	-11.95040100	2.79051100
H	-18.78240700	-11.75995400	3.08676600
H	-19.63220700	-11.96989100	1.56561500
H	-19.94939400	-9.55858500	3.43539900
H	-20.80071000	-9.76884300	1.91497200
H	-17.75286000	-9.57861800	2.20797900
H	-18.60431800	-9.78936700	0.68783400
H	-18.91874100	-7.37696200	2.55659400
H	-19.77252400	-7.58819500	1.03741300
H	-16.72395900	-7.39790700	1.32548300
H	-17.57787600	-7.60968400	-0.19326200
H	-17.89045600	-5.19885100	1.67530400
H	-18.74634400	-5.41092000	0.15650700
H	-15.69030600	-5.20756300	0.43583300
H	-16.55050500	-5.42510000	-1.08296000
H	-16.80984900	-3.00809500	0.77745000
H	-17.72702200	-3.23259100	-0.72627600
H	22.56629300	-15.56684700	-3.38048000
H	22.59121000	-15.75257000	-1.62326300
H	21.69225200	-16.90336800	-2.62076200
H	20.10624100	-15.29016700	-1.54575100
H	20.08161700	-15.10567400	-3.29002000
H	21.66943400	-13.32952800	-1.35996600
H	21.64484100	-13.14499900	-3.10520200
H	19.18973200	-12.90724800	-1.28045900
H	19.16527600	-12.72270200	-3.02572300
H	20.75432900	-10.94561300	-1.09488200
H	20.73041000	-10.76128700	-2.84008900
H	18.27522300	-10.52224400	-1.01633500
H	18.25159400	-10.33787500	-2.76167000
H	19.84215400	-8.56260600	-0.83036800

H	19.81948100	-8.37861200	-2.57570100
H	17.36369200	-8.13537200	-0.75331400
H	17.34146300	-7.95137300	-2.49896400
H	18.93419600	-6.17846100	-0.56694600
H	18.91301100	-5.99489300	-2.31259400
H	16.45815300	-5.74835200	-0.49152700
H	16.43719900	-5.56545300	-2.23828900
H	18.03193200	-3.78525400	-0.30488100
H	18.00715500	-3.59961200	-2.05467900
H	15.57266400	-3.33800900	-0.21351900
H	15.54154700	-3.16426800	-1.98135500
H	32.80520000	-1.21979600	3.77833100
H	32.37528700	0.08064400	2.65964500
H	32.31983700	-1.60155200	2.12118000
H	30.49935100	-0.38614000	4.28989900
H	30.44433100	-2.05600100	3.75548600
H	29.87777200	0.37552900	1.97571300
H	29.82286200	-1.29519400	1.44087800
H	28.03742700	-0.09273700	3.62723600
H	27.98252500	-1.76343200	3.09226400
H	27.41452300	0.66934700	1.31174100
H	27.36007500	-1.00114800	0.77639400
H	25.57523600	0.19983300	2.96346000
H	25.52076200	-1.47061800	2.42771600
H	24.95252200	0.96297400	0.64831600
H	24.89881100	-0.70720700	0.11190800
H	23.11306800	0.49172400	2.29935900
H	23.05937600	-1.17850700	1.76241400
H	22.49038400	1.25623000	-0.01560000
H	22.43750800	-0.41356700	-0.55335200
H	20.65363300	0.78239100	1.63539100
H	20.60068300	-0.88781400	1.09638400
H	20.02164200	1.55095200	-0.68339700
H	19.96577600	-0.12208500	-1.22323700
H	18.17955700	1.07352900	0.95855400
H	18.13100200	-0.60890700	0.38340500
H	20.86407100	16.15347900	4.86284900
H	22.62381700	16.32840500	4.84941900
H	21.71867500	16.36449900	3.33056200

H	22.01111800	13.92342000	5.18806500
H	22.85964600	14.13292000	3.66713700
H	19.81401000	13.94340300	3.96551900
H	20.66304500	14.15306200	2.44382100
H	20.98101100	11.74120800	4.31348000
H	21.83080400	11.95101500	2.79217100
H	18.78329600	11.76074900	3.08831200
H	19.63317200	11.97078200	1.56721600
H	19.95007600	9.55925000	3.43675700
H	20.80148800	9.76959800	1.91639700
H	17.75360400	9.57962700	2.20920600
H	18.60518300	9.79041700	0.68913500
H	18.91924100	7.37782600	2.55771700
H	19.77313900	7.58909300	1.03860400
H	16.72453300	7.39911800	1.32646100
H	17.57858100	7.61089600	-0.19221000
H	17.89076800	5.19990900	1.67622200
H	18.74675800	5.41196900	0.15748200
H	15.69067800	5.20898600	0.43662000
H	16.55100400	5.42646300	-1.08211000
H	16.80993700	3.00936400	0.77822800
H	17.72719100	3.23377600	-0.72546100

Dimer Ph_2-1TB

C	-6.27880100	-0.14742200	-0.15129000
C	-7.49531600	-0.18097400	-0.17920300
C	-8.91827500	-0.21957900	-0.21201500
C	-9.60073300	-1.42912400	-0.00903400
C	-10.99528700	-1.45641900	-0.03480300
C	-11.72713800	-0.28378800	-0.27154400
C	-11.04145200	0.93335000	-0.48395400
C	-9.64757700	0.96399700	-0.45483400
C	0.60849700	0.01463200	0.01479400
C	-0.60863400	-0.01402400	-0.01466000
C	6.27866700	0.14779200	0.15151900

C	7.49518500	0.18124500	0.17941700
C	8.91814600	0.21982200	0.21215200
C	9.60062100	1.42934800	0.00912600
C	10.99517800	1.45660500	0.03481200
C	11.72701600	0.28396500	0.27153300
C	11.04131200	-0.93315500	0.48400500
C	9.64743700	-0.96377000	0.45494900
O	-11.63866300	-2.63928000	0.23450300
C	-20.00336400	-14.79927800	-3.44970600
C	-19.33078100	-13.51713500	-3.94916700
C	-18.59759500	-12.74672600	-2.84421100
C	-17.92119900	-11.46124900	-3.33555000
C	-17.18830800	-10.69123200	-2.23016800
C	-16.51238300	-9.40537000	-2.72166500
C	-15.77968400	-8.63563000	-1.61596400
C	-15.10452100	-7.34925700	-2.10721300
C	-14.37214200	-6.58048200	-1.00085800
C	-13.69858800	-5.29326300	-1.49266600
C	-12.96712800	-4.53161700	-0.38063400
C	-12.31325400	-3.25276600	-0.88083400
O	-13.09640600	-0.33013700	-0.33570100
C	-27.00987300	1.77301600	5.45274500
C	-25.50059900	1.85737000	5.69911900
C	-24.66339100	1.41709100	4.49194300
C	-23.15075600	1.49866300	4.72999600
C	-22.31381200	1.05895700	3.52247100
C	-20.80113100	1.14140100	3.76101000
C	-19.96402900	0.70297100	2.55313300
C	-18.45144900	0.78650400	2.79203200
C	-17.61468000	0.35012500	1.58339900
C	-16.10222100	0.43510400	1.82381600
C	-15.27208700	0.00070000	0.60971900
C	-13.77561000	0.09207100	0.86286700

O	-11.83747600	2.01930900	-0.69738400
C	-15.21259300	16.52843800	-3.71258700
C	-14.09914900	15.50318300	-3.47842600
C	-14.62507200	14.08900300	-3.20363200
C	-13.51694500	13.05555200	-2.96814100
C	-14.04402800	11.64191900	-2.69297600
C	-12.93612100	10.60775500	-2.45849100
C	-13.46485900	9.19492300	-2.18241000
C	-12.35785600	8.15939600	-1.94957400
C	-12.88969400	6.74816300	-1.67224000
C	-11.78322600	5.71129200	-1.44157500
C	-12.32688100	4.30443400	-1.16398600
C	-11.22007500	3.28458000	-0.93816200
O	13.09628400	0.33030000	0.33564000
C	27.00966600	-1.77425800	-5.45246800
C	25.50039100	-1.85848700	-5.69888300
C	24.66318700	-1.41810100	-4.49174300
C	23.15055200	-1.49954700	-4.72983500
C	22.31361400	-1.05973300	-3.52234400
C	20.80093200	-1.14204900	-3.76091900
C	19.96384000	-0.70351400	-2.55307300
C	18.45125800	-0.78691500	-2.79200400
C	17.61450600	-0.35043700	-1.58339500
C	16.10204200	-0.43527600	-1.82383500
C	15.27193400	-0.00078800	-0.60975200
C	13.77545100	-0.09200300	-0.86291400
O	11.63858200	2.63943900	-0.23455400
C	20.00421800	14.79904900	3.44876700
C	19.33154800	13.51699100	3.94833300
C	18.59831600	12.74653800	2.84343800
C	17.92182900	11.46115000	3.33488100
C	17.18889500	10.69108800	2.22955900
C	16.51287000	9.40531800	2.72115800

C	15.78012900	8.63553900	1.61551200
C	15.10485300	7.34926300	2.10686000
C	14.37243100	6.58045600	1.00055600
C	13.69874400	5.29334400	1.49245700
C	12.96724100	4.53167600	0.38046700
C	12.31320700	3.25294100	0.88075300
O	11.83732400	-2.01912000	0.69744700
C	15.21261400	-16.52804000	3.71335900
C	14.09915200	-15.50287600	3.47889000
C	14.62503600	-14.08865700	3.20422000
C	13.51688800	-13.05529500	2.96843300
C	14.04393600	-11.64162600	2.69338200
C	12.93601000	-10.60754000	2.45864000
C	13.46472000	-9.19467900	2.18265000
C	12.35770500	-8.15920800	1.94962200
C	12.88952700	-6.74795700	1.67234800
C	11.78305400	-5.71111400	1.44158000
C	12.32671100	-4.30425300	1.16401000
C	11.21991100	-3.28438700	0.93821000
H	-9.06014000	-2.34801300	0.18390500
H	-9.10604000	1.88702800	-0.61300500
H	9.06004100	2.34824900	-0.18379600
H	9.10588300	-1.88678500	0.61316000
H	-20.51726800	-15.32528700	-4.26089600
H	-19.26946600	-15.48971300	-3.01853000
H	-20.74511300	-14.58097100	-2.67293200
H	-20.08504000	-12.86241400	-4.40664400
H	-18.62022800	-13.76443900	-4.74966400
H	-17.84305900	-13.40229000	-2.38601200
H	-19.30868600	-12.49982300	-2.04268300
H	-18.67633100	-10.80664800	-3.79390600
H	-17.21063700	-11.70902900	-4.13711600
H	-16.43294800	-11.34564800	-1.77203800

H	-17.89877600	-10.44373600	-1.42846600
H	-17.26772700	-8.75087000	-3.17972900
H	-15.80182000	-9.65272300	-3.52330600
H	-15.02390500	-9.28984000	-1.15829300
H	-16.49010900	-8.38874800	-0.81405700
H	-15.86026300	-6.69478200	-2.56459600
H	-14.39398500	-7.59591500	-2.90905700
H	-13.61505500	-7.23351100	-0.54423400
H	-15.08185100	-6.33376500	-0.19857800
H	-14.45597300	-4.63957900	-1.94779800
H	-12.98960600	-5.54041900	-2.29548500
H	-12.19623700	-5.16948000	0.06947100
H	-13.66842700	-4.27379200	0.42288000
H	-13.05882400	-2.56092600	-1.28759200
H	-11.57824300	-3.47241600	-1.66906300
H	-27.57865600	2.09350000	6.33170400
H	-27.31042700	2.40923500	4.61243800
H	-27.31588200	0.74788500	5.21468700
H	-25.23623800	1.23826400	6.56724800
H	-25.23084100	2.88740600	5.96949600
H	-24.92855500	2.03633800	3.62287800
H	-24.93374700	0.38629600	4.22103000
H	-22.88658900	0.87927800	5.59911400
H	-22.88161800	2.52950800	5.00155500
H	-22.57810900	1.67816500	2.65329600
H	-22.58249500	0.02802500	3.25101300
H	-20.53662000	0.52165100	4.62970900
H	-20.53265500	2.17222600	4.03316900
H	-20.22867500	1.32253200	1.68434400
H	-20.23184800	-0.32800200	2.28108500
H	-18.18641000	0.16595400	3.65995800
H	-18.18390700	1.81730000	3.06517600
H	-17.87851500	0.97051000	0.71527300

H	-17.88043500	-0.68083300	1.31019800
H	-15.83834500	-0.18714400	2.69060300
H	-15.83741600	1.46595200	2.09855600
H	-15.51674100	0.62551400	-0.25806600
H	-15.52093200	-1.03192500	0.33452400
H	-13.47522000	-0.55836200	1.69551900
H	-13.48307100	1.12173800	1.10318200
H	-14.80533900	17.52627700	-3.90550000
H	-15.83261200	16.25068700	-4.57260200
H	-15.87322500	16.60199400	-2.84117700
H	-13.47502300	15.82692900	-2.63434200
H	-13.43482700	15.47803000	-4.35298900
H	-15.25038000	13.76534300	-4.04817300
H	-15.29039200	14.11439100	-2.32856600
H	-12.89178000	13.38019500	-2.12401500
H	-12.85229000	13.03076000	-3.84357600
H	-14.66980800	11.31762400	-3.53672400
H	-14.70810900	11.66666500	-1.81713900
H	-12.30975600	10.93192000	-1.61513100
H	-12.27257800	10.58199300	-3.33467700
H	-14.09223800	8.87130400	-3.02516900
H	-14.12744100	9.22060400	-1.30551000
H	-11.72963900	8.48268100	-1.10730100
H	-11.69613100	8.13204300	-2.82702600
H	-13.51853100	6.42451000	-2.51349400
H	-13.54960900	6.77442900	-0.79368500
H	-11.15377800	6.03486300	-0.60053200
H	-11.12466000	5.68351100	-2.32114600
H	-12.94724900	3.96663300	-2.00286400
H	-12.97585200	4.31957100	-0.27970000
H	-10.59697800	3.56868500	-0.07769800
H	-10.56566300	3.21844600	-1.81912100
H	27.57844600	-2.09481700	-6.33140100

H	27.31575700	-0.74914700	-5.21443000
H	27.31014000	-2.41047900	-4.61213400
H	25.23055100	-2.88850700	-5.96923800
H	25.23610900	-1.23938100	-6.56703600
H	24.93362600	-0.38732100	-4.22085000
H	24.92827300	-2.03734700	-3.62265300
H	22.88133100	-2.53037500	-5.00137300
H	22.88646300	-0.88016200	-5.59897600
H	22.58238100	-0.02881700	-3.25090800
H	22.57783400	-1.67894000	-2.65314500
H	20.53237100	-2.17285700	-4.03305900
H	20.53649700	-0.52229700	-4.62964000
H	20.23174500	0.32744200	-2.28104600
H	20.22841000	-1.32307700	-1.68426200
H	18.18362800	-1.81769200	-3.06513500
H	18.18629300	-0.16635700	-3.65994600
H	17.88035100	0.68050100	-1.31020700
H	17.87826600	-0.97083100	-0.71525300
H	15.83714400	-1.46610000	-2.09857700
H	15.83823900	0.18699300	-2.69062900
H	15.52087800	1.03181400	-0.33455700
H	15.51651400	-0.62562300	0.25803900
H	13.48280200	-1.12164200	-1.10321600
H	13.47513800	0.55844900	-1.69558100
H	20.51815300	15.32509100	4.25991500
H	20.74595600	14.58062700	2.67201500
H	19.27036900	15.48949600	3.01753000
H	18.62100700	13.76441000	4.74880600
H	20.08576100	12.86225900	4.40586800
H	19.30939500	12.49952000	2.04193500
H	17.84382800	13.40211500	2.38517700
H	17.21127700	11.70904600	4.13642000
H	18.67691200	10.80653600	3.79329900

H	17.89935500	10.44347300	1.42788600
H	16.43358800	11.34551900	1.77136300
H	15.80231500	9.65279200	3.52276900
H	17.26816100	8.75080200	3.17928800
H	16.49055000	8.38853000	0.81363900
H	15.02441200	9.28976900	1.15777100
H	14.39432200	7.59604800	2.90867000
H	15.86053200	6.69476400	2.56431200
H	15.08213800	6.33360500	0.19831500
H	13.61541600	7.23351600	0.54385500
H	12.98976300	5.54063500	2.29523500
H	14.45605300	4.63962500	1.94766300
H	13.66854000	4.27370000	-0.42299800
H	12.19643600	5.16958400	-0.06972300
H	11.57819700	3.47273500	1.66894200
H	13.05869200	2.56105200	1.28758500
H	14.80538800	-17.52590900	3.90617300
H	15.87348700	-16.60155200	2.84212700
H	15.83238000	-16.25023100	4.57353800
H	13.43459400	-15.47776600	4.35327500
H	13.47527800	-15.82668100	2.63464200
H	15.29058500	-14.11400000	2.32932700
H	15.25009800	-13.76493900	4.04892100
H	12.85201100	-13.03054200	3.84370000
H	12.89196300	-13.37999800	2.12415300
H	14.70821900	-11.66633100	1.81769900
H	14.66949700	-11.31728200	3.53727300
H	12.27227800	-10.58180600	3.33468400
H	12.30984900	-10.93175800	1.61515000
H	14.12746000	-9.22032600	1.30586800
H	14.09193100	-8.87102400	3.02552000
H	11.69584400	-8.13187100	2.82697200
H	11.72963200	-8.48253400	1.10725800

H	13.54953300	-6.77420100	0.79386000
H	13.51826700	-6.42428800	2.51366700
H	11.12442500	-5.68333300	2.32110300
H	11.15367500	-6.03470700	0.60049500
H	12.97568200	-4.31937800	0.27972500
H	12.94708000	-3.96646700	2.00289400
H	10.56551700	-3.21826500	1.81918400
H	10.59679200	-3.56846500	0.07775200
C	-4.85823500	-0.11338700	-0.11725400
C	-4.11420300	-1.28471100	0.14024500
C	-4.15805900	1.09153700	-0.33989600
C	-2.72874200	-1.25238000	0.17369400
H	-4.64148200	-2.21695900	0.31318900
C	-2.77257500	1.12410900	-0.30655800
H	-4.71903900	1.99863000	-0.53892100
C	-2.02888400	-0.04727900	-0.04901300
H	-2.16773900	-2.15944000	0.37281200
H	-2.24545700	2.05655300	-0.47933000
C	2.02874900	0.04783400	0.04918000
C	2.77238900	-1.12358200	0.30677300
C	2.72866000	1.25289300	-0.17355700
C	4.15787000	-1.09106600	0.34013600
H	2.24522100	-2.05599200	0.47957200
C	4.11412500	1.28516600	-0.14009100
H	2.16770200	2.15997500	-0.37270500
C	4.85810100	0.11382400	0.11745900
H	4.71881300	-1.99817200	0.53921000
H	4.64144100	2.21739000	-0.31305000

Dimer HBC_2-1TB

C	9.15749900	-0.36078300	-1.57998700
C	8.45117200	-1.53022300	-1.17878200
C	7.03364600	-1.57491400	-1.28362500

C	6.32131900	-0.44776400	-1.78307500
C	7.02924300	0.71308300	-2.20545400
C	8.44737900	0.75505300	-2.10766300
C	6.31608300	-2.77057100	-0.89653200
C	4.89538500	-2.81727800	-0.98496000
C	4.15998800	-1.62792500	-1.41339300
C	4.87852200	-0.47507300	-1.84375400
C	4.15421400	0.65915100	-2.31228600
C	4.88362300	1.82475300	-2.81112500
C	6.30611800	1.84666200	-2.74089000
C	7.01098200	2.99213800	-3.21039800
C	8.46972700	3.02928300	-3.12354000
C	9.16972300	1.92692000	-2.55421600
C	10.58858000	1.98288900	-2.44310000
C	11.30927300	0.86750600	-1.82991800
C	10.59365300	-0.30086900	-1.43829700
C	11.31122200	-1.40095800	-0.88548100
C	10.59245300	-2.62318800	-0.52559400
C	9.17602100	-2.67385300	-0.66753500
C	8.47985500	-3.86321800	-0.30645200
C	7.02524100	-3.91837800	-0.43980500
C	6.30819500	-5.08193700	-0.12371400
C	4.92713600	-5.12928400	-0.24230800
C	4.22809000	-4.00792200	-0.66460200
C	2.76270300	-1.60219600	-1.40283000
C	2.04685100	-0.47612600	-1.82700300
C	2.75696300	0.63987300	-2.28604500
C	4.20870300	2.92136500	-3.36571800
C	4.90197900	4.03084100	-3.82684400
C	6.28590700	4.06776700	-3.74432500
C	9.19881800	4.13412700	-3.58772500
C	10.58239400	4.17027500	-3.49884200
C	11.26931900	3.10746000	-2.93065300

C	12.68948300	0.93052800	-1.61964800
C	13.39491200	-0.13637300	-1.04989800
C	12.69193800	-1.29452400	-0.69638200
C	11.27270000	-3.75235000	-0.04788900
C	10.58826200	-4.90707000	0.30180000
C	9.20793500	-4.95984600	0.17812400
C	0.62288900	-0.46538800	-1.79232200
C	-6.28869500	-0.36471300	-1.54304000
C	-6.99685100	-1.48579600	-1.02428100
C	-8.41272100	-1.43638500	-0.89877000
C	-9.12346200	-0.27468000	-1.31394500
C	-8.41762000	0.83583900	-1.85827400
C	-6.99961000	0.79561500	-1.96158300
C	-9.13024500	-2.56334700	-0.34216200
C	-10.54308400	-2.50405700	-0.17071400
C	-11.27229800	-1.30801400	-0.59289800
C	-10.56029300	-0.21950400	-1.17555800
C	-11.28325600	0.92958900	-1.60902200
C	-10.56558100	2.04010100	-2.23447300
C	-9.14465400	1.99833600	-2.32119700
C	-8.44717600	3.10298300	-2.88859000
C	-6.98653300	3.08727700	-2.93656100
C	-6.27901000	1.93541000	-2.48721300
C	-4.85614100	1.92009600	-2.55316000
C	-4.12552500	0.73279700	-2.10935800
C	-4.84750500	-0.40053300	-1.63419800
C	-4.12830700	-1.56493200	-1.23640600
C	-4.86154300	-2.74510100	-0.77958300
C	-6.27754500	-2.68189600	-0.64206100
C	-6.98173800	-3.81611900	-0.14599400
C	-8.42814400	-3.73962500	0.04850000
C	-9.14146600	-4.80283900	0.62110100
C	-10.51575900	-4.73367000	0.79426500

C	-11.20899100	-3.59971700	0.39713700
C	-12.65849100	-1.21718900	-0.43860000
C	-13.37027100	-0.08099700	-0.84186800
C	-12.66766600	0.98040400	-1.42498000
C	-11.25033000	3.14756000	-2.75474600
C	-10.56592000	4.20636500	-3.33347000
C	-9.18037200	4.18723800	-3.39293400
C	-6.26309200	4.19062100	-3.41253900
C	-4.87752800	4.17025400	-3.46869100
C	-4.18259200	3.04556300	-3.04895600
C	-2.72898200	0.69001500	-2.14664800
C	-2.01736900	-0.44157800	-1.73062600
C	-2.73157000	-1.55888500	-1.28176800
C	-4.19722100	-3.94189300	-0.47578900
C	-4.89395200	-5.05375900	-0.02541100
C	-6.26888100	-4.98858700	0.14517000
C	-0.59336900	-0.45508800	-1.76358900
C	14.80015400	-0.04602500	-0.83631800
C	16.00085800	0.03201300	-0.65710200
C	17.40745100	0.11812300	-0.44971800
C	18.11836500	-0.97013400	0.09057800
C	19.49085100	-0.87678700	0.28352600
C	20.19579700	0.30485000	-0.02885000
C	19.48431800	1.39380400	-0.57566700
C	18.10627200	1.28881400	-0.78214000
C	-14.78148700	-0.00709800	-0.66426900
C	-15.98712000	0.05703100	-0.51519400
C	-17.39930500	0.12684800	-0.34256500
C	-18.10659400	-0.95820400	0.20887100
C	-19.48432600	-0.88059100	0.36824900
C	-20.19896200	0.28213300	0.01030400
C	-19.49105700	1.36759000	-0.54793800
C	-18.10722400	1.27810000	-0.72047900

O	21.56264200	0.36895400	0.10555900
C	34.60944300	-0.32108600	7.90373500
C	33.08207300	-0.24055000	7.98347000
C	32.40374300	-0.21166700	6.60831400
C	30.87403600	-0.13139700	6.67854300
C	30.19679700	-0.10178100	5.30290400
C	28.66686600	-0.02217700	5.37264900
C	27.99119200	0.00933700	3.99628300
C	26.46111700	0.08770600	4.06462600
C	25.78845200	0.12234200	2.68704000
C	24.25801800	0.19858000	2.75432500
C	23.59544300	0.23801800	1.37137100
C	22.07815700	0.30376000	1.45438000
O	20.16408200	-1.96350200	0.80203800
C	28.63049700	-14.52422200	-0.63618700
C	27.98390800	-13.33540400	-1.35335900
C	27.21992500	-12.39965200	-0.40849900
C	26.56870100	-11.20653600	-1.11823900
C	25.80417100	-10.27187000	-0.17280600
C	25.15153600	-9.07943500	-0.88275500
C	24.38553600	-8.14646300	0.06316000
C	23.73050100	-6.95524100	-0.64665000
C	22.96234100	-6.02533600	0.30027000
C	22.30442100	-4.83589300	-0.41029200
C	21.53347800	-3.91486500	0.54358300
C	20.88716300	-2.74004300	-0.17419700
O	20.05934200	2.55861000	-0.99780300
C	26.80908000	16.02888500	1.04313300
C	26.75321200	14.55600100	1.45933400
C	25.67706900	13.75585700	0.71522700
C	25.61406300	12.27982600	1.12602700
C	24.53790000	11.48010300	0.38156700
C	24.47474700	10.00411700	0.79313500

C	23.39867500	9.20464400	0.04828200
C	23.33473400	7.72893000	0.46076900
C	22.25872700	6.93049200	-0.28494600
C	22.19418400	5.45510400	0.12930900
C	21.11600900	4.66374300	-0.62108500
C	21.06958200	3.20026300	-0.20313500
O	-20.15438900	-1.96389500	0.89800500
C	-28.39996000	-14.68013200	-0.47460300
C	-27.73121800	-13.50996800	-1.20206400
C	-27.02897100	-12.52891900	-0.25524300
C	-26.35664400	-11.35381600	-0.97527400
C	-25.65258900	-10.37436400	-0.02819600
C	-24.98047000	-9.19922100	-0.74871000
C	-24.27228800	-8.22252700	0.19813600
C	-23.60046200	-7.04741100	-0.52273000
C	-22.88599600	-6.07543900	0.42399300
C	-22.21501100	-4.90049200	-0.29835800
C	-21.49224400	-3.93953100	0.65386400
C	-20.83769000	-2.77730700	-0.07682600
O	-21.56927900	0.32862600	0.11325500
C	-34.73715700	-0.36104500	7.71118800
C	-33.21259700	-0.25376700	7.81078300
C	-32.51432300	-0.25133900	6.44534800
C	-30.98729400	-0.14454100	6.53574300
C	-30.28962900	-0.14127600	5.17003800
C	-28.76240600	-0.03521800	5.26086200
C	-28.06522400	-0.02999300	3.89491800
C	-26.53790500	0.07473000	3.98597500
C	-25.84201200	0.08310900	2.61956900
C	-24.31442100	0.18565800	2.71196400
C	-23.62618000	0.19870400	1.34109900
C	-22.11209700	0.29098700	1.45238100
O	-20.07287100	2.51252600	-1.01291100

C	-27.07919400	15.92128800	0.49570900
C	-27.00809600	14.46244100	0.95663300
C	-25.90442500	13.65874700	0.25807200
C	-25.82605000	12.19670300	0.71381700
C	-24.72222800	11.39349600	0.01501100
C	-24.64386100	9.93143000	0.47125000
C	-23.53990000	9.12864300	-0.22781100
C	-23.46103300	7.66669700	0.22878800
C	-22.35683100	6.86520800	-0.47101700
C	-22.27780800	5.40335200	-0.01352600
C	-21.17103900	4.60926300	-0.71799700
C	-21.11104300	3.15866300	-0.25891400
H	6.82637500	-5.97118300	0.21066700
H	4.39308700	-6.04437000	-0.00516200
H	3.15193300	-4.07498700	-0.75824300
H	2.19548000	-2.45101200	-1.04653300
H	2.18441300	1.49919400	-2.60665100
H	3.13001300	2.91429700	-3.45291700
H	4.36106300	4.87033300	-4.25286300
H	6.79908500	4.94955000	-4.10505200
H	8.68993600	4.97934000	-4.03236700
H	11.12822300	5.03035900	-3.87451400
H	12.34928600	3.15777200	-2.88274100
H	13.25052400	1.81724500	-1.88040800
H	13.25608700	-2.10528400	-0.25666000
H	12.35042700	-3.74279900	0.04934400
H	11.13347800	-5.76973000	0.67246100
H	8.70005300	-5.87075100	0.46687300
H	-8.62465600	-5.69514000	0.94965800
H	-11.04941800	-5.56694700	1.24113100
H	-12.28148400	-3.57656500	0.53903100
H	-13.22230700	-2.02636800	0.00427700
H	-13.23521100	1.85178900	-1.72088300

H	-12.33146300	3.18621300	-2.72659700
H	-11.11528000	5.04971000	-3.74062200
H	-8.67292800	5.02502900	-3.85317900
H	-6.77877300	5.08505700	-3.73689500
H	-4.33645000	5.03476700	-3.84108900
H	-3.10231600	3.05550000	-3.11127300
H	-2.15652700	1.53909400	-2.49323900
H	-2.16251300	-2.41855400	-0.95587600
H	-3.12574800	-4.02202300	-0.60554600
H	-4.36288900	-5.97526200	0.19282700
H	-6.78736200	-5.87211300	0.49420000
H	17.61298600	-1.89250200	0.35165100
H	17.58756300	2.14231400	-1.20231500
H	-17.59399300	-1.86602200	0.50458200
H	-17.59139600	2.12847200	-1.15043300
H	35.06294600	-0.33998800	8.90009300
H	35.02415600	0.53932200	7.36622700
H	34.93157100	-1.22539100	7.37489200
H	32.79175100	0.65574300	8.54844800
H	32.69981400	-1.09598100	8.55700600
H	32.78667900	0.64434400	6.03417100
H	32.69508600	-1.10836300	6.04254800
H	30.58360500	0.76497900	7.24505600
H	30.49196700	-0.98773500	7.25255100
H	30.57857800	0.75486100	4.72921400
H	30.48768500	-0.99781400	4.73613800
H	28.37565500	0.87323300	5.94019700
H	28.28464700	-0.87952100	5.94495600
H	28.37290300	0.86728600	3.42455300
H	28.28325600	-0.88548400	3.42825600
H	26.16851300	0.98152600	4.63388600
H	26.07863400	-0.77141400	4.63398500
H	26.16902100	0.98251000	2.11831000

H	26.08146200	-0.77042500	2.11673100
H	23.96503100	1.08982900	3.32700300
H	23.87653800	-0.66354600	3.31917300
H	23.96087100	1.10462100	0.80583200
H	23.87850400	-0.65139100	0.79451500
H	21.75579300	1.20029700	2.00290500
H	21.67049600	-0.57581300	1.96111200
H	27.87785800	-15.13703900	-0.12706700
H	29.16732100	-15.17206200	-1.33682300
H	29.34758100	-14.18778900	0.12131900
H	27.29855000	-13.70423900	-2.12863400
H	28.75750800	-12.76204800	-1.88217400
H	26.44624600	-12.97386400	0.12135300
H	27.90584600	-12.03081800	0.36772400
H	25.88367000	-11.57634600	-1.89459400
H	27.34286600	-10.63284000	-1.64771300
H	25.03057200	-10.84587500	0.35708000
H	26.48920000	-9.90139100	0.60321900
H	24.46710100	-9.44995600	-1.65922500
H	25.92490600	-8.50445000	-1.41190000
H	23.61305900	-8.72197400	0.59298200
H	25.06998500	-7.77480000	0.83911100
H	23.04694000	-7.32694200	-1.42325100
H	24.50258900	-6.37809300	-1.17524200
H	22.19076100	-6.60218000	0.82940800
H	23.64531600	-5.65134300	1.07611500
H	21.62335300	-5.21064400	-1.18723800
H	23.07572900	-4.25649200	-0.93703500
H	20.75094600	-4.48084700	1.06399100
H	22.20788800	-3.52924600	1.31873700
H	20.18514100	-3.09117700	-0.94374900
H	21.64002800	-2.10919800	-0.66286600
H	25.85418600	16.53141700	1.23505300

H	27.58590400	16.57176900	1.59140600
H	27.02479800	16.13054400	-0.02660300
H	26.57116400	14.48779900	2.54056200
H	27.73311100	14.08980000	1.28812700
H	24.69640900	14.22290400	0.88605600
H	25.85901000	13.82458800	-0.36698800
H	25.43235600	12.21216600	2.20825700
H	26.59515000	11.81381300	0.95535500
H	23.55688900	11.94622900	0.55190400
H	24.71977500	11.54732000	-0.70059800
H	24.29247300	9.93688100	1.87526400
H	25.45578000	9.53786500	0.62300800
H	22.41775500	9.67116900	0.21779700
H	23.58130400	9.27106800	-1.03376000
H	23.15137500	7.66246100	1.54276200
H	24.31563800	7.26212200	0.29146800
H	21.27778600	7.39670100	-0.11651900
H	22.44231500	6.99454200	-1.36667200
H	22.01031900	5.39193600	1.21141300
H	23.17517000	4.98873300	-0.03959000
H	20.12982300	5.11307100	-0.45147500
H	21.29998300	4.71074300	-1.70168400
H	20.81405800	3.11014100	0.86187400
H	22.03644200	2.71425000	-0.36419200
H	-28.89152300	-15.36121300	-1.17692800
H	-29.15969600	-14.32573600	0.23138200
H	-27.66760400	-15.26281900	0.09578600
H	-28.48301600	-12.96769700	-1.79162000
H	-27.00189900	-13.89784400	-1.92625400
H	-27.75878100	-12.14134200	0.47015700
H	-26.27681600	-13.07193000	0.33504100
H	-27.10955600	-10.81084600	-1.56442600
H	-25.62843800	-11.74242500	-1.70153300

H	-26.38036600	-9.98587700	0.69854000
H	-24.89933700	-10.91740600	0.56038200
H	-25.73405600	-8.65420500	-1.33502500
H	-24.25477300	-9.58785800	-1.47738400
H	-24.99727900	-7.83403900	0.92762900
H	-23.51809600	-8.76768000	0.78349000
H	-24.35511500	-6.49907600	-1.10449700
H	-22.87867200	-7.43610300	-1.25516600
H	-23.60618800	-5.68625100	1.15755200
H	-22.12974500	-6.62293200	1.00399300
H	-22.97216800	-4.34831600	-0.87272900
H	-21.49988700	-5.29063300	-1.03613200
H	-22.19935900	-3.54122000	1.39260100
H	-20.72067400	-4.47751600	1.21845200
H	-21.58254700	-2.17029900	-0.60629300
H	-20.10802500	-3.14121800	-0.81408300
H	-35.03711800	-1.28449500	7.20278700
H	-35.20510700	-0.36009900	8.70102200
H	-35.15727700	0.47760600	7.14429200
H	-32.82554500	-1.08701800	8.41303800
H	-32.94485400	0.66211500	8.35511800
H	-32.78302100	-1.16766000	5.90019800
H	-32.90201200	0.58247900	5.84245700
H	-30.60055600	-0.97873100	7.13851900
H	-30.71960400	0.77141600	7.08176900
H	-30.55764000	-1.05688700	4.62365400
H	-30.67586300	0.69330000	4.56753200
H	-28.37591700	-0.87051900	5.86216900
H	-28.49427300	0.87974500	5.80824300
H	-28.33393300	-0.94438500	3.34689300
H	-28.45084600	0.80603200	3.29407900
H	-26.15179600	-0.76248100	4.58477000
H	-26.26897800	0.98810400	4.53554200

H	-26.11089300	-0.82925900	2.06873800
H	-26.22572000	0.92152400	2.02124300
H	-23.93016300	-0.65475800	3.30685800
H	-24.04603600	1.09649100	3.26558100
H	-23.88390100	-0.71029400	0.78322000
H	-23.99382900	1.04395200	0.74548100
H	-21.70156700	-0.56825800	1.99066500
H	-21.81511900	1.20678900	1.98312500
H	-27.87559600	16.46706600	1.01209400
H	-27.27479900	15.98777200	-0.58063700
H	-26.13658800	16.44510600	0.69141700
H	-27.97671800	13.97514100	0.78010700
H	-26.84686400	14.42919500	2.04279500
H	-26.06551300	13.69250100	-0.82909300
H	-24.93504700	14.14688600	0.43422700
H	-26.79583200	11.70956000	0.53764700
H	-25.66534300	12.16400300	1.80097900
H	-24.88297200	11.42593900	-1.07208900
H	-23.75250200	11.88064900	0.19102000
H	-25.61353800	9.44409700	0.29522100
H	-24.48292800	9.89891700	1.55835300
H	-23.70095200	9.16071500	-1.31483200
H	-22.57029500	9.61604400	-0.05206600
H	-24.43052200	7.17894100	0.05293700
H	-23.29953100	7.63449000	1.31581500
H	-22.51819700	6.89558200	-1.55774600
H	-21.38722400	7.35205500	-0.29551400
H	-23.24731600	4.91623100	-0.18994000
H	-22.11655300	5.37372400	1.07360700
H	-21.33163900	4.62372200	-1.80324000
H	-20.19599300	5.07897100	-0.53944600
H	-22.06614700	2.65261100	-0.42809600
H	-20.87840900	3.10167400	0.81361300

12. Supporting Information References

1 M. J. Frisch, G. W. Trucks, H. B. Schlegel, G. E. Scuseria, M. A. Robb, J. R. Cheeseman, G. Scalmani, V. Barone, G. A. Petersson, H. Nakatsuji, X. Li, M. Caricato, A. V. Marenich, J. Bloino, B. G. Janesko, R. Gomperts, B. Mennucci, H. P. Hratchian, J. V. Ortiz, A. F. Izmaylov, J. L. Sonnenberg, D. Williams-Young, F. Ding, F. Lipparini, F. Egidi, J. Goings, B. Peng, A. Petrone, T. Henderson, D. Ranasinghe, V. G. Zakrzewski, J. Gao, N. Rega, G. Zheng, W. Liang, M. Hada, M. Ehara, K. Toyota, R. Fukuda, J. Hasegawa, M. Ishida, T. Nakajima, Y. Honda, O. Kitao, H. Nakai, T. Vreven, K. Throssell, J. A. Montgomery, Jr., J. E. Peralta, F. Ogliaro, M. J. Bearpark, J. J. Heyd, E. N. Brothers, K. N. Kudin, V. N. Staroverov, T. A. Keith, R. Kobayashi, J. Normand, K. Raghavachari, A. P. Rendell, J. C. Burant, S. S. Iyengar, J. Tomasi, M. Cossi, J. M. Millam, M. Klene, C. Adamo, R. Cammi, J. W. Ochterski, R. L. Martin, K. Morokuma, O. Farkas, J. B. Foresman, and D. J. Fox Gaussian 16, Revision C.02, Gaussian, *Inc., Wallingford CT*, **2016**.

2 A. D. Becke, *J. Chem. Phys.* **1993**, *98*, 1372.

3 M. M. Francl, W. J. Pietro, W. J. Hehre, J. S. Binkley, M. S. Gordon, D. J. DeFrees, J. A. Pople, *J. Chem. Phys.* **1982**, *77*, 3654.

4 A. P. Scott and L. Radom, *J. Phys. Chem*., **1996**, *41*, 16502.

5 G. Scalmani, M. J. Frisch, B. Mennucci, J. Tomasi, R. Cammi, V. Barone, *J. Chem. Phys.* **2006**, *124*, 094107.

6 R. E. Stratmann, G. E. Scuseria, M. J. Frisch, *J. Chem. Phys.* **1998**, *109*, 8218.

7 D. Casanova, M. Head-Gordon, *Phys. Chem. Chem. Phys.* **2009**, *11*, 9779.

8 K. Yamaguchi, *Self-Consistent Field: Theory and Applications* (R. Carbo, M. Klobukowsk, Eds.) Elsevier, **1990**, 727.

9 Y. Shao, Z. Gan, E. Epifanovsky, A. T.B. Gilbert, M. Wormit, J. Kussmann, A. W. Lange, A. Behn, J. Deng, X. Feng, D. Ghosh, M. Goldey, P. R. Horn, L. D. Jacobson, I. Kaliman, R. Z. Khaliullin, T. Kuś, A. Landau, J. Liu, E. I. Proynov, Y. Min Rhee, R. M. Richard, M. A. Rohrdanz, R. P. Steele, E. J. Sundstrom, H. L. Woodcock III, P. M. Zimmerman, D. Zuev, B. Albrecht, E. Alguire, B. Austin, G. J. O. Beran, Y. A. Bernard, E. Berquist , K. Brandhorst, K. B. Bravaya, S. T. Brown, D. Casanova, C.-M. Chang, Y. Chen, S. H. Chien, K. D. Closser, D. L. Crittenden, M. Diedenhofen, R. A. DiStasio Jr., H. Do, A. D. Dutoi, R. G. Edgar, S. Fatehi, L. Fusti-Molnar, A. Ghysels, A. Golubeva-Zadorozhnaya, J. Gomes, M. W.D. Hanson-Heine, P. H.P. Harbach, A. W. Hauser, E. G. Hohenstein, Z. C.

Holden, T.-C. Jagau, H. Ji, B. Kaduk, K. Khistyaev, J. Kim, J. Kim, R. A. King, P. Klunzinger, D. Kosenkov, T. Kowalczyk, C. M. Krauter, K. Un Lao, A. D. Laurent, K. V. Lawler, S. V. Levchenko, C. Yeh Lin, F. Liu, E. Livshits, R. C. Lochan, A. Luenser, P. Manohar, S. F. Manzer, S.-P. Mao, N. Mardirossian, A. V. Marenich, S. A. Maurer, N. J. Mayhall, E.Neuscamman, C. M. Oana, R. Olivares-Amaya, D. P. O'Neill, J. A. Parkhill, T. M. Perrine, R. Peverati, A. Prociuk, D. R. Rehn, E. Rosta, N. J. Russ, S. M. Sharada, S. Sharma, D. W. Small, A. Sodt, T. Stein, D. Stück, Y.-C. Su, A. J.W. Thom, T. Tsuchimochi, V. Vanovschi, L. Vogt, O. Vydrov, T. Wang, M. A. Watson, J. Wenzel, A. White, C.F. Williams, J. Yang, S. Yeganeh, S.R. Yost, Z.-Q. You, I. Ying Zhang, X. Zhang, Y. Zhao, B. R. Brooks, G. K.L. Chan, D. M. Chipman, C. J. Cramer, W. A. Goddard III, M.S. Gordon, W. J. Hehre, A. Klamt, H. F. Schaefer III, M. W. Schmidt, C. D. Sherrill, D. G. Truhlar, A. Warshel, X. Xu, A. Aspuru-Guzik, R. Baer, A. T. Bell, N. A. Besley, J.-D. Chai, A. Dreuw, B. D. Dunietz, T. R. Furlani, S. R. Gwaltney, C.-P. Hsu, Y. Jung, Ji. Kong, D.S. Lambrecht, W. Liang, C. Ochsenfeld, V. A. Rassolov, L. V. Slipchenko, J. E. Subotnik, T. Van Voorhis, J. M. Herbert, A. I. Krylov, P. M.W. Gill, M. Head-Gordon, *Mol. Phys.* **2015**, *113*, 184.

10 D. Doehnert, J. Koutecký, *J. Am. Chem. Soc.* **1980**, *102*, 1789.

11 C. Schierl, W. Alex, L. M. Mateo, B. Ballesteros, D. Shimizu, A. Osuka, T. Torres, D. M. Guldi, G. Bottari, *Angew. Chem. Int. Ed. Engl.* **2019,** *58*, 14644-14652.

12 C. Schierl, V. Pardo, G. Bottari, D. M. Guldi, T. Torres, *ECS J. Solid State Sci. Technol.* **2020,** *9*, 051011.

13 J. Wu, M. D. Watson, L. Zhang, Z. Wang, K. Müllen, *J. Am. Chem. Soc.* **2004,** *126*, 177-186.

14 C. J. F. Du, H. Hart, K. K. D. Ng, *J. Org. Chem.* **1986,** *51*, 3162-3165.

15 D. V. Yandulov, R. R. Schrock, A. L. Rheingold, C. Ceccarelli, W. M. Davis, *Inorg. Chem.* **2003,** *42*, 796-813.

16 A. J. Markvoort, H. M. ten Eikelder, P. A. Hilbers, T. F. de Greef, E. W. Meijer, *Nat. Commun.* **2011,** *2*, 509.

17 H. M. ten Eikelder, A. J. Markvoort, T. F. de Greef, P. A. Hilbers, *J. Phys. Chem. B* **2012,** *116*, 5291-301.

18 C. Rest, R. Kandanelli, G. Fernández, *Chem. Soc. Rev.* **2015,** *44*, 2543-2572.
